# Immobilized Activation Base for Solid-Phase Peptide Synthesis in Flow

**DOI:** 10.1021/acs.joc.6c00252

**Published:** 2026-05-02

**Authors:** Anna Wettler, Bálint Tamás, Nina Hartrampf

**Affiliations:** Department of Chemistry, 27217 University of Zurich, Winterthurerstrasse 190, Zurich 8057, Switzerland

## Abstract

The chemical synthesis of peptides and proteins heavily relies on solid-phase peptide synthesis. Despite key advances, especially in flow-based methods, issues with aggregation and base-promoted side reactions persist. Higher temperatures would reduce aggregation but aggravate side reactions. To address this issue, we confined the basic environment to the amino acid activation step by developing a flow system featuring a reusable, immobilized amine base. After effective coupling conditions with minimal epimerization for each canonical amino acid were identified, we confirmed the viability of higher temperatures in the coupling step in the absence of excess base by synthesizing four test peptides. Finally, the synthesis of aggregation-prone peptides, where our method proved on par or superior to standard protocols, was achieved. This highlights the advantages of spatially separating both the peptide chain and reagents through immobilization.

## Graphical abstract

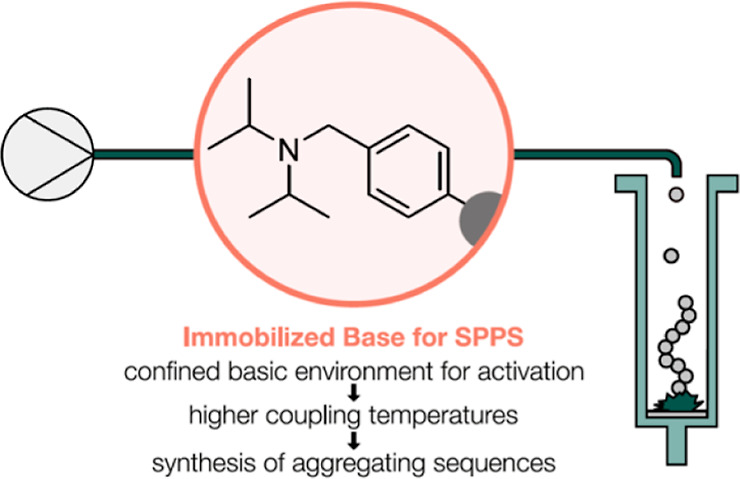

## Introduction

Solid-phase peptide synthesis (SPPS) is a key method for the chemical synthesis of peptides and proteins.^1^ The iterative peptide bond formation/deprotection cycles on resin substantially benefit from the simplified removal of excess reagents, reactants and soluble byproducts. Advances in flow-SPPS allowed the precise control of temperature and residence time, reducing reaction times while minimizing side reactions and accumulation of byproducts.^2^ Under highly optimized conditions,^3^ sequences exceeding 100 amino acids became accessible in hours of synthesis time.^4^ The high temperatures employed during automated fast-flow peptide synthesis (AFPS) reduced, but did not eliminate aggregation during synthesis.^5^ One straightforward solution, namely higher coupling temperatures, is prohibited by increasing side reactions, especially under basic conditions. The coupling base, essential for active ester formation, promotes epimerization (Figure 1A),^6,7^ while aspartimide formation mostly occurs during the deprotection step.^8–10^ Confining the basic environment to the activation step should enable increased coupling temperature without an increase in epimerization-related side products.

**Figure 1:**
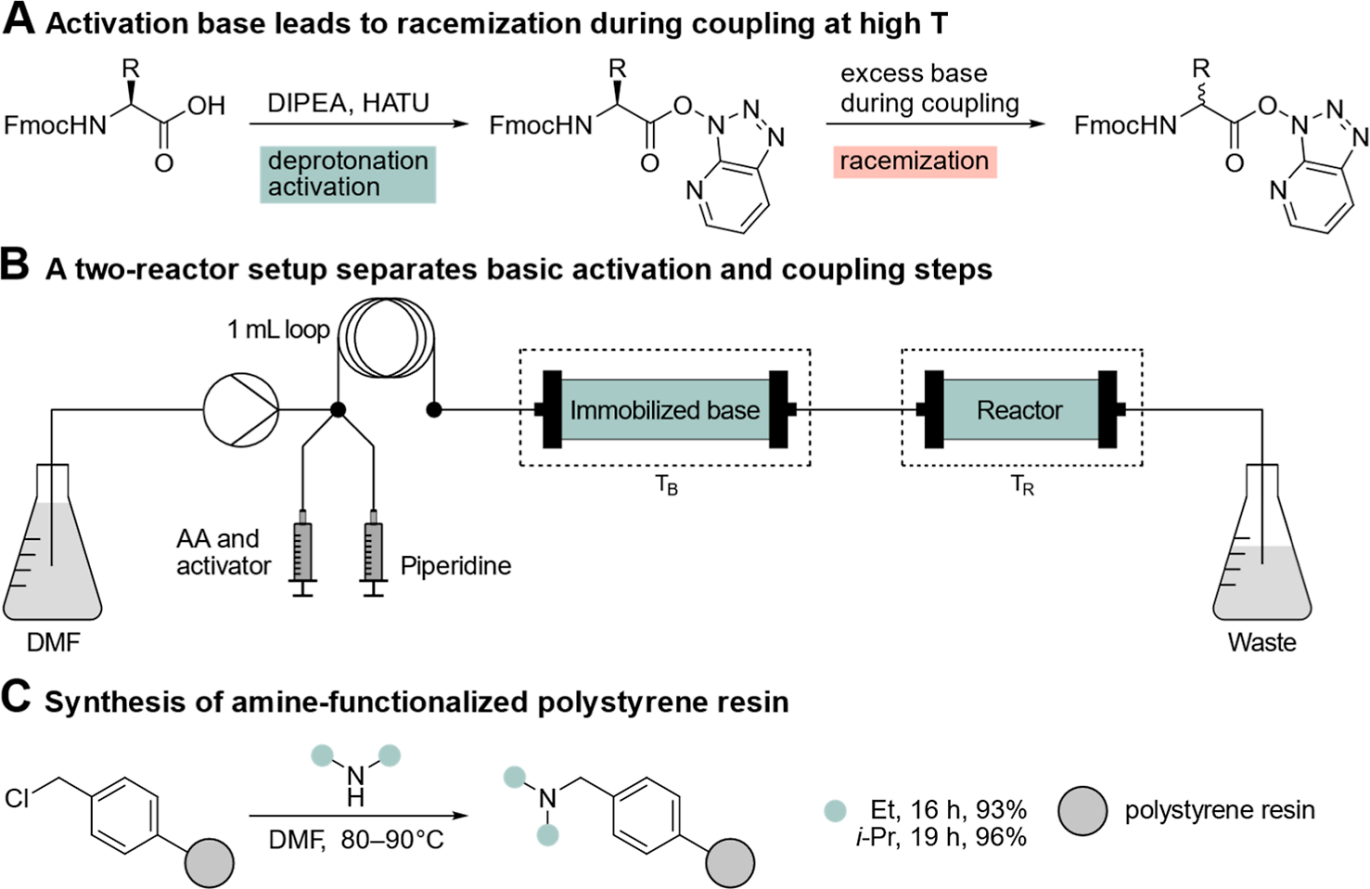
An immobilized base has the potential to reduce racemization at elevated synthesis temperatures. (A) The tertiary amine base DIPEA promotes the activation step, but an unwanted racemization can occur during prolonged base exposure. (B) Schematic representation of the flow system used: The loop enables precise addition of the amino acid and piperidine solutions, while separate heating baths allow for the independent temperature optimization of the activation and coupling steps. (C) Synthesis of the immobilized base using Merrifield resin and secondary amines.

Such an approach would make use of immobilized reagents, whose use in liquid-phase peptide synthesis (LPPS) is well-described.^11^ While the focus has been on immobilized activators, polymer-supported bases such as PS*-N-*methylaminopyridine or PS-morpholine were also developed and used in amide couplings as general bases or nucleophilic catalysts.^12–16^ While the use of immobilized reagents for the synthesis of short peptides has been demonstrated, their application has been mostly limited to LPPS,^17^ with the notable exception of Wei et al.’s immobilized activators for SPPS.^18^

Herein we report an approach that combines SPPS with the immobilization of the activation base DIPEA for HATU-^19^ and PyAOP-mediated^20^ amide coupling. The use of a flow system enables two consecutive solid-supported reactions by the swift solution transfer between reactor beds (Figure 1B). This approach achieves spatial separation of activation and coupling while eliminating the base from the coupling step and should thus allow for higher coupling temperatures.

## Results

The base-functionalized resins were synthesized using Merrifield resin and the secondary amines diethylamine (DEA) and diisopropylamine (DIPA). The DEA-functionalized resin was prepared following the procedure reported by Esteve et al.^16^ and adapted for DIPA by extending the reaction time from 16 to 19 h (Figure 1C). Complete substitution was confirmed by disappearance of the characteristic C–Cl IR stretch at 1264 cm^–1^. To further confirm functionalization and assess the basicity of the resins, we performed a 4-(4-nitrobenzyl)­pyridine (NBP) test^16,21^ (Supporting Information Figure S5) and used the pH indicator bromophenol blue^22^ (Supporting Information Figure S6). The basic resins were packed into empty HPLC columns, and the equivalents were calculated based on the nominal loading of the functionalized resin.

Next, the immobilized base was implemented into a flow-SPPS system to evaluate its performance in amino acid activation. The setup features an injection loop for precise control over amino acid and piperidine solution volumes (Figure 1B). The reagents are delivered by a HPLC pump and sequentially pass through a column containing the immobilized base, then into the reactor packed with the peptide resin. In the piperidine-mediated Fmoc deprotection step, the immobilized base is regenerated concomitantly. In fast-flow SPPS, DIPEA is usually the reagent used in largest excess, e.g. 0.6 mL DIPEA for each amino acid coupling on AFPS,^3^ which we eliminate with the use of the immobilized base.

The immobilized base resins were evaluated regarding conversion and epimerization. Resin preloaded with LYRAG pentamer was used to improve solubility and facilitate HPLC analysis (Figure 2A). Conversion was measured as ratio of XXX-LYRAG to LYRAG. For model sequence ALF, excellent crude purities were achieved with DEA- and DIPA-functionalized resin (98% and >99%, Figure 2B). We then evaluated the epimerization-prone histidine (H) and cysteine (C) using test sequences GCF and FHL.^3^ With DEA, GCF showed >99% conversion and 73% all-l, while FHL resulted in 81% conversion with 83% all-l. The DIPA-functionalized resin led to reduced epimerization: for GCF >99% conversion with no detectable d-epimer was observed, and FHL gave 77% conversion with an all-l content of 92% (Figure 2B). Based on these results, we selected the DIPA-functionalized resin for further optimization studies.

**Figure 2:**
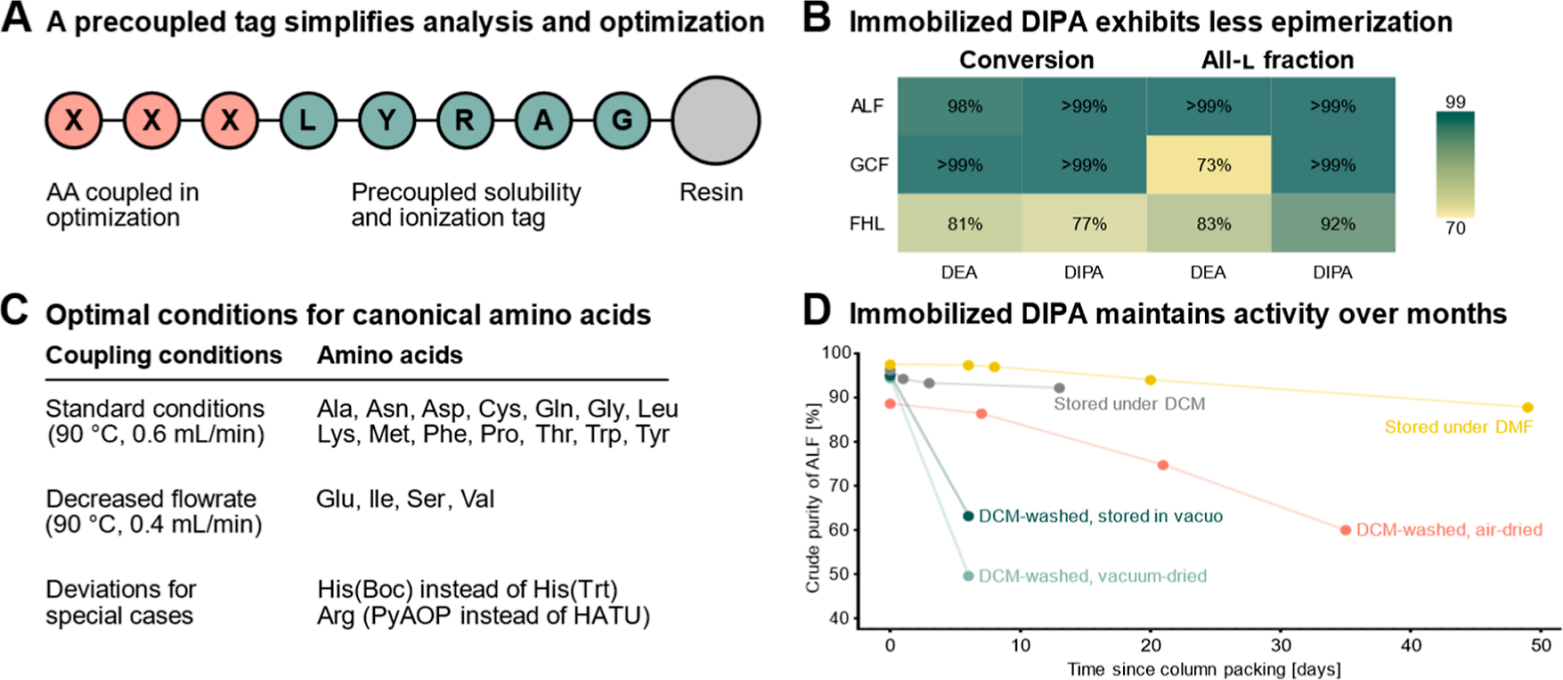
Immobilized DIPA enables amino acid activation and coupling under optimized conditions. (A) During optimization, three amino acids are coupled onto an existing LYRAG tag, which facilitates ionization and analysis. (B) Comparison between DEA- and DIPA-functionalized resin, using a simple test sequence (ALF) and two sequences containing epimerization-prone cysteine (GCF) and histidine (FHL). (C) Optimized conditions for the 20 canonical amino acids, only His and Arg require bespoke conditions. (D) Storage in DMF provides best reagent stability, enabling over a month usage time and more than 200 couplings with minimal loss of efficacy; dry storage results in quick degradation.

The coupling conditions for all 20 canonical amino acids were then optimized by synthesizing random sequences of three or four amino acids onto LYRAG on resin (Figure 2C). The conditions initially optimized for the ALF sequence (0.6 mL/min, 90 °C, 5 eq. AA, 4.9 eq. activator, deprotection 5 mL 20% pip with 10 mL/min) were used as a starting point for all other amino acids, seven of which required further optimization (glutamic acid (E), histidine (H), isoleucine (I), methionine (M), arginine (R), serine (S), and valine (V)). For H, it was found that switching from H­(Trt) to H­(Boc) allowed for slower flow rates and higher activation temperatures, resulting in >99% conversion without detectable epimerization. For M, the S-oxidation side product increased with sequence length, which could be mitigated by degassing the solvent. In the case of E, I, S, and V, partial deletions could be suppressed by reducing the flow rate from 0.6 to 0.4 mL/min. For R, adjusting the flow rate did not improve coupling efficiency, but switching the activator from HATU to PyAOP led to 10% improvement in conversion. To evaluate the difficult couplings between hydrophobic, β-branched residues, we tested the LIV tripeptide on the LYRAG scaffold, achieving >99% crude purity. Finally, we optimized piperidine consumption during the washing and deprotection steps, respectively (see Supporting Information Section 8).

Next, we assessed the stability of the DIPA-functionalized resin by repeatedly using it for synthesizing the ALF-LYRAG test sequence over the course of up to 50 days and monitored crude purity over time under various storage conditions (Figure 2D): under vacuum, under air, and in DCM or DMF. Storage in an evacuated desiccator led to a rapid decline in performance, with crude purities dropping from >90% to 50–60% within a week. Air storage resulted in a more gradual decline, reaching ∼60% crude purity after one month. In contrast, storing the functionalized resin in solvent preserved its activity. As both solvents showed similar stability after 15 days, we proceeded with DMF, our standard SPPS solvent. The DMF-stored resin retained high efficiency for over 50 days, showing only a minor decrease in crude purity. Within this 50-day period, the resin was used for more than 200 coupling/deprotection cycles. In addition, storage in DMF eliminated the need for solvent exchange steps before and after each synthesis. Furthermore, when stored at −20 °C, synthesis performance did not drop significantly even after a year.

With optimized coupling and storage conditions in hand, we finally evaluated our immobilized base in the synthesis of peptide sequences (Figure 3). Three test cases were selected: the LYRAG tag, a 16-mer fragment of microprotein NBDY[53–68], and a 12-mer fragment of HIV-1 protease [88–99]. The LYRAG tag was selected as it includes arginine, one of the most challenging amino acids to couple. The previously optimized conditions resulted in 63% crude purity, the main impurity being R deletion. Repeating the R coupling step increased the crude purity to 85%. For NBDY[53–68] a crude purity of 68% was obtained, with a C-terminal lysine deletion as the major side product. The synthesis of HIV-1 protease [88–99] under standard conditions yielded a crude purity of 50%. Overall, these results demonstrate the applicability of DIPA-functionalized resin for peptide synthesis. Notably, aspartimide formation—a common side-reaction in SPPS—persists, as it is mainly influenced by the deprotection base (see Supporting Information Section 13).

**Figure 3:**
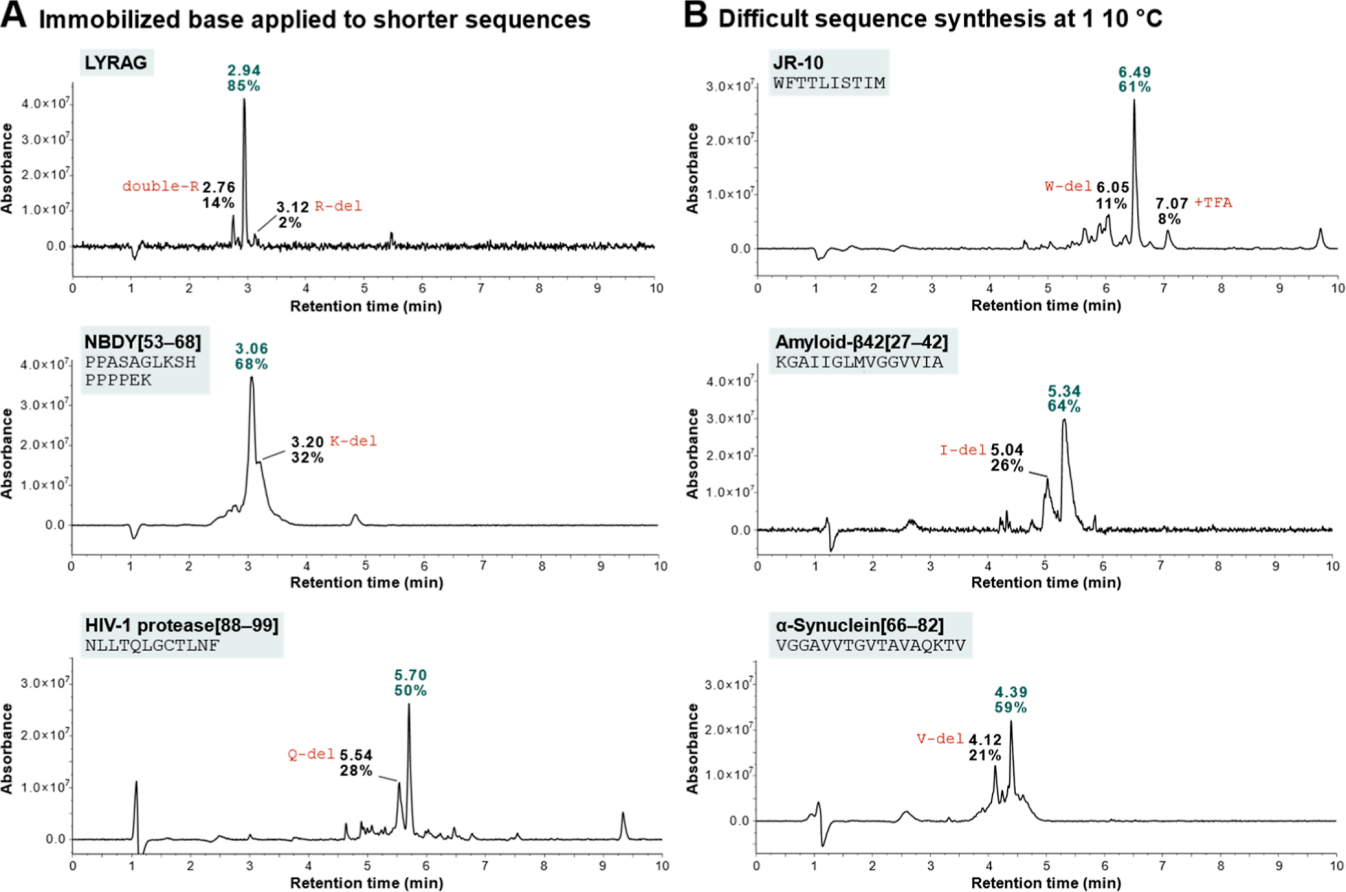
Applications of the immobilized base in peptide synthesis. (A) Synthesis of short peptides. (B) The use of immobilized base improves the crude purity of aggregating sequences at 110 °C.

As the initial motivation for this study was to develop a platform for the synthesis of aggregation-prone peptides, we next explored higher coupling temperatures. These would not only improve coupling rates but also reduce β-sheet formation by increasing the entropic penalty associated with ordered structures.^24^ Before proceeding with the syntheses, the effect of temperature was tested on the epimerization-prone GCF and FH­(Boc)­L sequences. No epimerization was detected at 100 °C, and approximately 5% (GCF) and 7% (FHL) epimerized product at 110 °C (Supporting Information Section 12).

To test the effect of increased temperature on aggregation, we selected three known aggregation-prone sequences, JR-10, amyloid-β[27–42], and α-synuclein[66–82], and compared synthesis outcome to previously reported syntheses of these peptides from our lab (Figure 3B). As benchmark, JR-10 was synthesized at 90 °C with a crude purity of approximately 58%. While increasing the temperature to 100 °C led to less aggregation-related F and W deletion side products, the overall crude purity remained unchanged (54%) due to an increase in M oxidation (18%), which was exacerbated at 110 °C (53%). Solvent degassing improved purity to a similar 61% at 110 °C, which matches the literature value.^25^ Amyloid-β[27–42] was synthesized with a crude purity of 47% at 90 °C, raising the temperature to 110 °C improved the crude purity to 55%. By degassing the solvent, the main M-oxidation side product could again be minimized, resulting in a 64% crude purity, which is a significant improvement compared to 42% in the literature.^25^ Finally, α-synuclein[66–82] was synthesized with crude purities of approximately 40% at 90 °C and 59% at 110 °C, matching or considerably outperforming the literature result (37%^25^). In summary, the use of an immobilized base at elevated temperatures is also effective for aggregation-prone sequences, yielding crude purities at least comparable to those reported in the literature.

## Conclusion

In this study, we demonstrated the potential of immobilized bases for the activation of amino acids in flow-SPPS. Both DEA- and DIPA-derived resins provided very high conversions; however, the DIPA-functionalized resin outperformed DEA in reducing epimerization for H and C. Under optimal storage conditions, the same DIPA-functionalized resin can be reused for more than 200 coupling steps over a period of nearly two months and could be stored in a freezer for over a year without notable decrease in performance. Optimizing the coupling conditions for all 20 canonical amino acids resulted in >95% conversion for each amino acid coupling, and the synthesis of seven short to medium-length peptides was achieved. These included the aggregation-prone sequences JR-10, amyloid-β[27–42], and α-synuclein[66–82], which were obtained in crude purities matching or surpassing those reported in the literature. In summary, this work demonstrates the potential of combining immobilizing reagents with flow-SPPS, allowing for the spatial separation of reaction steps and greener processes through reagent recycling.

## Supplementary Material



## Data Availability

The data underlying this study are available in the published article, in its Supporting Information, and openly available in Zenodo: https://zenodo.org/doi/10.5281/zenodo.19653949

## Notes

Methods and materials, experimental procedures, analytical data (PDF)
