# Supplementary material for: Immobilized Activation Base for Solid-Phase Peptide Synthesis in Flow
Source: J Org Chem. 2026 May 2;91(19):6641–5. doi: 10.1021/acs.joc.6c00252 (PMC13185110; doi:10.1021/acs.joc.6c00252)
Supplement: Supplementary file 1 [file jo6c00252_si_001.pdf]

Supplementary material

Immobilized Activation Base for Solid-Phase Peptide Synthesis in Flow

Authors: Anna Wettler[†], Bálint Tamás[†], and Nina Hartrampf[*]

Affiliation:

Department of Chemistry, University of Zurich, Winterthurerstrasse 190, 8057 Zurich, Switzerland

* Correspondence to: nina.hartrampf@chem.uzh.ch

† Authors contributed equally

Table of Contents

1 Methods and materials S4

1.1 Reagents and solvents S4

1.2 Automated flow-based peptide synthesis (AFPS) S4

1.3 Peptide cleavage and deprotection S5

1.4 Batch Synthesis S5

2 Analytical methods S5

2.1 Liquid Chromatography with High-Resolution Electrospray Ionization Mass Spectrometry (LC–MS) S5

2.2 Analytical Ultra-High Performance Liquid Chromatography (UHPLC) S6

2.3 Infrared Spectroscopy (IR) S6

3 Synthesis of the LYRAG tags using the AFPS S6

3.1 Synthesis 1 ..... S6

4 Synthesis of DEA- and DIPA-functionalized resins S7

4.1 Diethylamine-functionalized immobilized base (DEA) S7

4.2 Diisopropylamine-functionalized immobilized base batch 1 (DIPA b.1) S8

4.3 Diisopropylamine-functionalized immobilized base batch 2 (DIPA b.2) S9

4.4 IV NBP test S9

4.5 Basicity test with bromophenol blue S10

5 Experimental setup S11

5.1 Flow setup S11

5.2 Reactor S12

6 Immobilized base-SPPS S12

7 Comparison of DEA- and DIPA-functionalized resins S13

7.1 Coupling of ALF to the LYRAG-coupled resin S13

7.2 Coupling of GCF to the LYRAG coupled resin S16

7.3 Coupling of FHL to the LYRAG coupled resin S20

8 Stability investigation of the DIPA-functionalized resin S25

8.1 Washed with DCM, dried and stored airtight S25

8.2 Washed with methanol and DCM, dried and stored in vacuo S29

8.3 Washed with DCM, stored in vacuo S31

8.4 Washed with DCM and stored airtight S33

8.5 Washed with DMF and stored airtight S37

8.6 Storage sealed in -20°C freezer S42

9 Optimization of all canonical amino acids S43

9.1 A, F, L S43

9.2 C, G S43

9.3 D, E S44
9.4 H S45
9.5 I, V S46
9.6 K, M, N S48
9.7 P, Q, R, S S49
9.8 T, W, Y S52
10 Piperidine reduction S53
10.1 0.3 mL piperidine S53
10.2 0.6 mL piperidine S54
11 Synthesis of peptides using the DIPA-functionalized base as catalyst S55
11.1 LYRAG S55
11.2 NBDY [53–68] S57
11.3 HIV-1 protease [88–99] S59
12 Test of the effect of temperature on epimerization sensitive amino acids cysteine and histidine S63
12.1 GCF added to LYRAG tag at 100 °C S63
12.2 GCF added to LYRAG tag at 110 °C S64
12.3 FHL added to LYRAG tag at 110 °C S66
13 Synthesis of aspartimide formation prone sequence NN92[2–29] S67
13.1 Synthesis of NN92[15–29] on the AFPS S67
13.2 Standard synthesis of NN92[2–14] onto resin-bound NN92[15–29] S68
14 Synthesis of aggregating sequences S70
14.1 JR-10 ..... S70
14.2 Amyloid-β42 [27–42] S78
14.3 α-synuclein [66–82] S84
15 References S89

1 Methods and materials

1.1 Reagents and solvents

Fmoc- and side chain-protected L-amino acids (Fmoc-Ala-OH, Fmoc-Arg(Pbf)-OH, Fmoc-Asn(Trt)-OH, Fmoc-Asp(Ot-Bu)-OH, Fmoc-Cys(Trt)-OH, Fmoc-Gln(Trt)-OH, Fmoc-Glu(Ot-Bu)-OH, Fmoc-Gly-OH, Fmoc-His(Trt)-OH, Fmoc-Ile-OH, Fmoc-Leu-OH, Fmoc-Lys(Boc)-OH, Fmoc-Met-OH, Fmoc-Phe-OH, Fmoc-Pro-OH, Fmoc-Ser(tBu)-OH, Fmoc-Thr(t-Bu)-OH, Fmoc-Trp(Boc)-OH, Fmoc-Tyr(t-Bu)-OH, Fmoc-Val-OH) and N'-tetramethyluronium hexafluorophosphate (HATU) were purchased from Bachem; O-(7-azabenzotriazol-1-yl)-N,N,N' and (7-azabenzotriazol-1-yloxy)tripyrrolidinophosphonium hexafluorophosphate (PyAOP) were purchased from Advanced ChemTech; Diethylamine (DEA >99.5%) was purchased from Sigma Alrich ; Diisopropylamine (DIPA, 99%) was purchased from PanReac AppliChem ; Bromophenol blue was purchased fomr Sigma Aldrich ; Merrifield resin was purchased from Sigma Aldrich ; N,N-diisopropylethylamine (i-Pr_2NEt, DIPEA, 99.5%) was purchased from Sigma-Aldrich; trifluoroacetic acid (TFA, for HPLC, ≥99.0%), triisopropylsilane (TIPS, 98%) and 3,6-dioxa-1,8-octane-dithiol (DODT, 95%) were purchased from Sigma-Aldrich. N,N-Dimethylformamide (DMF) was purchased from the from VWR nternational GmbH; dichloromethane (DCM, ≥99.8%) was purchased from Fisher Scientific Ltd.; diethyl ether was purchased from Honeywell Riedel-de Haën; acetonitrile (MeCN, for HPLC gradient grade, ≥99.9%) was purchased from Sigma-Aldrich. NovaPEG Rink Amide resin (0.41 or 0.20 mmol/g loading) was purchased from the Novabiochem-line from Sigma-Aldrich Canada Ltd. Piperidine (>99%, for synthesis) was purchased from Carl Roth GmbH. Formic acid (reagent grade, >95%) and AldraAmine trapping agent added to DMF were purchased from Sigma-Aldrich Canada Ltd.

1.2 Automated flow-based peptide synthesis (AFPS)

Peptides were synthesized on an automated flow system built in the Hartrampf lab, which is similar to the published AFPS system.[S1] Capitalized letters refer to L-amino acids. Unless otherwise noted, the following settings were used for peptide synthesis: flow rate = 20 mL/min for coupling and deprotection steps, temperature = 90 °C (loop) for all canonical amino acids except histidine and cysteine, those were coupled on room temperature and 90 °C (reactor). The standard synthetic cycle involves a first step of prewashing the resin at 90 °C for 60 s at 40 mL/min. During the coupling step, three HPLC pumps are used: a 50 mL/min pump head pumps the activating agent, a second 50 mL/min pump head pumps the amino acid, and a 5.0 mL/min pump head pumps *i*-Pr_2NEt (*neat*). The 50 mL/min pump head pumps delivered 0.398679 mL of liquid per pump stroke, the 5.0 mL/min pump head pumps 3.9239×10^{-2} mL of liquid per pump stroke.

All peptides were prepared by AFPS on NovaPEG Rink Amide resin (0.41 or 0.20 mmol/g) and standard Fmoc/*t*-Bu protected amino acids (0.40 M in DMF) were coupled using HATU (0.38 M in DMF) or PyAOP (0.38 M in DMF) with DIPEA (neat, 3.0 mL/min) at a total flow rate of 20 mL/min. For amino acids D, E, F, G, I, K, L, M, P, S, W, and Y, a total volume of 6.4 mL of the "coupling solution" (i.e. amino

acid (0.20 M), HATU or PyAOP (0.19 M), and DIPEA in DMF) was applied for each coupling. For amino acids A, C, H, N, Q, R, S, T, and V, a total of 10.4 mL of "coupling solution" was applied for each coupling. All amino acids except C and H were preheated at 90 °C during the activation step with HATU or PyAOP, whereas C and H were preactivated with PyAOP at room temperature. Removal of the N^{α}-Fmoc group was achieved using 20% piperidine with 1% formic acid in DMF at a flow rate of 20 mL/min and a total volume of 6.4 mL at 90 °C. Between each coupling and deprotection step, the resin was washed with DMF (32 mL) at 90 °C with a flow rate of 40 mL/min. After completion of the peptide sequence, the resins were manually washed with DCM (3 × 5 mL) and dried under reduced pressure.

1.3 Peptide cleavage and deprotection

All peptides were cleaved using a solution of TFA/TIPS/DODT/H_2O (94:1:2.5:2.5, *v*/*v*/*v*/*v*, 1–3 mL) for 2 h at room temperature with gentle mixing. TFA was then removed by evaporation under a light stream of N_2, and the peptides were precipitated and isolated by centrifugation from ice-cold diethyl ether (14–45 mL), twice. The resulting peptide pellets were then briefly dried under a light stream of N_2, then dissolved in 2 mL of an aqueous solution containing 50% MeCN and 0.1 % TFA and lyophilized. Crude peptides were then analyzed by LC–MS and UHPLC (214 nm). Pure peptide samples were obtained using RP-HPLC and were analyzed for purity by LC–MS and UHPLC (214 nm).

1.4 Batch Synthesis

Unless otherwise noted, pre-functionalized NovaPEG Rink Amide resin (0.41 or 0.20 mmol/g loading) was used in all experiments. The amino resin (1 eq., 21 µmol.) was swelled with DCM (1 × 5 mL) for 1 min. and washed with DMF (1 × 5 mL), then the solvent was removed by filtration under reduced pressure. For each coupling: a solution of Fmoc- and sidechain-protected amino acid (0.5 mL, 0.2 M in DMF, 5.0 eq.) and HATU (0.5 mL, 0.19 M in DMF, 4.8 eq.) was prepared. To this solution, DIPEA (10 eq.) was added, and the solution was gently agitated at 23 °C for 1 min. The solution was then added to the resin, and the reaction was gently stirred for 20 sec., then left to react at 23 °C for 30 min. Afterwards, the resin was drained, washed with DMF (3 × 5 mL) and DCM (3 × 5 mL). For each deprotection: 2 mL of solution of 20% piperidine in DMF (*v*/*v*) was then added to the reactor, the resin was gently stirred for 20 sec. then left to react at 23 °C for 20 min. Afterwards, the resin was drained, then washed with DMF (3 × 5 mL) and DCM (3 × 5 mL).

2 Analytical methods

2.1 Liquid Chromatography with High-Resolution Electrospray Ionization Mass Spectrometry (LC–MS)

For determination of peptide masses and purity by LC–MS, the filtered peptide solution was diluted in 10–50% acetonitrile (MeCN) in water with 0.1% TFA (60–500 µL) to a final concentration of approximately 0.1 mg/mL. The samples were analyzed on an Acquity UPLC (Waters, Milford, USA) connected to an Acquity el diode array detector and a Synapt G2HR-ESI-QTOF-MS (Waters, Milford, USA).

For standard analysis of all peptide samples, LC–MS spectra were recorded on an Acquity BEH C8 HPLC column (2.1 × 100 mm, 1.7 µm particle size, Waters) kept at 30 °C at a flow rate of 0.4 mL/min with UV detection at 190–300 nm. A binary solvent system was used, wherein Solvent A was water containing 0.02% formic acid and 0.04% TFA, and Solvent B was MeCN containing 0.04% formic acid and 0.02% TFA. The LC–MS gradient used was as follows: isocratic at 3% Solvent B for 3 min, then linear gradient of 3–95% Solvent B over 9 min, followed by isocratic at 95% Solvent B for 1 min.

UV spectra recorded at 1.2 nm resolution and 20 points s^{-1}; ESI: positive ionization mode, capillary voltage 3.0 kV, sampling cone 40V, extraction cone 4V, N_2 cone gas 4 L h^{-1}, N_2 desolvation gas 800 L min^{-1}, source temperature 120 °C; mass analyzer in resolution mode: mass range 150–3000 *m/z* with a scan rate of 1 Hz; mass calibration to <2 ppm within 50–2500 *m/z* with a 5.0 mM aq. soln. of HCO_2Na, lock masses: *m/z* 195.0882 (caffein, 0.7 ng mL^{-1}) and 556.2771 (Leucine-enkephalin, 2 ng mL^{-1}).

All mass spectra show deconvoluted masses from the raw *m/z* values, calculated using Mestrelab Research S.L.© MestReNova v. 14.1 Mnova MS Suite. Purity based on LC–MS was calculated by calculating the Area Under the Curve (AUC) of desired product peak as a percentage of the AUC of all peaks (within 2–8 min) of the absorbance chromatogram (λ = 214 nm).

2.2 Analytical Ultra-High Performance Liquid Chromatography (UHPLC)

For determination of peptide masses by UHPLC, the filtered peptide solution was diluted in 10–50% acetonitrile (MeCN) in water with 0.1% TFA (500 µL) to a final concentration of approximately 0.1 mM. The samples were analyzed on an Agilent 1290 Infinity II Series UHPLC, which is connected to an Agilent 1260 Infinity II Series VWD, and an Agilent 6546 LC/Q-TOF. Separation was carried out on an Agilent Poroshell 300SB-C8 HPLC column (5 µm particle size, 2.1 × 75 mm) which was at 50 °C, with a sample injection volume of 5 µL. The elution was performed at a flow rate of 0.8 mL/min with solvent A: H2O + 0.1% formic acid and solvent B: MeCN + 0.1 formic acid with the following UHPLC gradient: gradient: isocratic at 5% Solvent B for 1.5 min, followed by a linear gradient of 5–95% Solvent B over 5 min, followed by isocratic at 95% Solvent B for 1 min.

2.3 Infrared Spectroscopy (IR)

SpectrumTwo FT-IR Spectrometer (Perkin–Elmer) equipped with a Specac Golden GateTM ATR (attenuated total reflection) accessory; applied as neat samples; 1/λ in cm–1. Peaks are labeled with w (weak), m (medium) or s (strong).

3 Synthesis of the LYRAG tags using the AFPS

3.1 Synthesis 1

The sequence LYRAG was synthesized on commercially available Novabiochem® NovaPEG Rink Amide resin (0.41 mmol/g, 202 mg, 83 µmol) using the standard AFPS protocol (SI 1.2). Total synthesis time to afford resin-bound LYRAG was approximately 0.25 h. Cleavage of the peptidyl resin (approx. 10 mg, 4.1

μmol) according to Cleavage Protocol (Section 1.3) afforded the crude peptide as a colorless solid (79% purity by LCMS [SI Figure 1]). The synthesis was carried out several times, the analytical data looks similar.

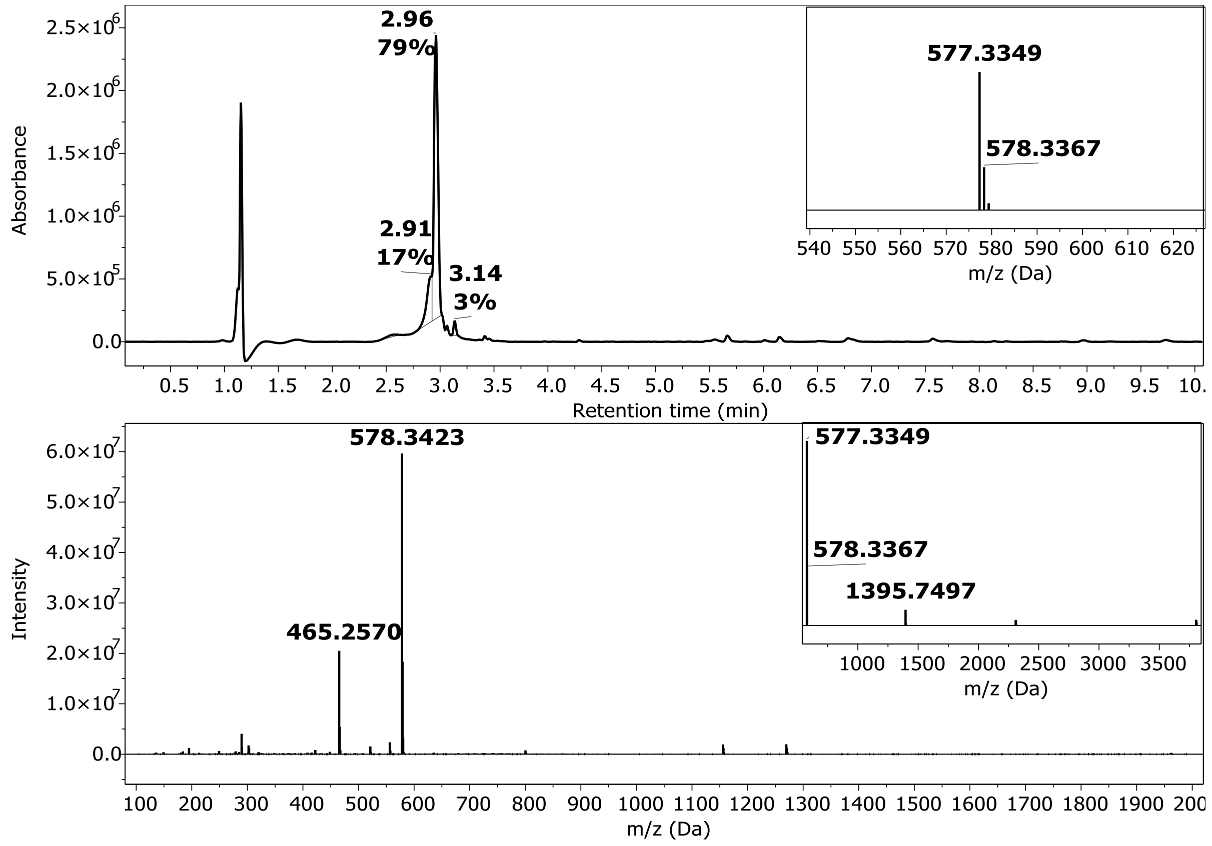

SI Figure 1: LCMS Profile of crude LYRAG. Absorbance chromatogram (λ = 214 nm) of LYRAG; Rt 2.96 min, 79% purity. ESI-TOF spectrum found within Rt 2–8 min (insert: deconvoluted masses). Monoisotopic mass (ESI+) calcd. for C26H43N9O6577.3336, found 577.3349. LCMS Gradient A.

4 Synthesis of DEA- and DIPA-functionalized resins

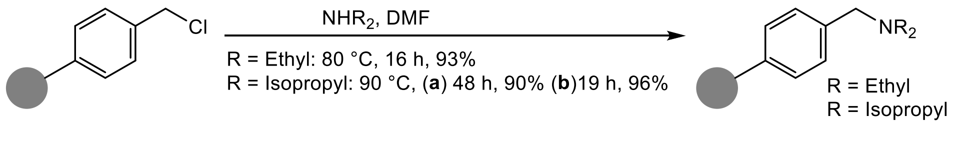

4.1 Diethylamine-functionalized immobilized base (DEA)

Synthesis was adapted from Eseteve et al.[2].

DMF (3.20 mL) was added to Merrifield resin (1.08 g, 3.00 mmol) and diethylamine (2.80 mL, 27.1 mmol, 9.03 equiv.). The mixture was heated to 80 °C and stirred for 16 h. The product was washed three times with 10 mL of DMF, methanol and DCM, respectively and was dried *in vacuo* to yield the desired product (1.10 g, 2.77 mmol) in a 93% yield.

Characterization: Absence of C–Cl band (1264 cm^{-1}) in the FT-IR spectrum; the full absence of C–Cl bonds was confirmed by a negative NBP test.[2]

Light yellow powder IR: 1966*w*, 1601*w*, 1508*m*, 1492*m*, 1450*s*, 1420*m*, 1369*s*, 1290*m*, 1198*m*, 1165*m*, 1057*m*, 1019*w*.

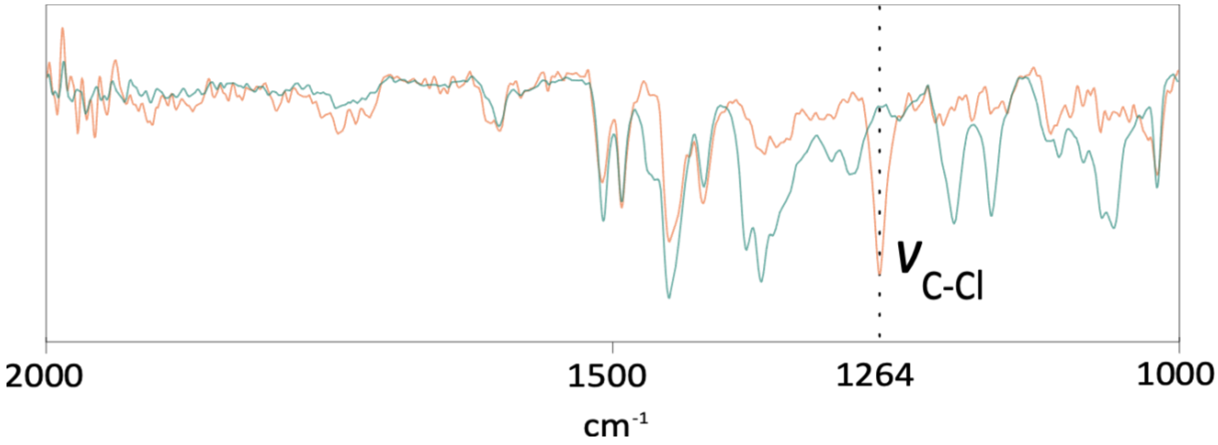

SI Figure 2: Partial IR spectra for the starting material Merrifield resin (3.5 – 4.5 mmol Cl/g nominal loading, orange spectrum) and DEA (green spectrum). Disappearance of the C–Cl characteristic band is highlighted by the dotted line.

4.2 Diisopropylamine-functionalized immobilized base batch 1 (DIPA b.1)

DMF (4.00 mL) was added to Merrifield resin (2.58 g, 7.15 mmol) and diisopropylamine (9.50 mL, 67.8 mmol, 9.47 eq.). The mixture was heated to 90 °C and stirred at reflux for 24 h. The product was washed three times with 10 mL of DMF, methanol and DCM, respectively and was dried *in vacuo* to yield the desired product (2.72 g, 6.40 mmol) in a 90% yield.

Characterization: Absence of C–Cl band (1264 cm^{-1}) in the FT-IR spectrum; the full absence of C–Cl bonds was confirmed by a negative NBP test.

Light yellow powder IR: 1603*w*, 1509*m*, 1493*m*, 1451*m*, 1420*m*, 1380*m*, 1361*s*, 1205*m*, 1176*s*, 1114*m*, 1017*w*.

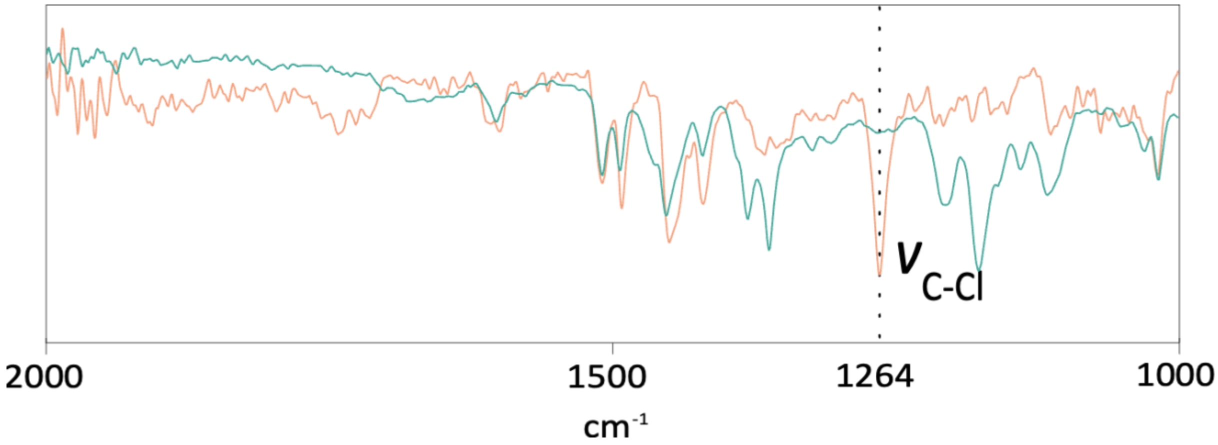

SI Figure 3: Partial IR spectra for the starting material Merrifield resin (3.5 – 4.5 mmol Cl/g nominal loading, orange spectrum) and DIPA b.1 (green spectrum). Disappearance of the C–Cl characteristic band is highlighted by the dotted line.

4.3 Diisopropylamine-functionalized immobilized base batch 2 (DIPA b.2)

DMF (7.00 mL) was added to Merrifield resin (3.53 g, 9.77 mmol) and diisopropylamine (12.0 mL, 85.6 mmol, 8.76 eq.). The mixture was heated to 90 °C and stirred at reflux for 19 h. The product was washed three times with 10 mL of DMF, methanol and DCM, respectively and was dried *in vacuo* to yield the desired product (3.99 g, 9.37 mmol) in a 96% yield.

Characterization: Absence of C–Cl band (1264 cm^{-1}) in the FT-IR spectrum; the full absence of C–Cl bonds was confirmed by a negative NBP test.

Kight yellow powder IR: 1982*w*, 1451*m*, 1361*s*, 1207*m*, 1176*s*, 1017*w*.

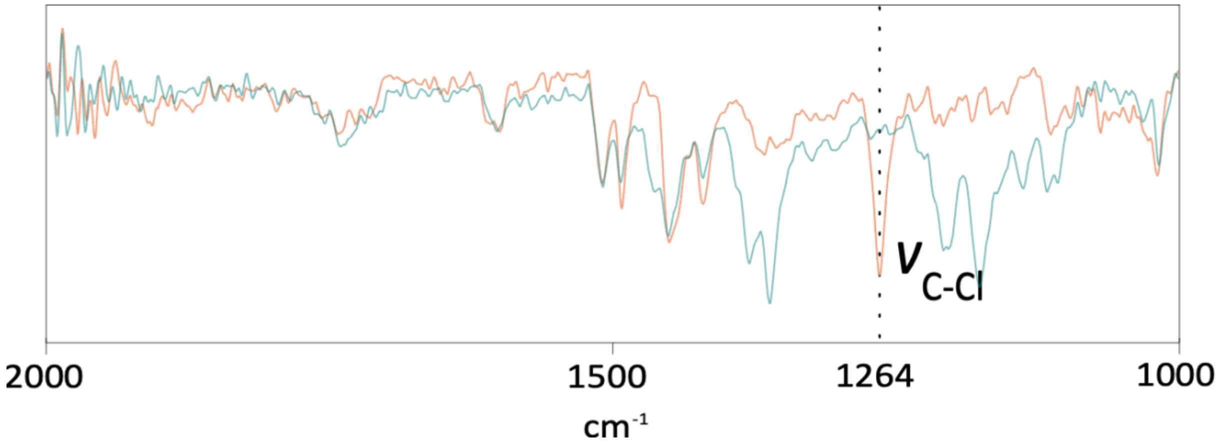

SI Figure 4: Partial IR spectra for the starting material Merrifield resin (3.5 – 4.5 mmol Cl/g nominal loading, orange spectrum) and DIPA b.2 (green spectrum). Disappearance of the C–Cl characteristic band is highlighted by the dotted line.

4.4 IV NBP test

To 10 mg of resin, 0.2 ml of NBP 0.05M in DCM/DMF (1:1, *v*/*v*) with 5 % triethylamine (TEA) were added. Purple color is positive result, indicating the presence of the chlorine group, while the colorless resins show the lack of it. The developed color was allowed to stabilize.

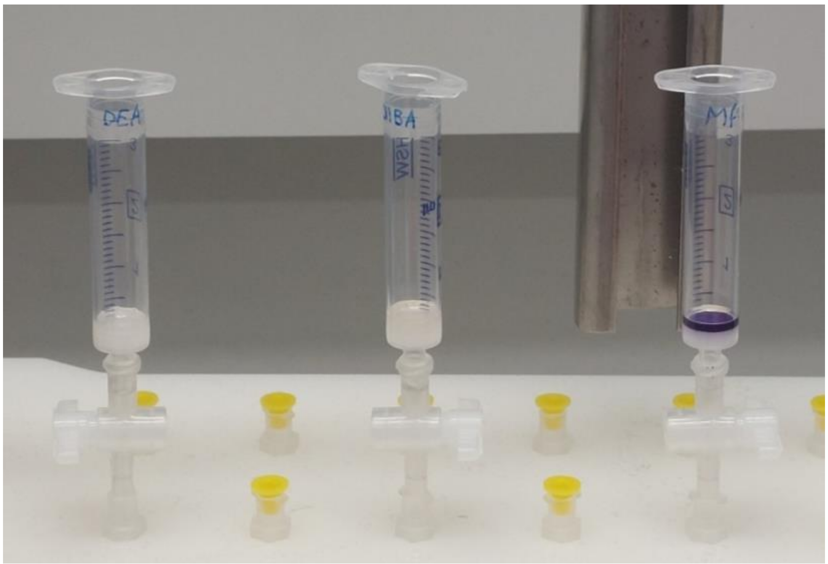

SI Figure 5: Picture of the NBP test carried out for the supported bases, left is DEA immobilized base, middle is DIPA immobilized base, right is Merrifield's resin

4.5 Basicity test with bromophenol blue

Bromophenol blue was added to ethanol. Of the resulting solution, 200 µL were added to MS vials containing 20 mg of the corresponding immobilized bases.

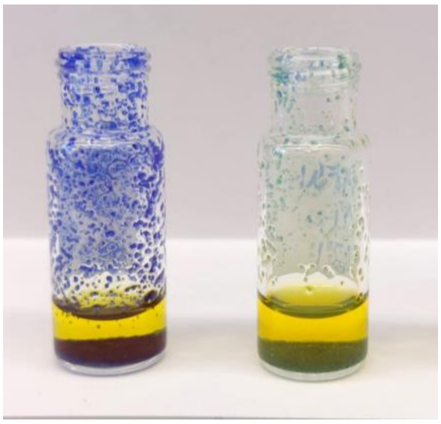

SI Figure 6: From left to right: DEA immobilized base (DEA), DIPA immobilized base.

5 Experimental setup

5.1 Flow setup

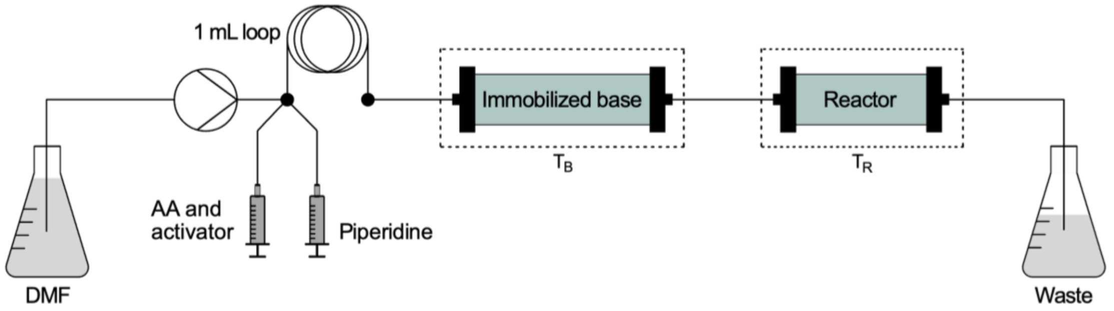

SI Figure 7: The flow setup with a Teledyne isco Reaxus LD Class HPLC pump with a 12 mL/min stainless steel pump head. Tubes: 1 mL loop: PEEK; tube1: stainless steel, l1 = 470 mm, id1 = 0.762 mm, V1 = 0.21 mL; tube2: PEEK, l2 = 400 mm, id2 = 0.762 mm, V2 = 0.18 mL. The vessels were heated using water or oil baths.

5.2 Reactor

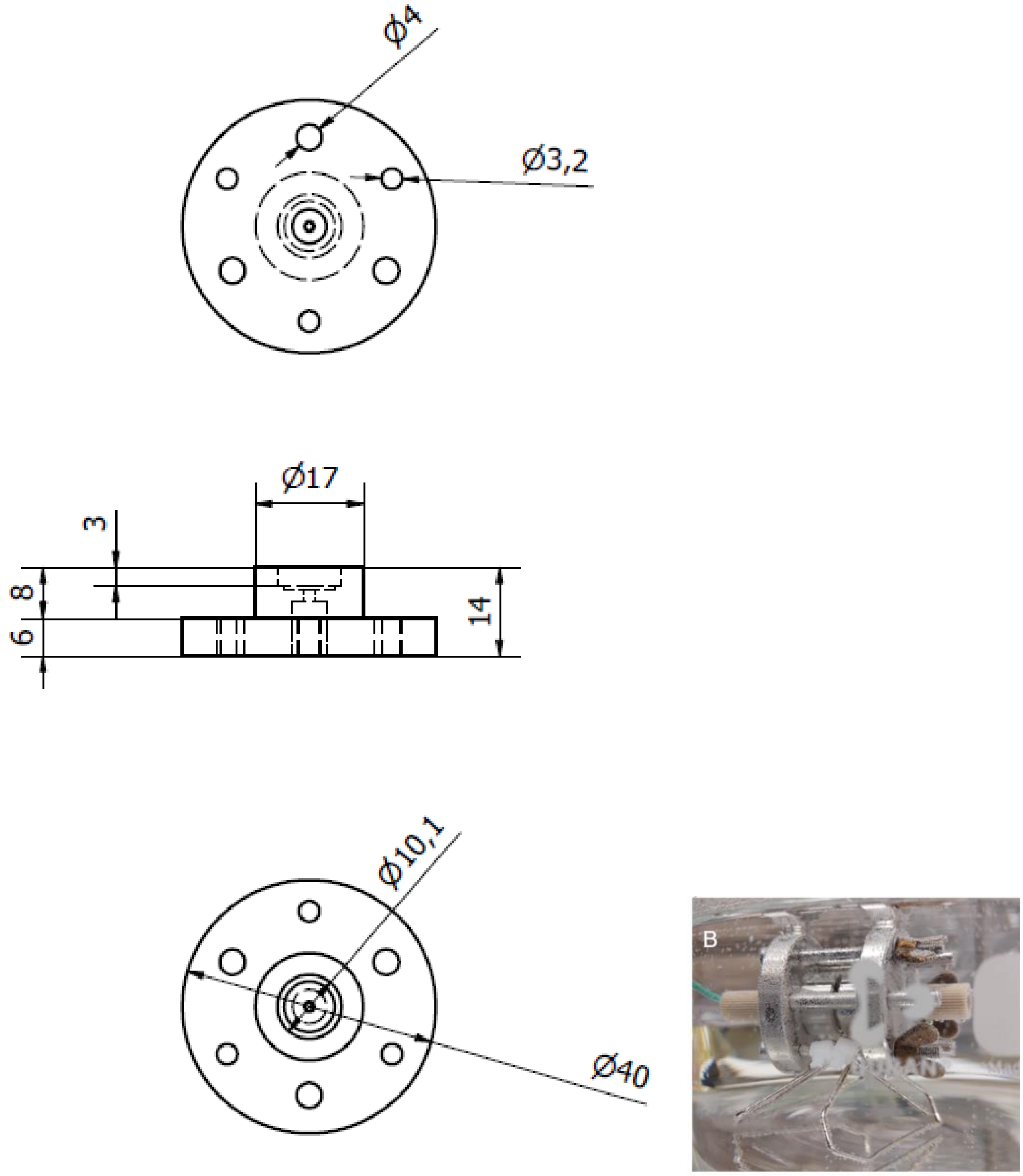

SI Figure 8: Schematic representation and photo of the reactor.

6 Immobilized base-SPPS

Unless stated otherwise, Rink amide resin (0.41 mmol/g loading) was filled into the reactor and swelled with DMF. The given base column was subjugated to a fast washing and deprotection step (HPLC pump at 10 mL/min, starting with 30 s DMF, 30 s 20% piperidine in DMF solution and finally 1 min of DMF), then the setup was connected. The system was flushed with DMF before submerging both base column and reactor in water baths of the given temperature. Unless otherwise stated, the amino acids were dissolved in 0.5 mL, 0.38 M activator solution and were added quickly in the appropriate order at the given flow rate

to the preheated setup. The system was washed at the given flow rate and for the given time with DMF (slow washing step, t_{sw} in Tables), followed by a fast washing and deprotection step (t_{fw} in Tables) by HPLC pump at 10 mL/min, starting with 30 s DMF, 30 s 20% piperidine and 1% formic acid in DMF solution and finally 1 min of DMF. Once all amino acids were added, the reactor was disconnected from the system, and the resin was washed with DCM (3 × 5 mL) and dried under reduced pressure.

7 Comparison of DEA- and DIPA-functionalized resins

7.1 Coupling of ALF to the LYRAG-coupled resin

7.1.1 DEA-functionalized resin

The sequence ALF was synthesized on a previously synthesized LYRAG-tag (LYRAG TAG 5, 9.1 mg, 3.7 μmol) using the immobilized base-SPPS standard protocol (Section SI3). Cleavage of the peptidyl resin according to Cleavage Protocol (Section 1.3) afforded the crude peptide (1.6 mg, 100% purity by LCMS [**SI Figure 9**], 96% purity by UHPLC [SI Figure 10]).

AA	m(AA) [mg]	Immob. base	Flow rate [mL/min]	Base temp. [°C]	Reactor temp. [°C]	Activator	t_{sw} [min]	t_{fw} [min]
A	61.9	DEA 1	0.6	90	90	HATU	4	2
L	71.5	DEA 1	0.6	90	90	HATU	4	2
F	77.1	DEA 1	0.6	90	90	HATU	4	2

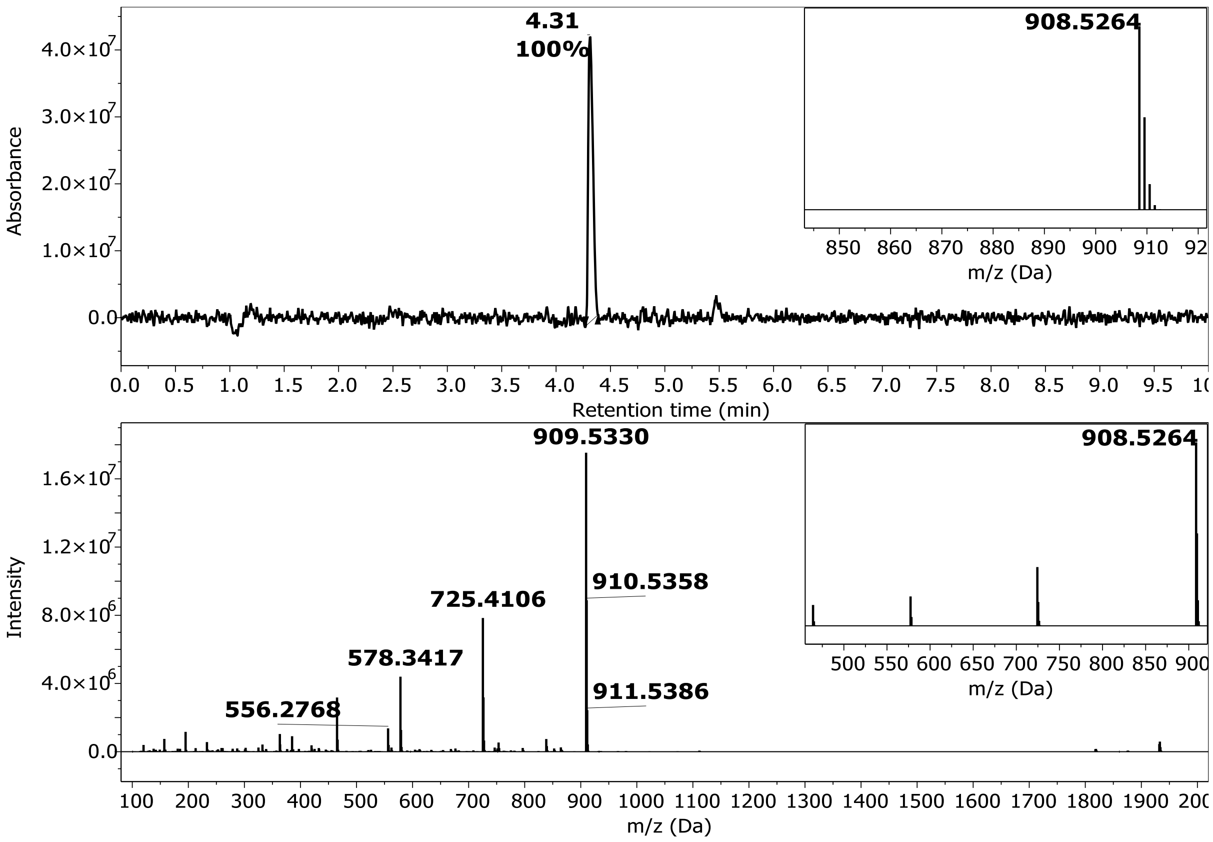

SI Figure 9. *LCMS Profile of crude ALFLYRAG. Absorbance chromatogram (λ = 214 nm) of ALFLYRAG; R_t 4.31 min, 100% purity. ESI-TOF spectrum found within R_t 2–8 min (insert: deconvoluted masses). Monoisotopic mass (ESI+) calcd. for $C_{44}H_{68}N_{12}O_9$ 908.5232, found 908.5264. LCMS Gradient A.*

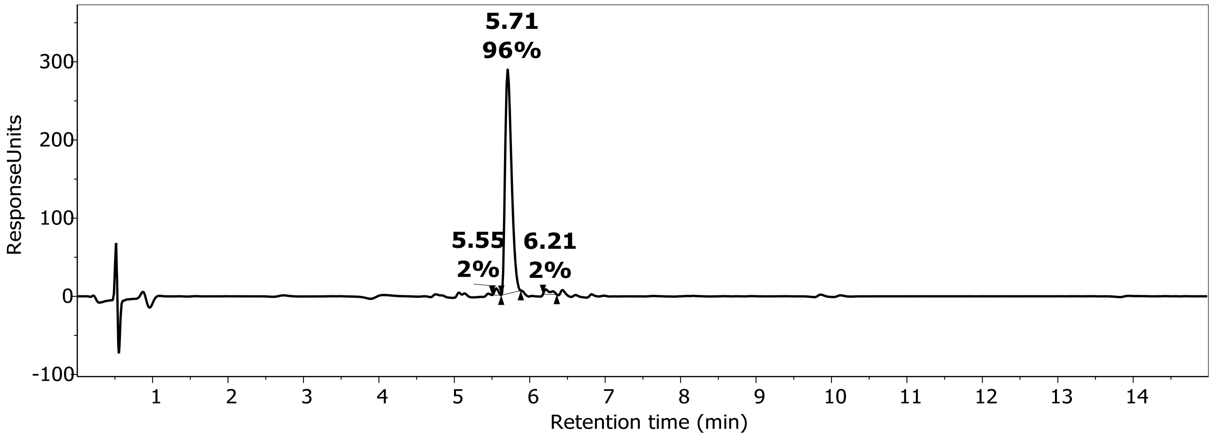

SI Figure 10: UHPLC Profile of crude ALFLYRAG. Absorbance chromatogram (λ = 214 nm) of ALFLYRAG; R_t 5.71 min, 96% purity.

7.1.2 DIPA-functionalized resin

ALF was synthesized on a previously synthesized LYRAG-tag (LYRAG TAG 4, 11.5 mg, 4.7 μmol) using the immobilized base-SPPS standard protocol (Section SI3). Cleavage of the peptidyl resin according to Cleavage Protocol (Section 1.3) afforded the crude peptide (100% purity by LCMS [SI Figure 11] and 99% by UHPLC [SI Figure 12]).

AA	m(AA) [mg]	Immob. base	Flow rate [mL/min]	Base temp. [°C]	Reactor temp. [°C]	Activator	t_{sw} [min]	t_{fw} [min]
A	62.8	DIPA 7	0.6	90	90	HATU	4	2
L	71.6	DIPA 7	0.6	90	90	HATU	4	2
F	77.1	DIPA 7	0.6	90	90	HATU	4	2

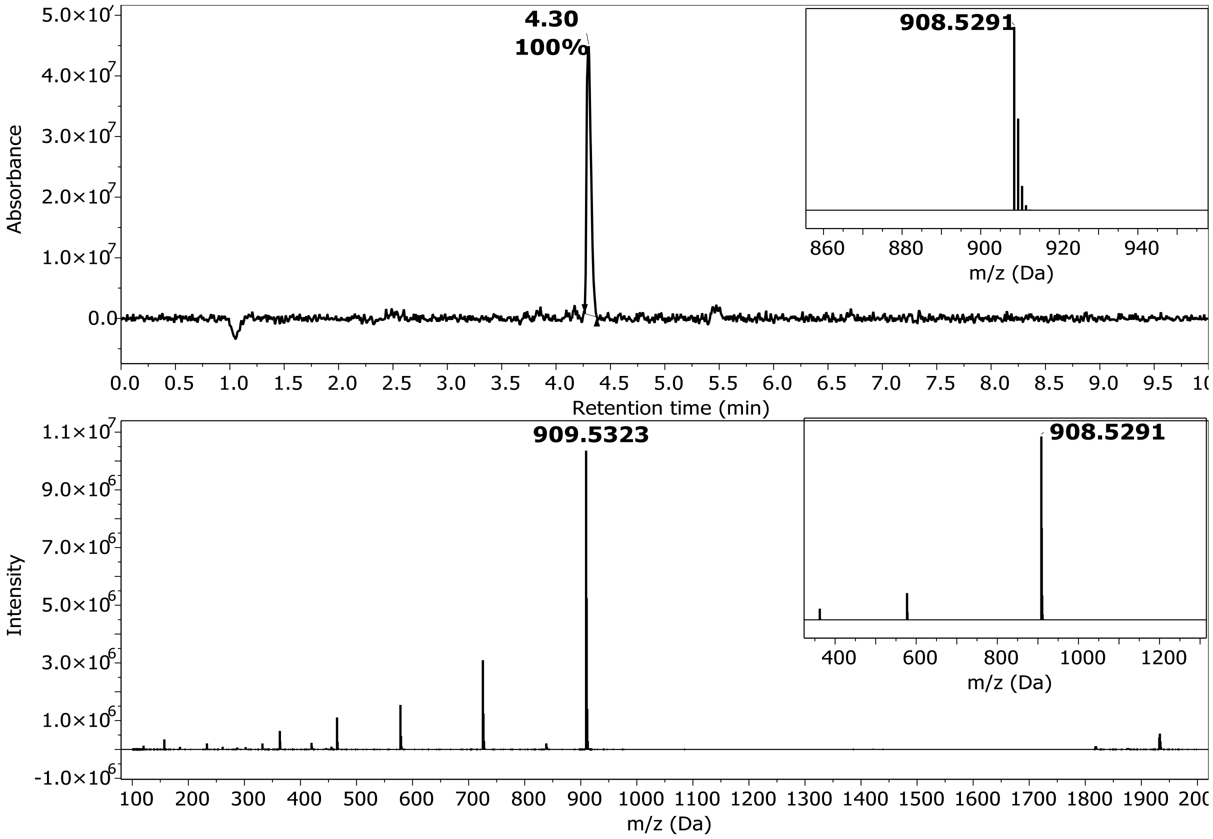

SI Figure 11: LCMS Profile of crude ALFLYRAG. Absorbance chromatogram (λ = 214 nm) of ALFLYRAG; R_t 4.30 min, 100% purity. ESI-TOF spectrum found within R_t 2–8 min (insert: deconvoluted masses). Monoisotopic mass (ESI+) calcd. for $C_{44}H_{68}N_{12}O_9$ 908.5232, found 908.5291. LCMS Gradient A.

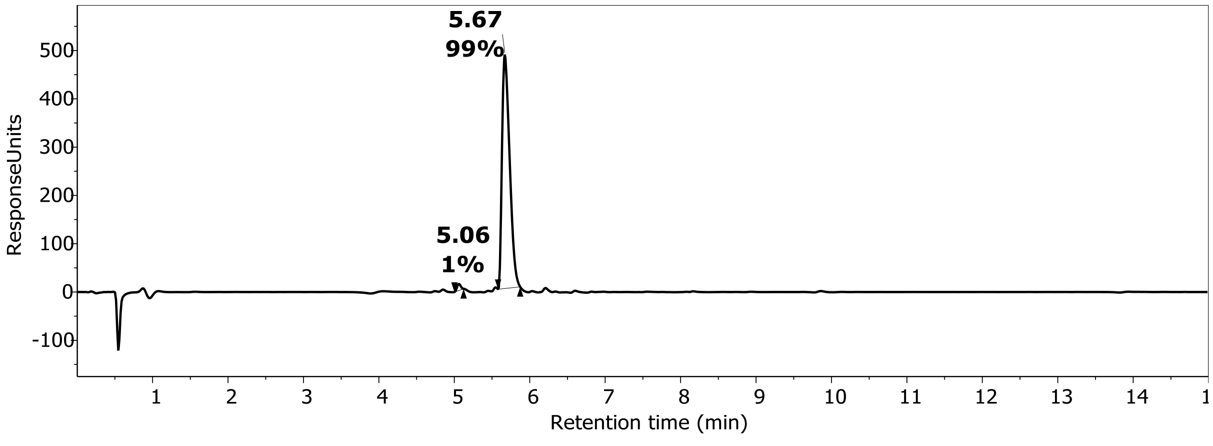

SI Figure 12: UHPLC Profile of crude ALFLYRAG. Absorbance chromatogram (λ = 214 nm) of ALFLYRAG; R_t 5.67 min, 99% purity.

7.2 Coupling of GCF to the LYRAG coupled resin

In all cases L:D ration was calculated the following way: peak area of products containing the L epimer divided by the peak area of the products containing the D epimer.

7.2.1 Batch synthesis of D and L GCFs

GCF-LYRAG

The sequence GCF (synthesized with L-Cys) was synthesized on a previously synthesized LYRAG-tag (LYRAG-TAG 1, 9.2 mg, 3.8 μmol) using the batch SPPS standard protocol (Section 1.4) at room temperature. Cleavage of the peptidyl resin according to Cleavage Protocol (Section 1.3) afforded the crude peptide (96% purity by LCMS [SI Figure 13], 90% purity by UHPLC [SI Figure 14]).

AA	m(AA) [mg]	DIPEA	HATU
G	60.2	50 μL	0.5 mL
C	117.1	50 μL	0.5 mL
F	77.6	50 μL	0.5 mL
L:D	89.4:1		

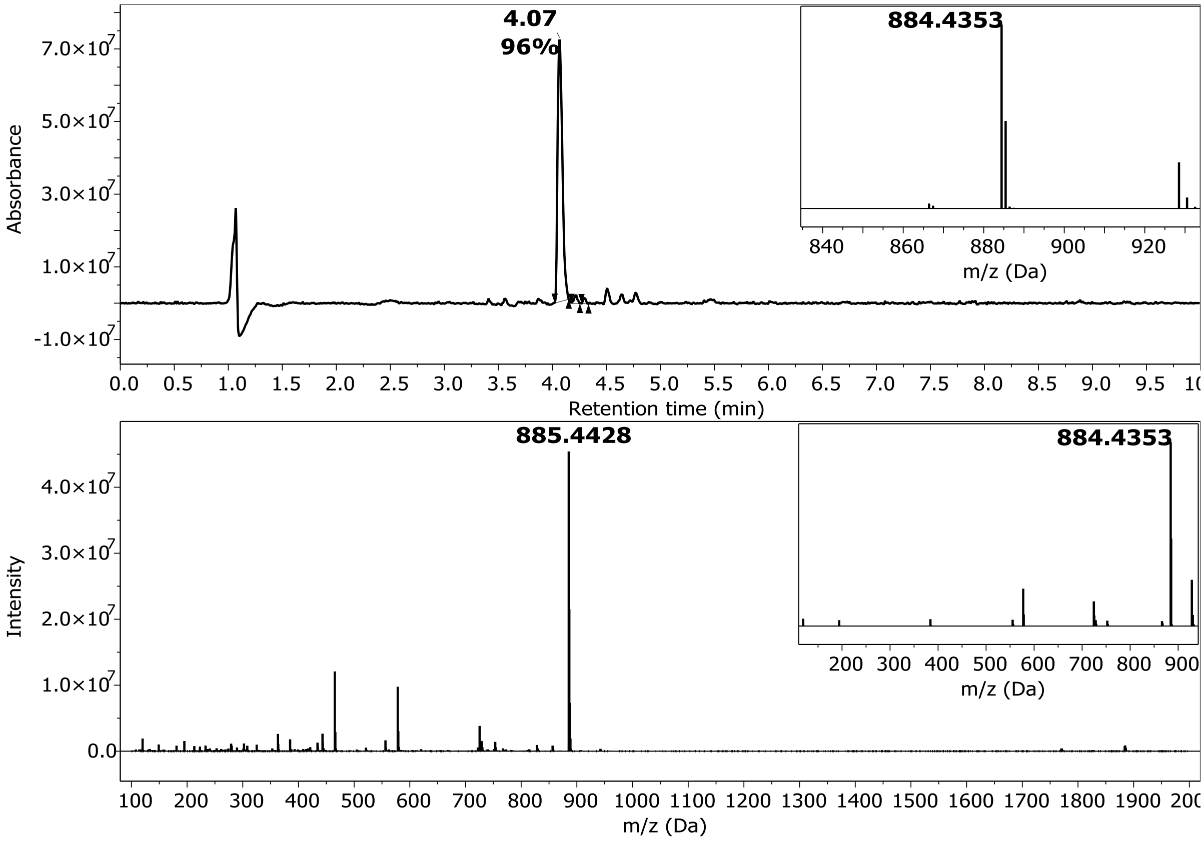

SI Figure 13: LCMS Profile of crude GCFLYRAG. Absorbance chromatogram (λ = 214 nm) of GCFLYRAG; R_t 4.07 min, 96% purity. ESI-TOF spectrum found within R_t 2–8 min (insert: deconvoluted masses). Monoisotopic mass (ESI+) calcd. for $C_{40}H_{60}N_{12}O_9S$ 884.4327, found 884.4353. LCMS Gradient A.

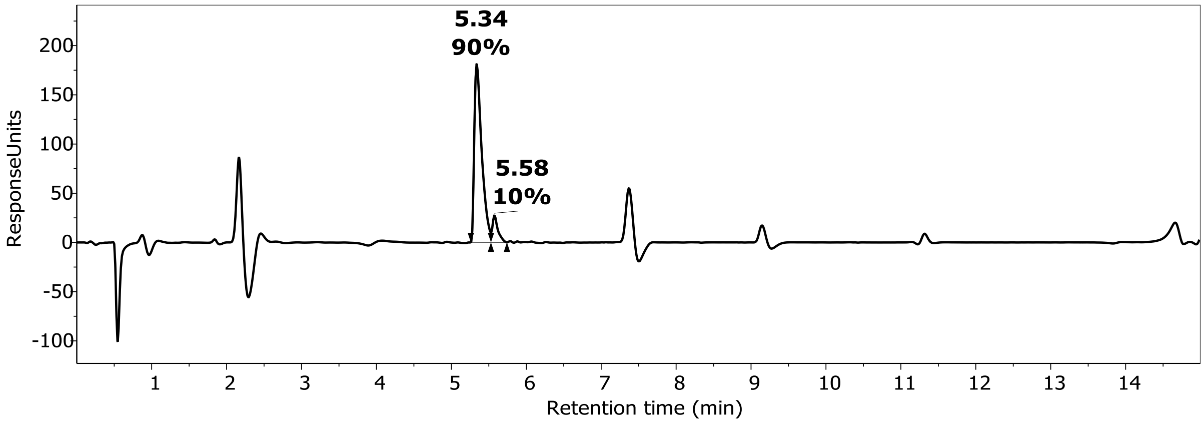

SI Figure 14: UHPLC Profile of crude GCFLYRAG. Absorbance chromatogram (λ = 214 nm) of GcFLYRAG; R_t 5.34 min, 90% purity.[1]

GcF-LYRAG

The sequence GcF (synthesized with D-Cys) was synthesized on a previously synthesized LYRAG-tag (LYRAG-TAG 1, 10.4 mg, 4.3 µmol) using the batch SPPS standard protocol (Section 1.4) at room temperature. Cleavage of the peptidyl resin according to Cleavage Protocol (Section 1.3) afforded the crude peptide (85% purity by LCMS [SI Figure 15], 84% purity by UHPLC [SI Figure 16]).

AA	m(AA) [mg]	DIPEA	HATU
G	59.5	50 µL	0.5 mL
c	117	50 µL	0.5 mL
F	77.1	50 µL	0.5 mL
L:D	-		

[1] Additional peaks are visible in this spectrum due to air bubbles in the machine.

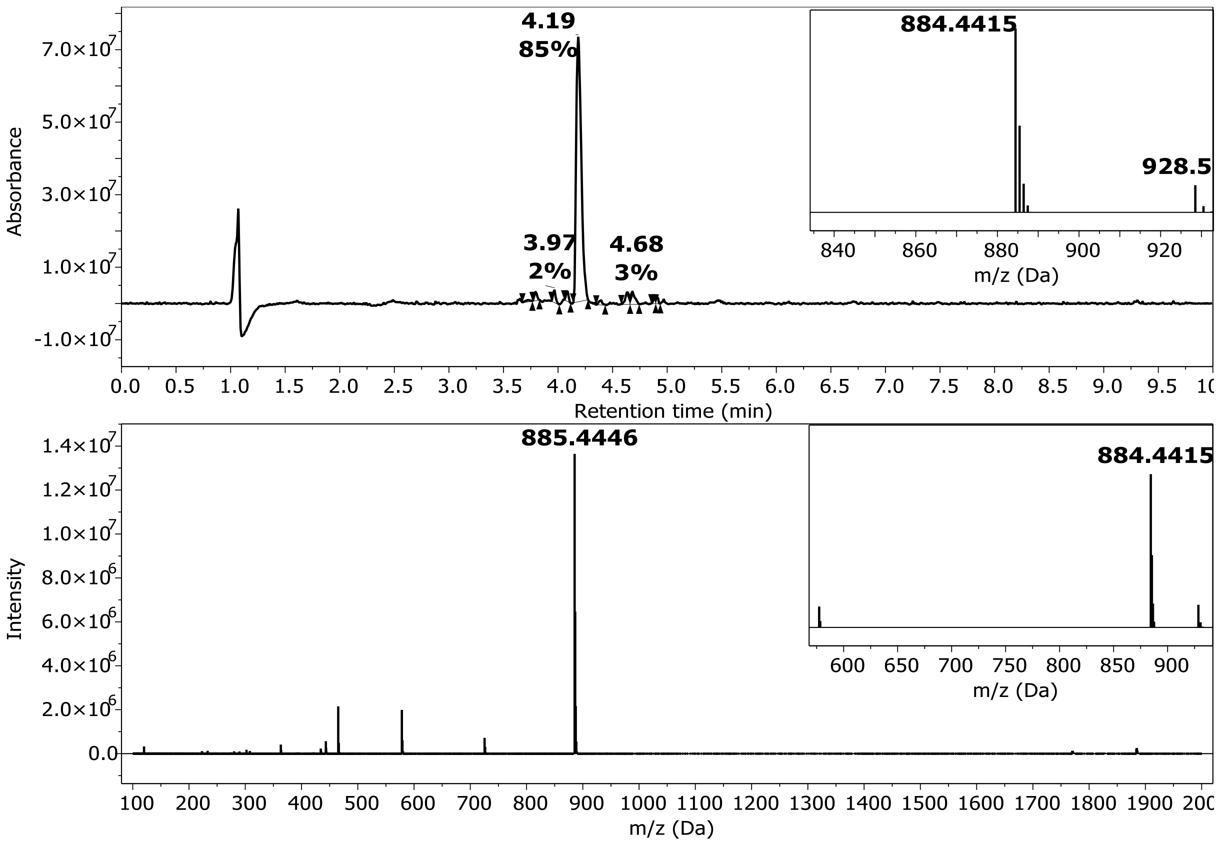

SI Figure 15: LCMS Profile of crude GcFLYRAG. Absorbance chromatogram (λ = 214 nm) of GcFLYRAG; R_t 4.19 min, 85% purity. ESI-TOF spectrum found within R_t 2–8 min (insert: deconvoluted masses). Monoisotopic mass (ESI+) calcd. for $C_{40}H_{60}N_{12}O_9S$ 884.4327, found 884.4415. LCMS Gradient A.

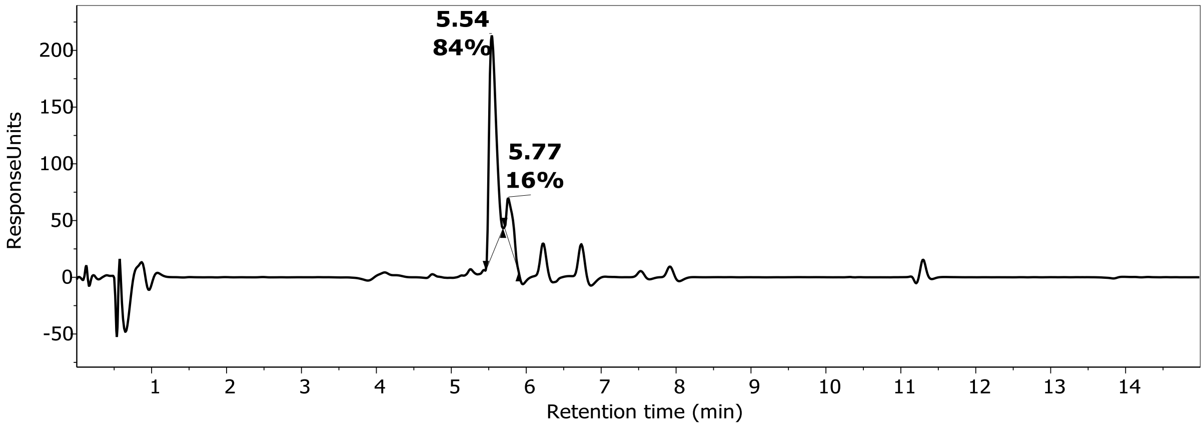

SI Figure 16: UHPLC Profile of crude GcFLYRAG. Absorbance chromatogram (λ = 214 nm) of GcFLYRAG; R_t 5.54 min, 84% purity.[2]

7.2.2 DEA-functionalized resin

The sequence GCF was synthesized on a previously synthesized LYRAG-tag (LYRAG TAG 5, 9.5 mg, 3.9 µmol) using the immobilized base-SPPS standard protocol (Section SI3). Cleavage of the peptidyl resin

[2] Additional peaks are visible in this spectrum due to air bubbles in the machine.

according to Cleavage Protocol (Section 1.3) afforded the crude peptide (74% purity by LCMS [SI Figure 17]).

AA	m(AA) [mg]	Immob. base	Flow rate [mL/min]	Base temp. [°C]	Reactor temp. [°C]	Activator	t_{sw} [min]	t_{fw} [min]
G	58.8	DEA 1	0.6	90	90	HATU	4	2
C	118.3	DEA 1	0.6	90	90	HATU	4	2
F	76.9	DEA 1	0.6	90	90	HATU	4	2
L:D	3.3:1							

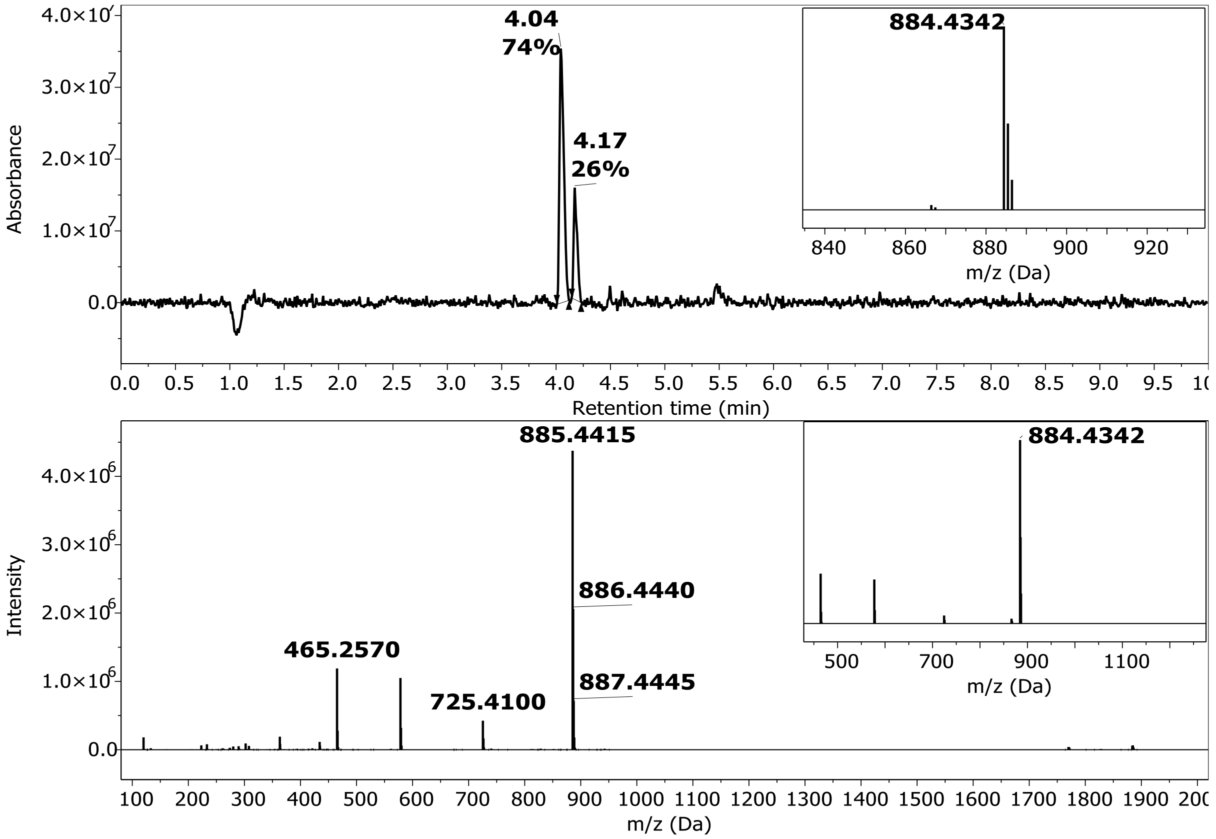

SI Figure 17: LCMS Profile of crude GCFLYRAG. Absorbance chromatogram (λ = 214 nm) of GCFLYRAG; R_t 4.04 min, 71% purity. ESI-TOF spectrum found within R_t 2–8 min (insert: deconvoluted masses). Monoisotopic mass (ESI+) calcd. for $C_{40}H_{60}N_{12}O_9S$ 884.4327, found 884.4342. LCMS Gradient A.

7.2.3 DIPA-functionalized resin

The sequence GCF was synthesized on a previously synthesized LYRAG-tag (LYRAG TAG 2, 9.80 mg, 4.0 μmol) using the immobilized base-SPPS standard protocol (Section SI3). Cleavage of the peptidyl resin according to Cleavage Protocol (Section 1.3) afforded the crude peptide (2.2 mg, 96% purity by LCMS [SI Figure 18]).

AA	m(AA) [mg]	Immob. base	Flow rate [mL/min]	Base temp. [°C]	Reactor temp. [°C]	Activator	t_{sw} [min]	t_{fw} [min]
G	60.3	DIPA 4	0.6	90	90	HATU	4	2
C	116.6	DIPA 4	0.6	90	90	HATU	4	2
F	77.1	DIPA 4	0.6	90	90	HATU	4	2
L:D	50.1:1							

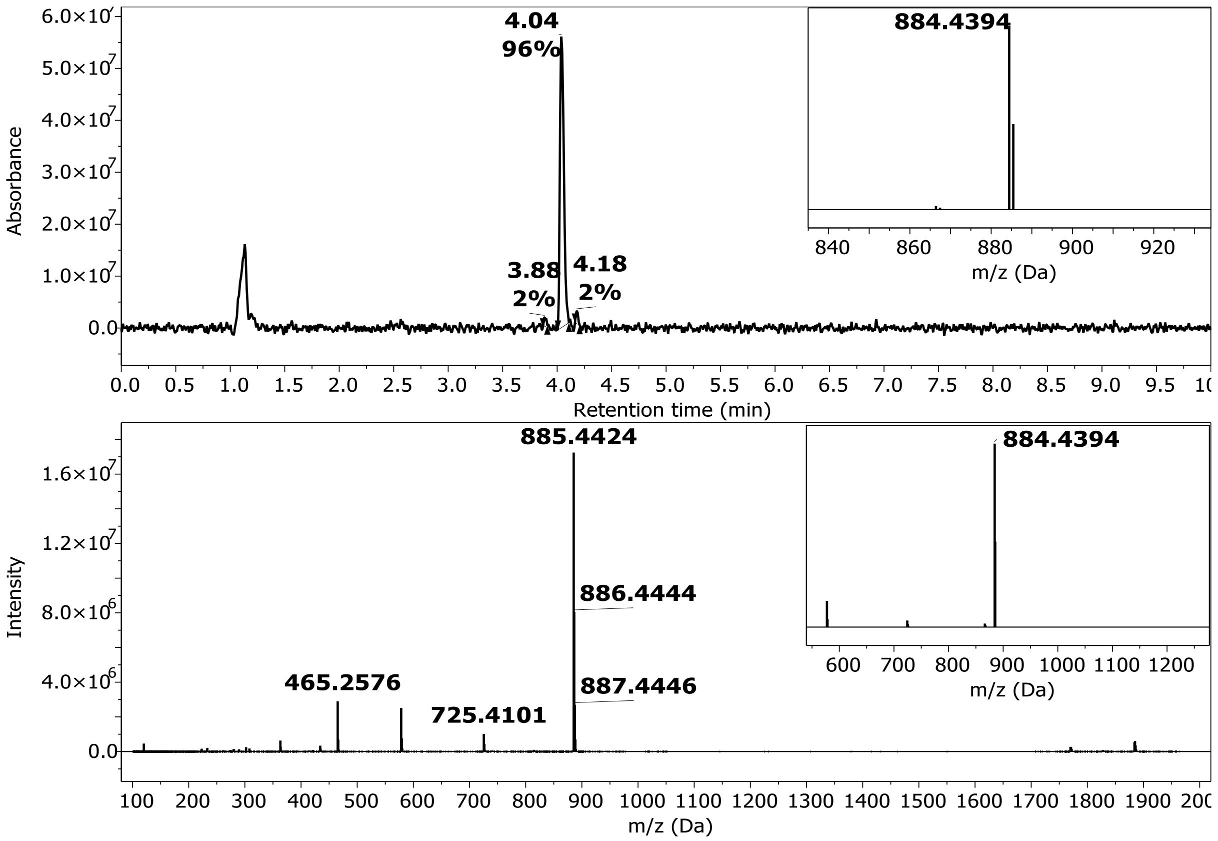

SI Figure 18: LCMS Profile of crude GCFLYRAG. Absorbance chromatogram (λ = 214 nm) of GCFLYRAG; R_t 4.04 min, 96% purity. ESI-TOF spectrum found within R_t 2–8 min (insert: deconvoluted masses). Monoisotopic mass (ESI+) calcd. for $C_{40}H_{60}N_{12}O_9S$ 884.4327, found 884.4394. LCMS Gradient A.

7.3 Coupling of FHL to the LYRAG coupled resin

7.3.1 Batch synthesis of D and L FHLs

FHL-LYRAG

The sequence FHL was synthesized on a previously synthesized LYRAG-tag (LYRAG-TAG 1, 11.6 mg, 4.8 μmol) using the batch SPPS standard protocol (Section 1.4) at room temperature. Cleavage of the peptidyl resin according to Cleavage Protocol (Section 1.3) afforded the crude peptide (82% purity by LCMS [SI Figure 19]).

AA	m(AA) [mg]	DIPEA	HATU
F	76.3	50 μL	0.5 mL
H	123.7	50 μL	0.5 mL
L	70.9	50 μL	0.5 mL
L:D	8.2:1		

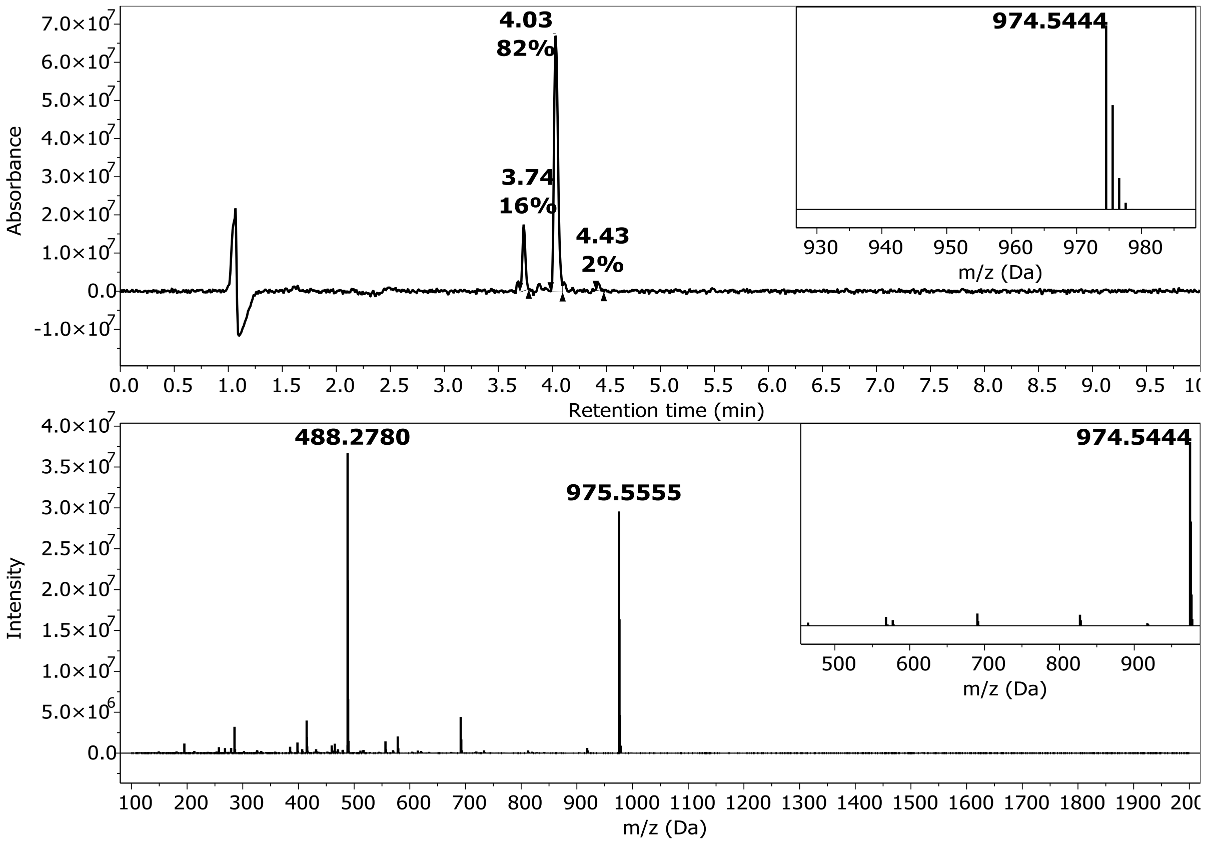

SI Figure 19: LCMS Profile of crude FHLLYRAG. Absorbance chromatogram (λ = 214 nm) of FHLLYRAG; R_t 4.03 min, 82% purity. ESI-TOF spectrum found within R_t 2–8 min (insert: deconvoluted masses). Monoisotopic mass (ESI+) calcd. for $C_{47}H_{70}N_{14}O_9$ 974.5450, found 974.5444. LCMS Gradient A.

FhL-LYRAG

The sequence FhL (synthesized with D-His) was synthesized on a previously synthesized LYRAG-tag (LYRAG-TAG 1, 11.1 mg, 4.6 μmol) using the batch SPPS standard protocol (Section 1.4) at room temperature. Cleavage of the peptidyl resin according to Cleavage Protocol (Section 1.3) afforded the crude peptide (83% purity by LCMS [SI Figure 20]).

AA	m(AA) [mg]	DIPEA	HATU
F	77.9	50 µL	0.5 mL
h	123.2	50 µL	0.5 mL
L	70.9	50 µL	0.5 mL
L:D	0.1:1		

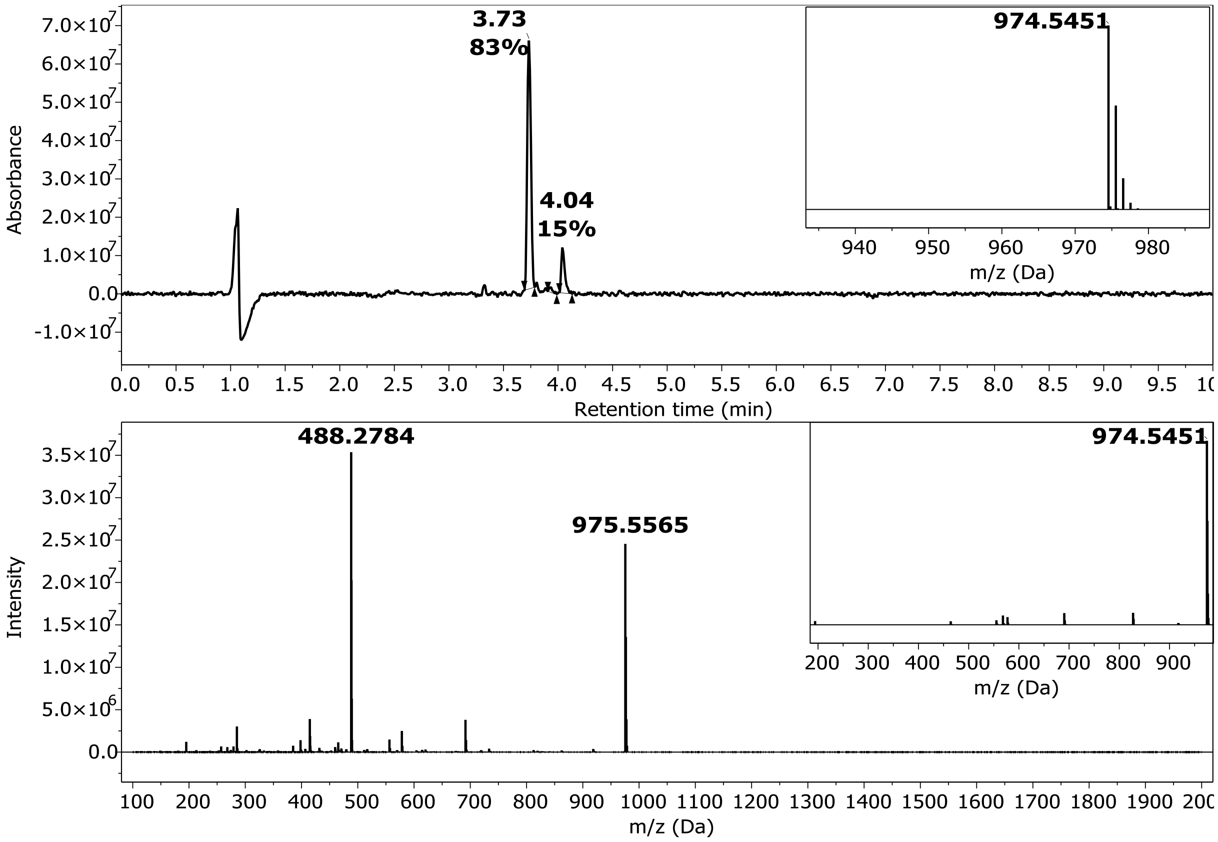

SI Figure 20: LCMS Profile of crude FhLLYRAG. Absorbance chromatogram (λ = 214 nm) of FhLLYRAG; R_t 3.73 min, 83% purity. ESI-TOF spectrum found within R_t 2–8 min (insert: deconvoluted masses). Monoisotopic mass (ESI+) calcd. for $C_{47}H_{70}N_{14}O_9$ 974.5450, found 974.5451. LCMS Gradient A.

7.3.2 DEA-functionalized resin

The sequence FHL was synthesized on a previously synthesized LYRAG-tag (LYRAG TAG 5, 10.1 mg, 4.1 µmol) using the immobilized base-SPPS standard protocol (Section SI3). Cleavage of the peptidyl resin according to Cleavage Protocol (Section 1.3) afforded the crude peptide (68% purity by LCMS [SI Figure 21], 78% BY UHPLC [*SI Figure 22*]).

AA	m(AA) [mg]	Immob. base	Flow rate [mL/min]	Base temp. [°C]	Reactor temp. [°C]	Activator	t_{sw} [min]	t_{fw} [min]

F	78.7	DEA 1	0.6	90	90	HATU	4	2
H	124.7	DEA 1	3.0	23	90	HATU	1	4
L	69.7	DEA 1	0.6	90	90	HATU	4	4
L:D	11.9:1							

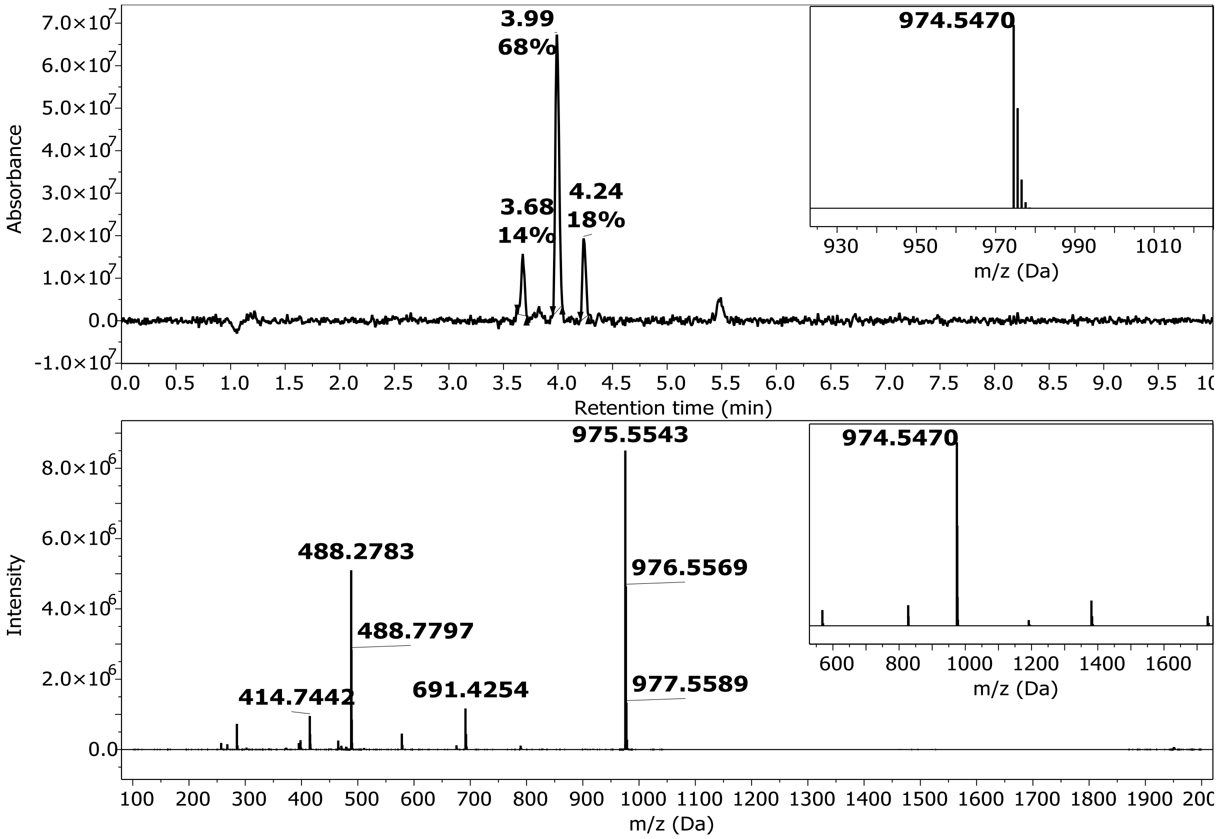

SI Figure 21: LCMS Profile of crude FHLLYRAG. Absorbance chromatogram (λ = 214 nm) of FHLLYRAG; R_t 3.99 min, 68% purity. ESI-TOF spectrum found within R_t 2–8 min (insert: deconvoluted masses). Monoisotopic mass (ESI+) calcd. for $C_{47}H_{70}N_{14}O_9$ 974.5450, found 974.5470. LCMS Gradient A.

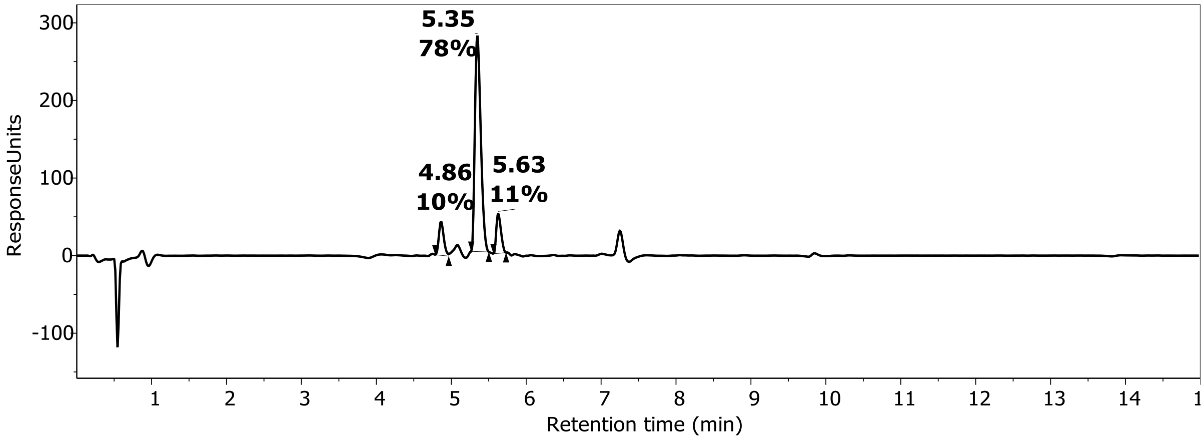

SI Figure 22: UHPLC Profile of crude FHLLYRAG. Absorbance chromatogram (λ = 214 nm) of FHLLYRAG; R_t 5.35 min, 78% purity.

7.3.3 DIPA-functionalized resin

The sequence FHL was synthesized on a previously synthesized LYRAG-tag (LYRAG TAG 5, 9.60 mg, 3.9 μmol) using the immobilized base-SPPS standard protocol (Section SI3). Cleavage of the peptidyl resin according to Cleavage Protocol (Section 1.3) afforded the crude peptide (1.1 mg, 66% purity by LCMS [SI Figure 23], 76% purity by UHPLC [*SI Figure 24*]).

AA	m(AA) [mg]	Immob. base	Flow rate [mL/min]	Base temp. [°C]	Reactor temp. [°C]	Activator	t_{sw} [min]	t_{fw} [min]
F	76.3	DIPA 7	0.6	90	90	HATU	4	2
H	124.0	DIPA 7	3.0	23	90	HATU	1.0	4
L	69.1	DIPA 7	0.6	90	90	HATU	4	4
L:D	17.2:1							

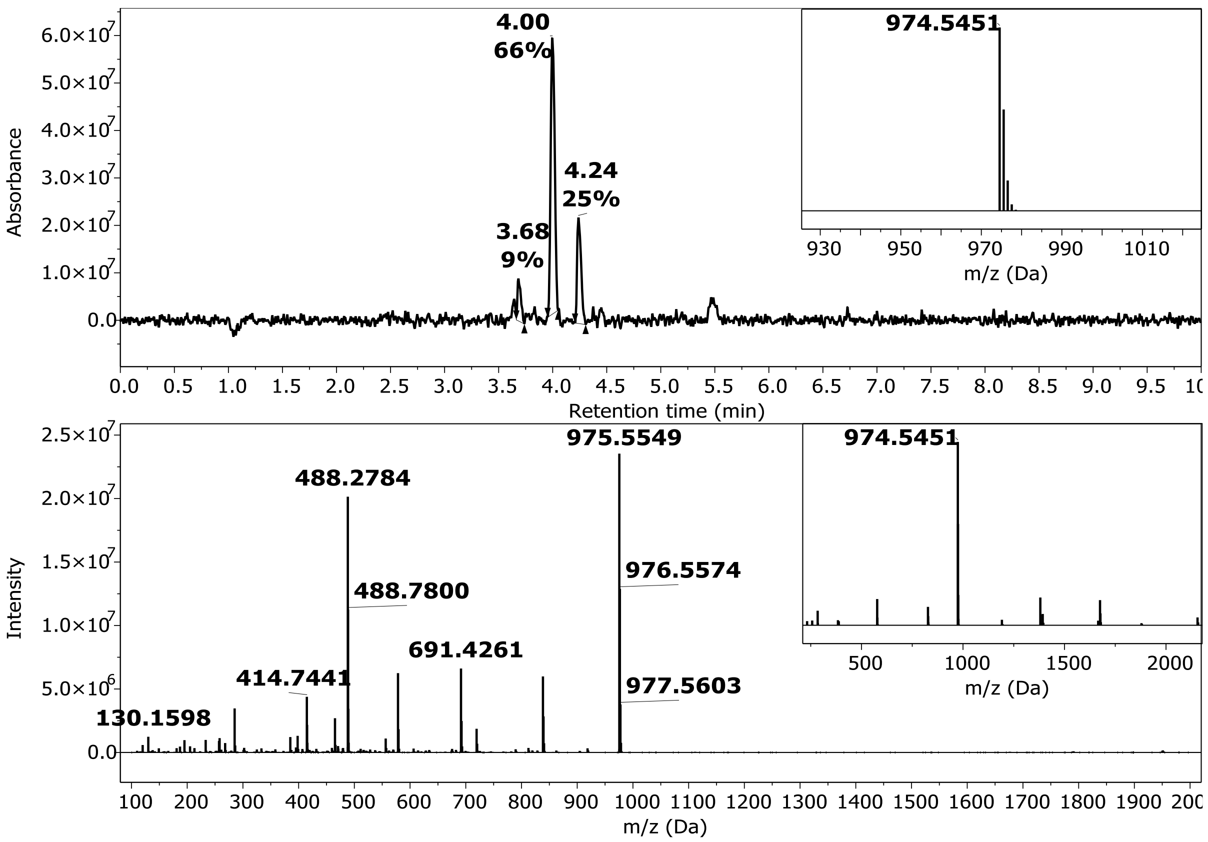

SI Figure 23: LCMS Profile of crude FHLLYRAG. Absorbance chromatogram (λ = 214 nm) of FHLLYRAG; R_t 4.00 min, 74% purity. ESI-TOF spectrum found within R_t 2–8 min (insert: deconvoluted masses). Monoisotopic mass (ESI+) calcd. for $C_{47}H_{70}N_{14}O_9$ 974.5450, found 974.5451. LCMS Gradient A.

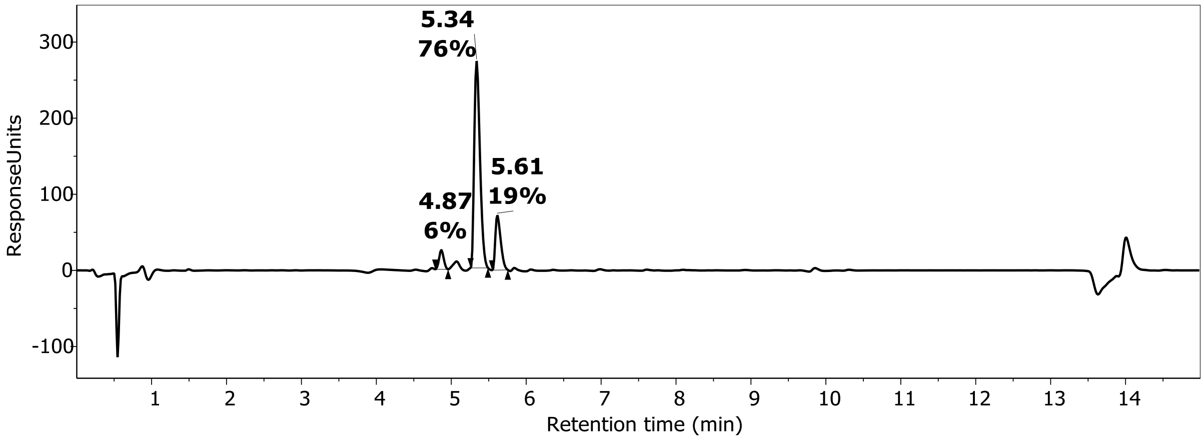

SI Figure 24: UHPLC Profile of crude FHLLYRAG. Absorbance chromatogram (λ = 214 nm) of FHLLYRAG; R_t 5.34 min, 76% purity.

8 Stability investigation of the DIPA-functionalized resin

8.1 Washed with DCM, dried and stored airtight

8.1.1 First experiment, t = 0 days

The sequence ALF was synthesized on a previously synthesized LYRAG-tag (LYRAG TAG 1, 10.7 mg, 4.4 µmol) using the immobilized base-SPPS standard protocol (Section SI3). Cleavage of the peptidyl resin according to Cleavage Protocol (Section 1.3) afforded the crude peptide (89% purity by LCMS [SI Figure 25]).

AA	m(AA) [mg]	Immob. base	Flow rate [mL/min]	Base temp. [°C]	Reactor temp. [°C]	Activator	t_{sw} [min]	t_{fw} [min]
A	62.5	DIPA 1	0.6	90	90	HATU	4	2
L	70.9	DIPA 1	0.6	90	90	HATU	4	2
F	78.0	DIPA 1	0.6	90	90	HATU	4	2

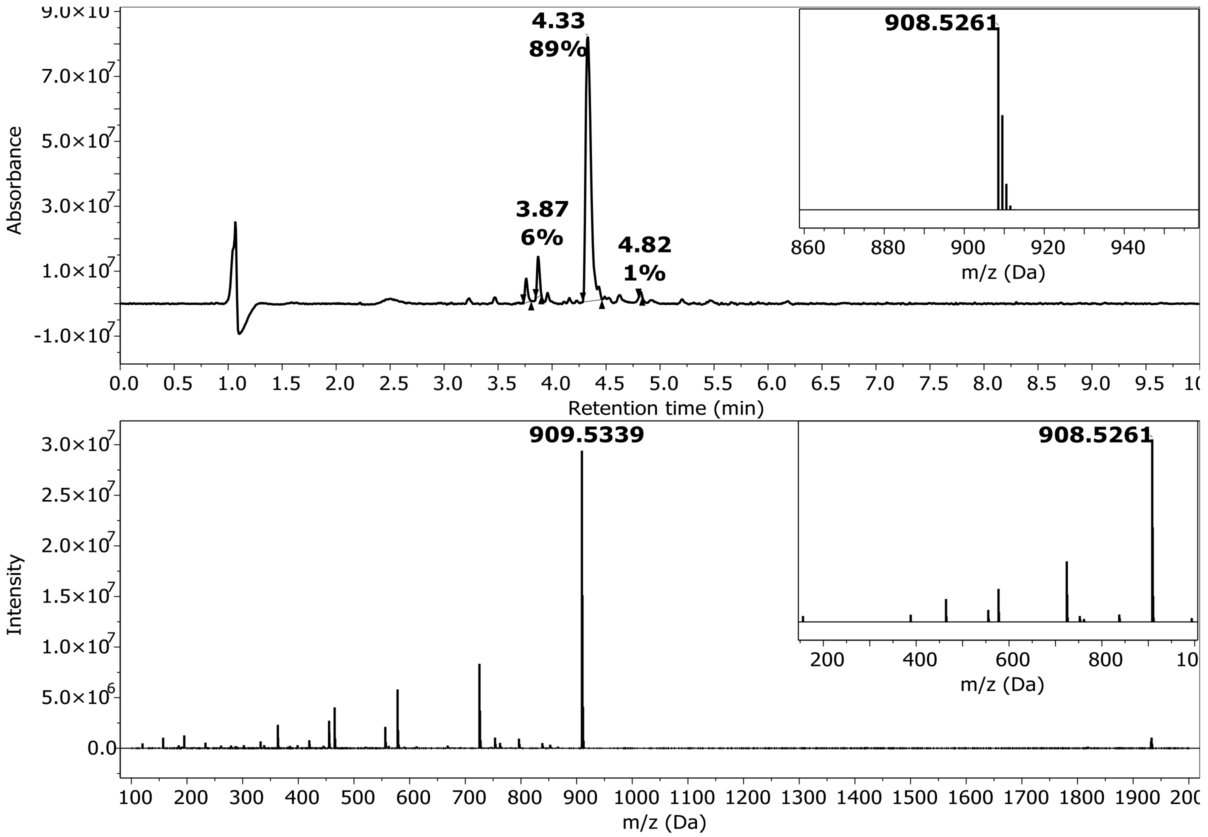

SI Figure 25: LCMS Profile of crude ALFLYRAG. Absorbance chromatogram (λ = 214 nm) of ALFLYRAG; R_t 4.33 min, 89% purity. ESI-TOF spectrum found within R_t 2–8 min (insert: deconvoluted masses). Monoisotopic mass (ESI+) calcd. for $C_{44}H_{68}N_{12}O_9$ 908.5232, found 908.5261. LCMS Gradient A.

8.1.2 Second experiment, t = 7 days

The sequence ALF was synthesized on a previously synthesized LYRAG-tag (LYRAG TAG 1, 10.3 mg, 4.2 µmol) using the immobilized base-SPPS standard protocol (Section SI3). Cleavage of the peptidyl resin according to Cleavage Protocol (Section 1.3) afforded the crude peptide (82% purity by LCMS [SI Figure 26]).

AA	m(AA) [mg]	Immob. base	Flow rate [mL/min]	Base temp. [°C]	Reactor temp. [°C]	Activator	t_{sw} [min]	t_{fw} [min]
A	63.5	DIPA 1	0.6	90	90	HATU	4	2
L	71.1	DIPA 1	0.6	90	90	HATU	4	2
F	77.1	DIPA 1	0.6	90	90	HATU	4	2

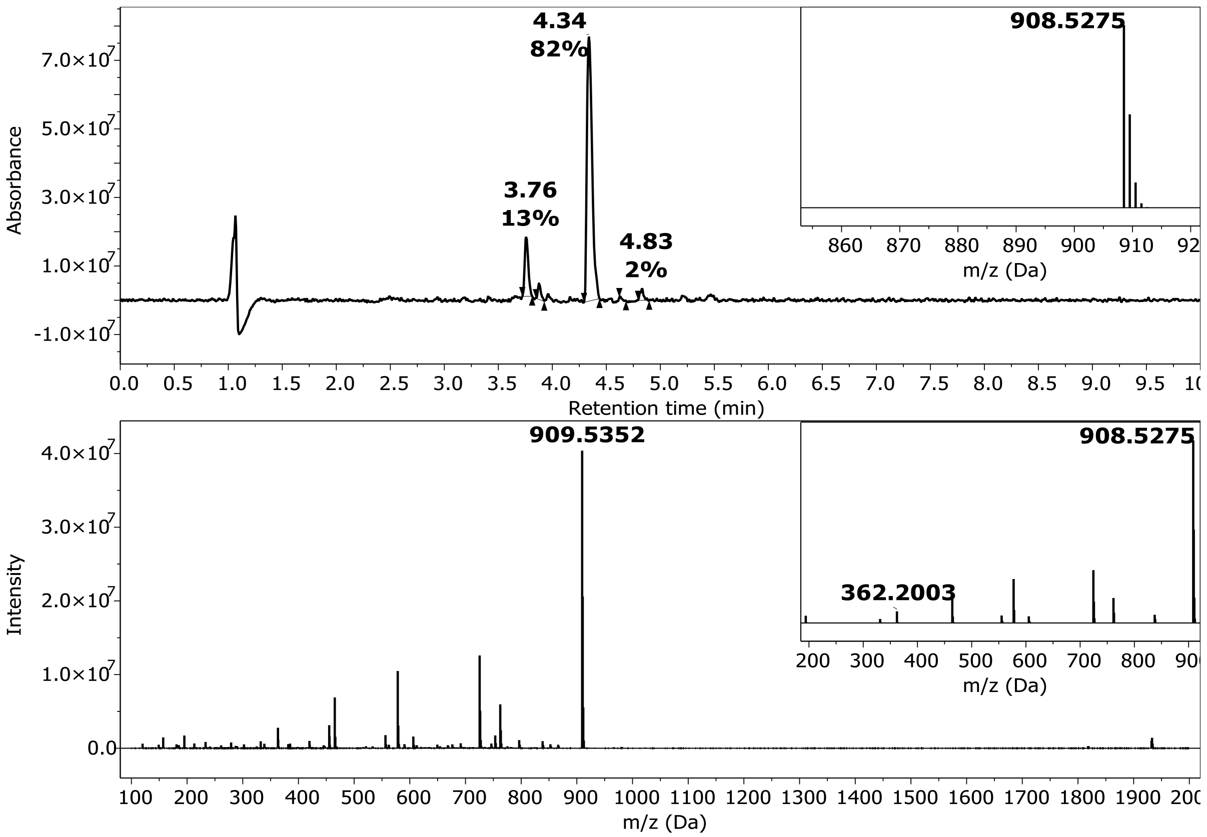

SI Figure 26: LCMS Profile of crude ALFLYRAG. Absorbance chromatogram (λ = 214 nm) of ALFLYRAG; R_t 4.34 min, 82% purity. ESI-TOF spectrum found within R_t 2–8 min (insert: deconvoluted masses). Monoisotopic mass (ESI+) calcd. for $C_{44}H_{68}N_{12}O_9$ 908.5232, found 908.5275. LCMS Gradient A.

8.1.3 Second experiment, t = 21 days

The sequence ALF was synthesized on a previously synthesized LYRAG-tag (LYRAG TAG 1, 9.5 mg, 3.9 μmol) using the immobilized base-SPPS standard protocol (Section SI3). Cleavage of the peptidyl resin according to Cleavage Protocol (Section 1.3) afforded the crude peptide (71% purity by LCMS [SI Figure 27]).

AA	m(AA) [mg]	Immob. base	Flow rate [mL/min]	Base temp. [°C]	Reactor temp. [°C]	Activator	t_{sw} [min]	t_{fw} [min]
A	62.0	DIPA 1	0.6	90	90	HATU	4	2
L	70.5	DIPA 1	0.6	90	90	HATU	4	2
F	77.9	DIPA 1	0.6	90	90	HATU	4	2

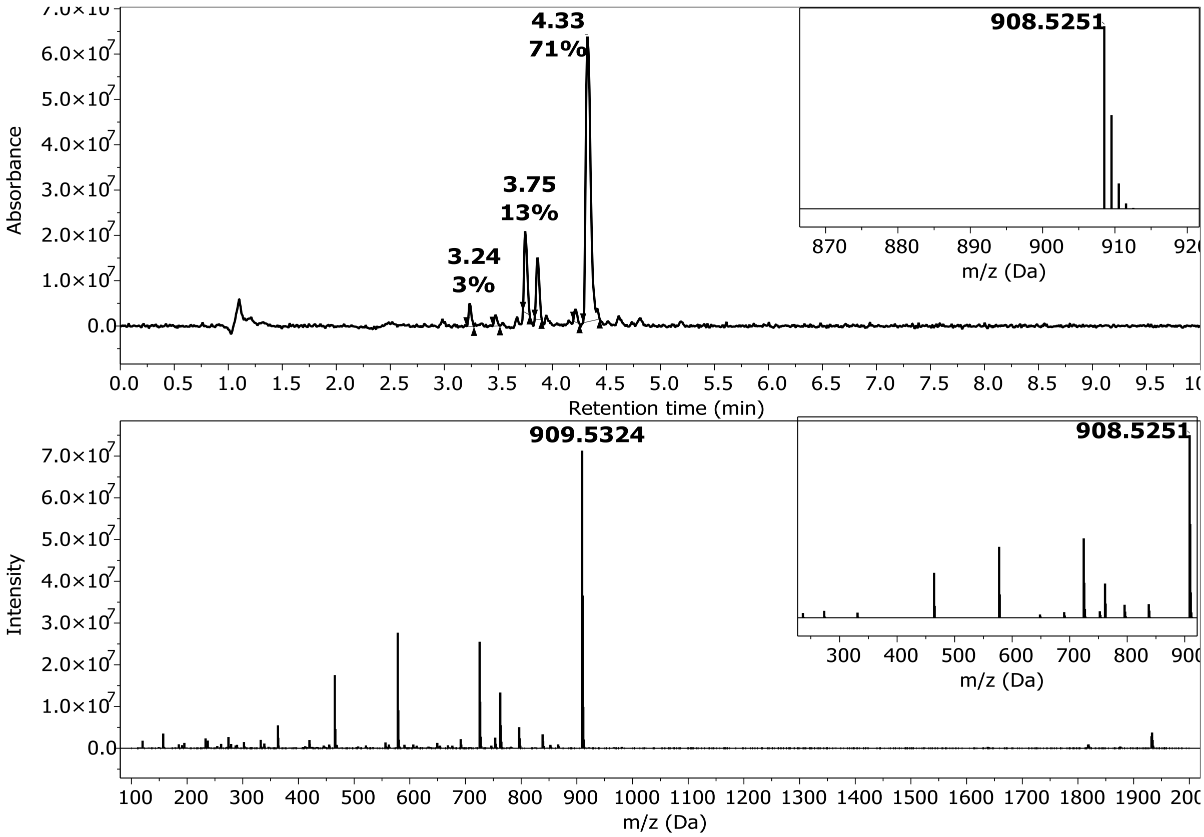

SI Figure 27: LCMS Profile of crude ALFLYRAG. Absorbance chromatogram (λ = 214 nm) of ALFLYRAG; R_t 4.33 min, 71% purity. ESI-TOF spectrum found within R_t 2–8 min (insert: deconvoluted masses). Monoisotopic mass (ESI+) calcd. for $C_{44}H_{68}N_{12}O_9$ 908.5232, found 908.5251. LCMS Gradient A.

8.1.4 Fourth experiment, t = 35 days

The sequence ALF was synthesized on a previously synthesized LYRAG-tag (LYRAG TAG 1, 11.0 mg, 4.5 µmol) using the immobilized base-SPPS standard protocol (Section SI3). Cleavage of the peptidyl resin according to Cleavage Protocol (Section 1.3) afforded the crude peptide (52% purity by LCMS [SI Figure 28]).

AA	m(AA) [mg]	Immob. base	Flow rate [mL/min]	Base temp. [°C]	Reactor temp. [°C]	Activator	t_{sw} [min]	t_{fw} [min]
A	62.0	DIPA 1	0.6	90	90	HATU	4	2
L	70.2	DIPA 1	0.6	90	90	HATU	4	2
F	77.2	DIPA 1	0.6	90	90	HATU	4	2

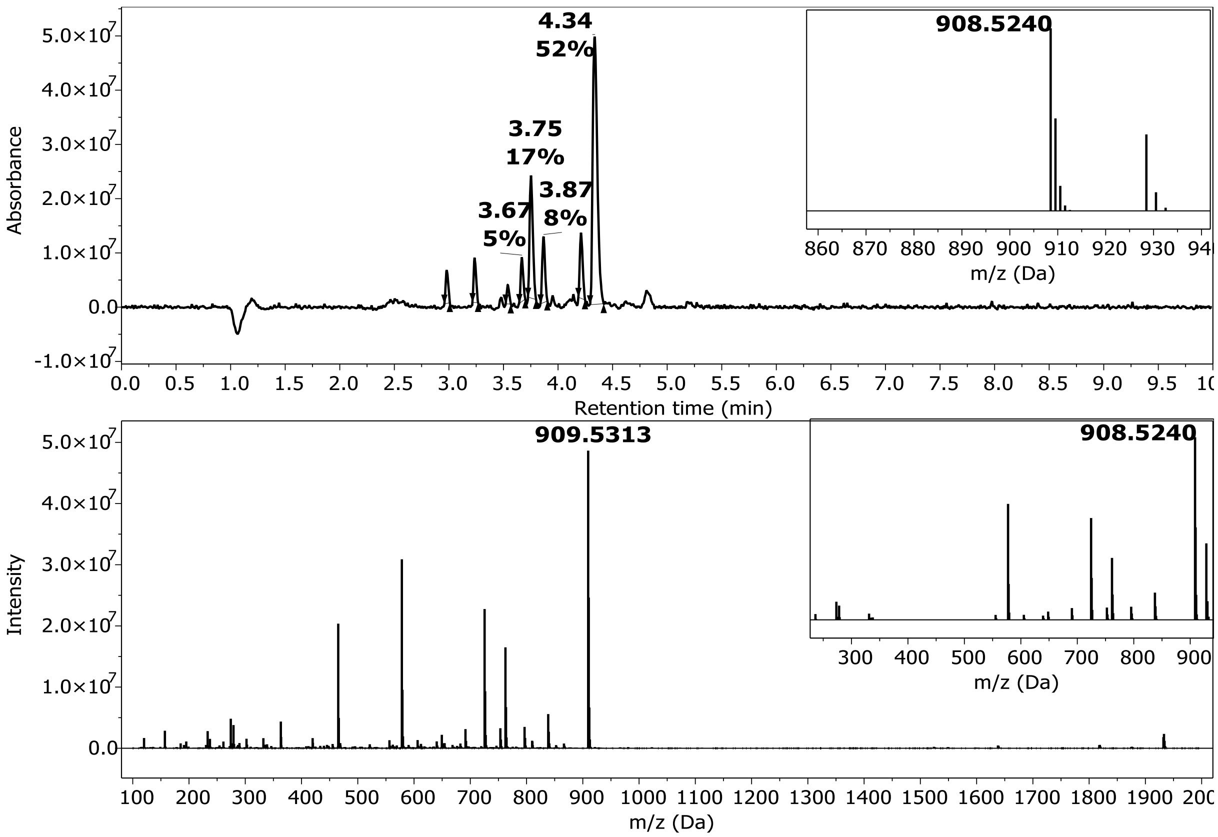

SI Figure 28: LCMS Profile of crude ALFLYRAG. Absorbance chromatogram (λ = 214 nm) of ALFLYRAG; R_t 4.34 min, 57% purity. ESI-TOF spectrum found within R_t 2–8 min (insert: deconvoluted masses). Monoisotopic mass (ESI+) calcd. for $C_{44}H_{68}N_{12}O_9$ 908.5232, found 908.5240. LCMS Gradient A.

8.2 Washed with methanol and DCM, dried and stored in vacuo

8.2.1 First experiment, t = 0 days

The sequence ALF was synthesized on a previously synthesized LYRAG-tag (LYRAG TAG 2, 11.6 mg, 4.8 µmol) using the immobilized base-SPPS standard protocol (Section SI3). Cleavage of the peptidyl resin according to Cleavage Protocol (Section 1.3) afforded the crude peptide (94% purity by LCMS [SI Figure 29]).

AA	m(AA) [mg]	Immob. base	Flow rate [mL/min]	Base temp. [°C]	Reactor temp. [°C]	Activator	t_{sw} [min]	t_{fw} [min]
A	63.6	DIPA 2	0.6	90	90	HATU	4	2
L	71.4	DIPA 2	0.6	90	90	HATU	4	2
F	78.5	DIPA 2	0.6	90	90	HATU	4	2

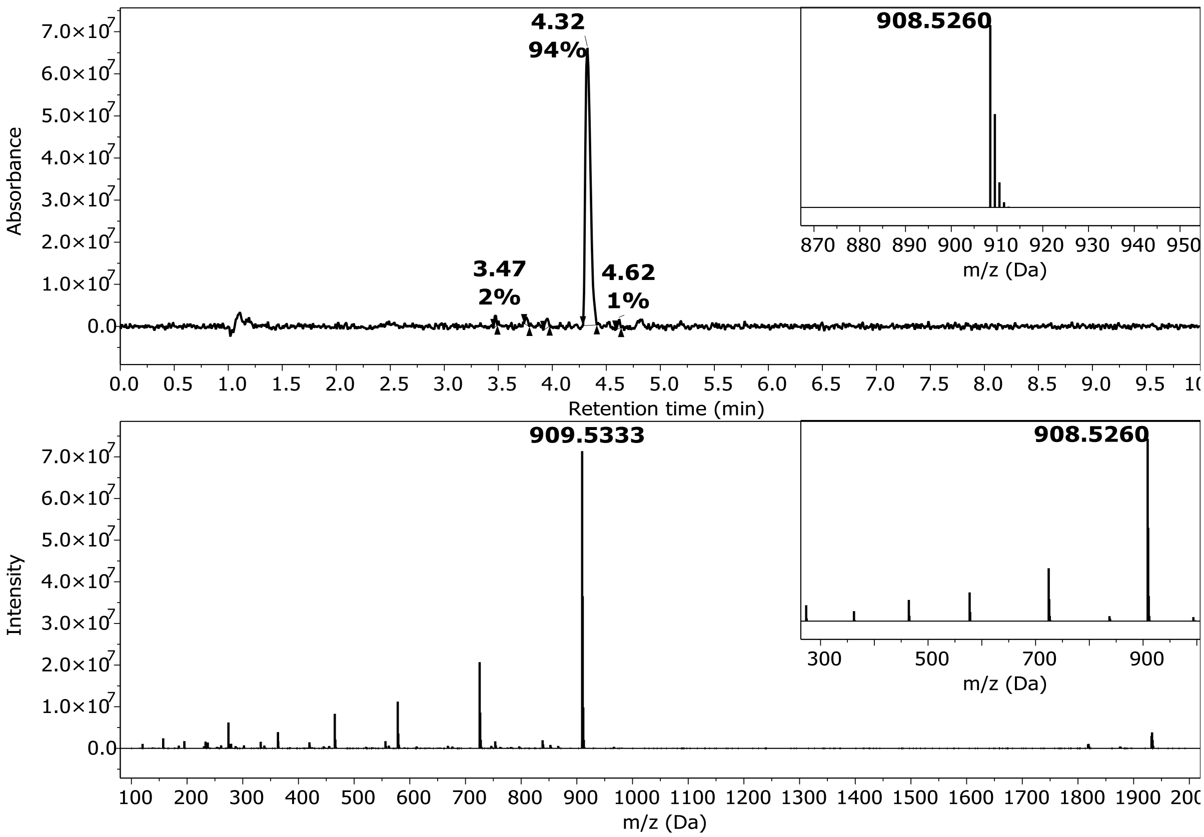

SI Figure 29: LCMS Profile of crude ALFLYRAG. Absorbance chromatogram (λ = 214 nm) of ALFLYRAG; R_t 4.32 min, 94% purity. ESI-TOF spectrum found within R_t 2–8 min (insert: deconvoluted masses). Monoisotopic mass (ESI+) calcd. for $C_{44}H_{68}N_{12}O_9$ 908.5232, found 908.5260. LCMS Gradient A.

8.2.2 Second experiment, t = 6 days

The sequence ALF was synthesized on a previously synthesized LYRAG-tag (LYRAG TAG 2, 9.6 mg, 3.9 μmol) using the immobilized base-SPPS standard protocol (Section SI3). Cleavage of the peptidyl resin according to Cleavage Protocol (Section 1.3) afforded the crude peptide (46% purity by LCMS [SI Figure 30]).

AA	m(AA) [mg]	Immob. base	Flow rate [mL/min]	Base temp. [°C]	Reactor temp. [°C]	Activator	t_{sw} [min]	t_{fw} [min]
A	62.0	DIPA 2	0.6	90	90	HATU	4	2
L	71.1	DIPA 2	0.6	90	90	HATU	4	2
F	77.0	DIPA 2	0.6	90	90	HATU	4	2

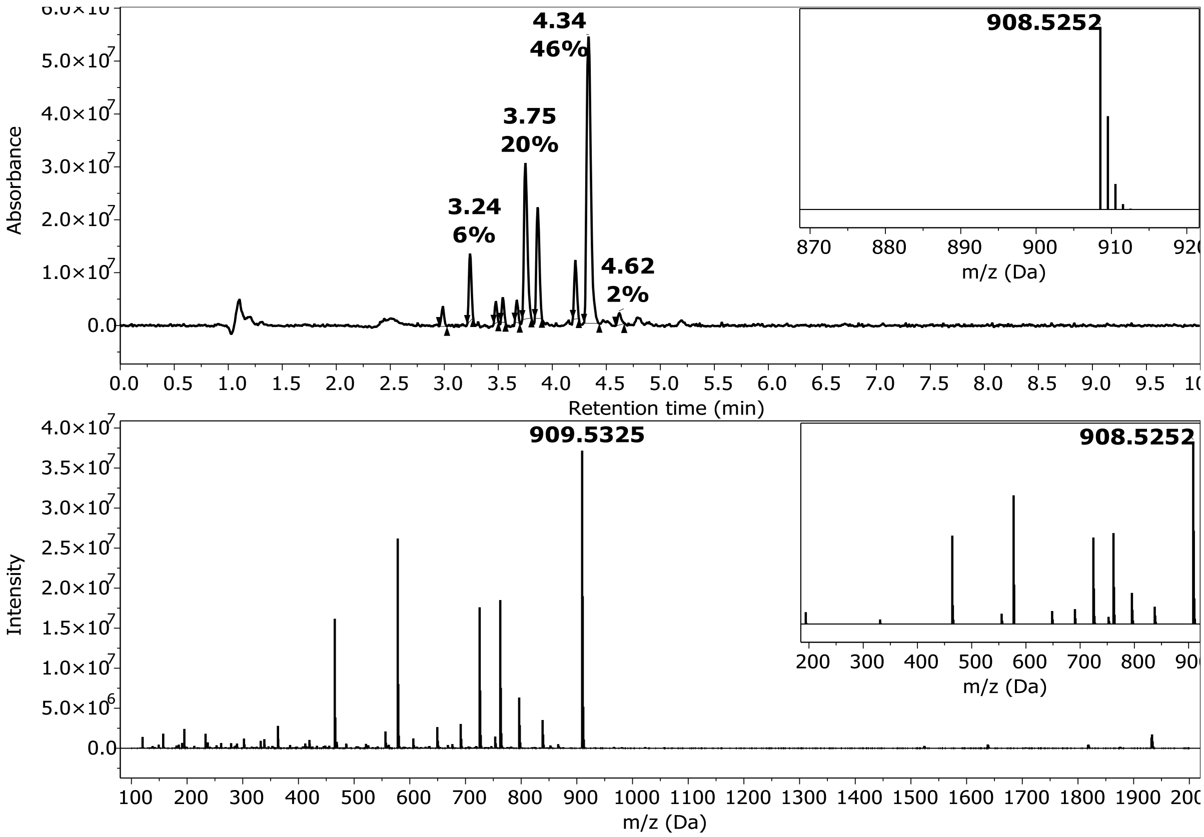

SI Figure 30: LCMS Profile of crude ALFLYRAG. Absorbance chromatogram (λ = 214 nm) of ALFLYRAG; R_t 4.34 min, 46% purity. ESI-TOF spectrum found within R_t 2–8 min (insert: deconvoluted masses). Monoisotopic mass (ESI+) calcd. for $C_{44}H_{68}N_{12}O_9$ 908.5232, found 908.5252. LCMS Gradient A.

8.3 Washed with DCM, stored in vacuo

8.3.1 First experiment, t = 0 days

The sequence ALF was synthesized on a previously synthesized LYRAG-tag (LYRAG TAG 2, 10.2 mg, 4.2 μmol) using the immobilized base-SPPS standard protocol (Section SI3). Cleavage of the peptidyl resin according to Cleavage Protocol (Section 1.3) afforded the crude peptide (94% purity by LCMS [SI Figure 31]).

AA	m(AA) [mg]	Immob. base	Flow rate [mL/min]	Base temp. [°C]	Reactor temp. [°C]	Activator	t_{sw} [min]	t_{fw} [min]
A	61.8	DIPA 3	0.6	90	90	HATU	4	2
L	71.4	DIPA 3	0.6	90	90	HATU	4	2
F	77.5	DIPA 3	0.6	90	90	HATU	4	2

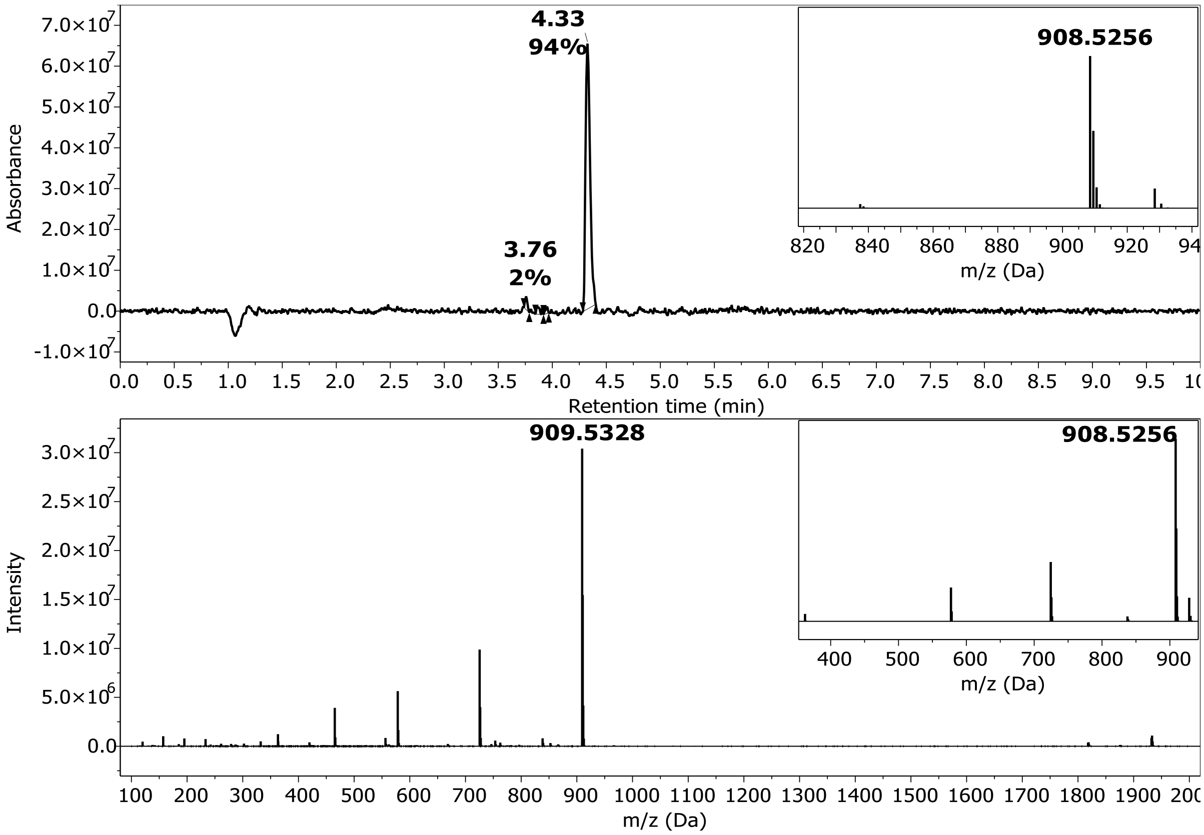

SI Figure 31: LCMS Profile of crude ALFLYRAG. Absorbance chromatogram (λ = 214 nm) of ALFLYRAG; R_t 4.33 min, 94% purity. ESI-TOF spectrum found within R_t 2–8 min (insert: deconvoluted masses). Monoisotopic mass (ESI+) calcd. for $C_{44}H_{68}N_{12}O_9$ 908.5232, found 908.5256. LCMS Gradient A.

8.3.2 Second experiment, t = 6 days

The sequence ALF was synthesized on a previously synthesized LYRAG-tag (LYRAG TAG 2, 11.2 mg, 4.6 µmol) using the immobilized base-SPPS standard protocol (Section SI3). Cleavage of the peptidyl resin according to Cleavage Protocol (Section 1.3) afforded the crude peptide (62% purity by LCMS [SI Figure 32]).

AA	m(AA) [mg]	Immob. base	Flow rate [mL/min]	Base temp. [°C]	Reactor temp. [°C]	Activator	t_{sw} [min]	t_{fw} [min]
A	63.0	DIPA 3	0.6	90	90	HATU	4	2
L	71.1	DIPA 3	0.6	90	90	HATU	4	2
F	77.7	DIPA 3	0.6	90	90	HATU	4	2

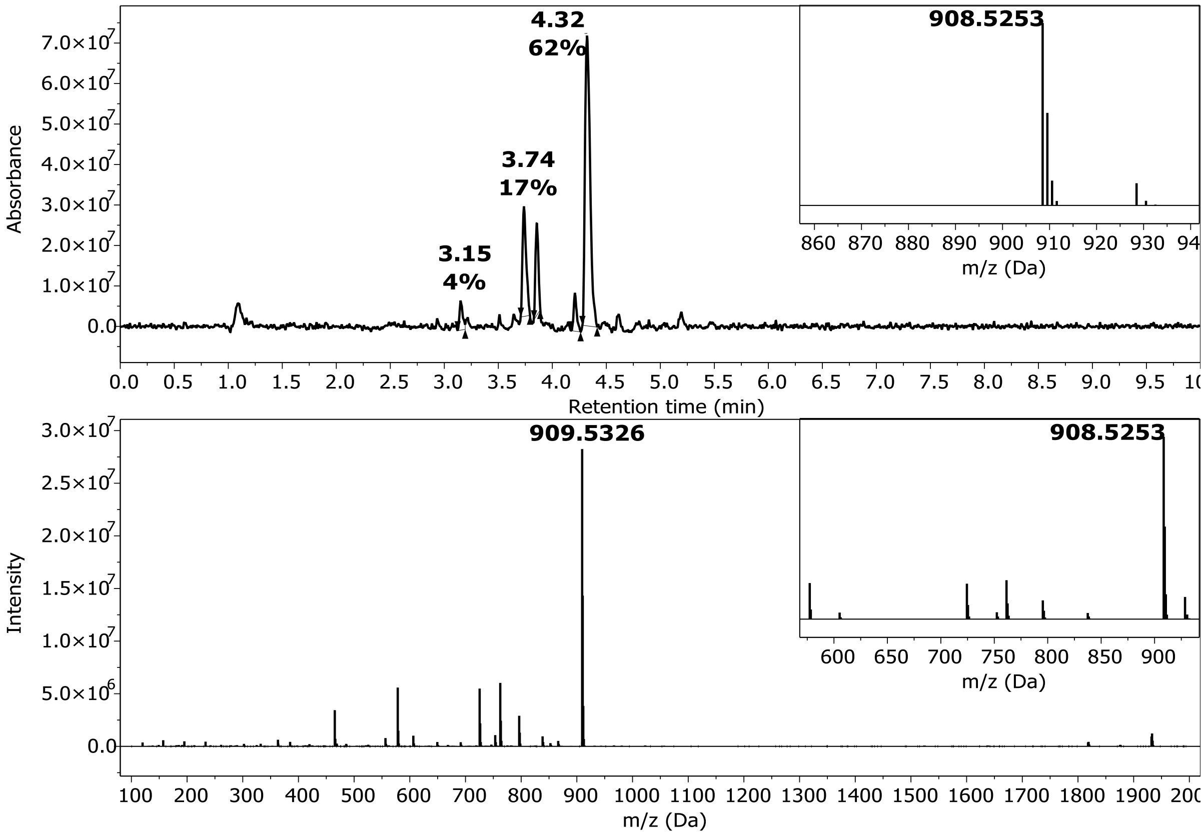

SI Figure 32: LCMS Profile of crude ALFLYRAG. Absorbance chromatogram (λ = 214 nm) of ALFLYRAG; R_t 4.32 min, 63% purity. ESI-TOF spectrum found within R_t 2–8 min (insert: deconvoluted masses). Monoisotopic mass (ESI+) calcd. for $C_{44}H_{68}N_{12}O_9$ 908.5232, found 908.5253. LCMS Gradient A.

8.4 Washed with DCM and stored airtight

8.4.1 First experiment, t = 0 days

The sequence ALF was synthesized on a previously synthesized LYRAG-tag (LYRAG TAG 2, 11.5 mg, 4.7 µmol) using the immobilized base-SPPS standard protocol (Section SI3). Cleavage of the peptidyl resin according to Cleavage Protocol (Section 1.3) afforded the crude peptide (96% purity by LCMS [SI Figure 33]).

AA	m(AA) [mg]	Immob. base	Flow rate [mL/min]	Base temp. [°C]	Reactor temp. [°C]	Activator	t_{sw} [min]	t_{fw} [min]
A	63.0	DIPA 4	0.6	90	90	HATU	4	2
L	71.4	DIPA 4	0.6	90	90	HATU	4	2
F	77.6	DIPA 4	0.6	90	90	HATU	4	2

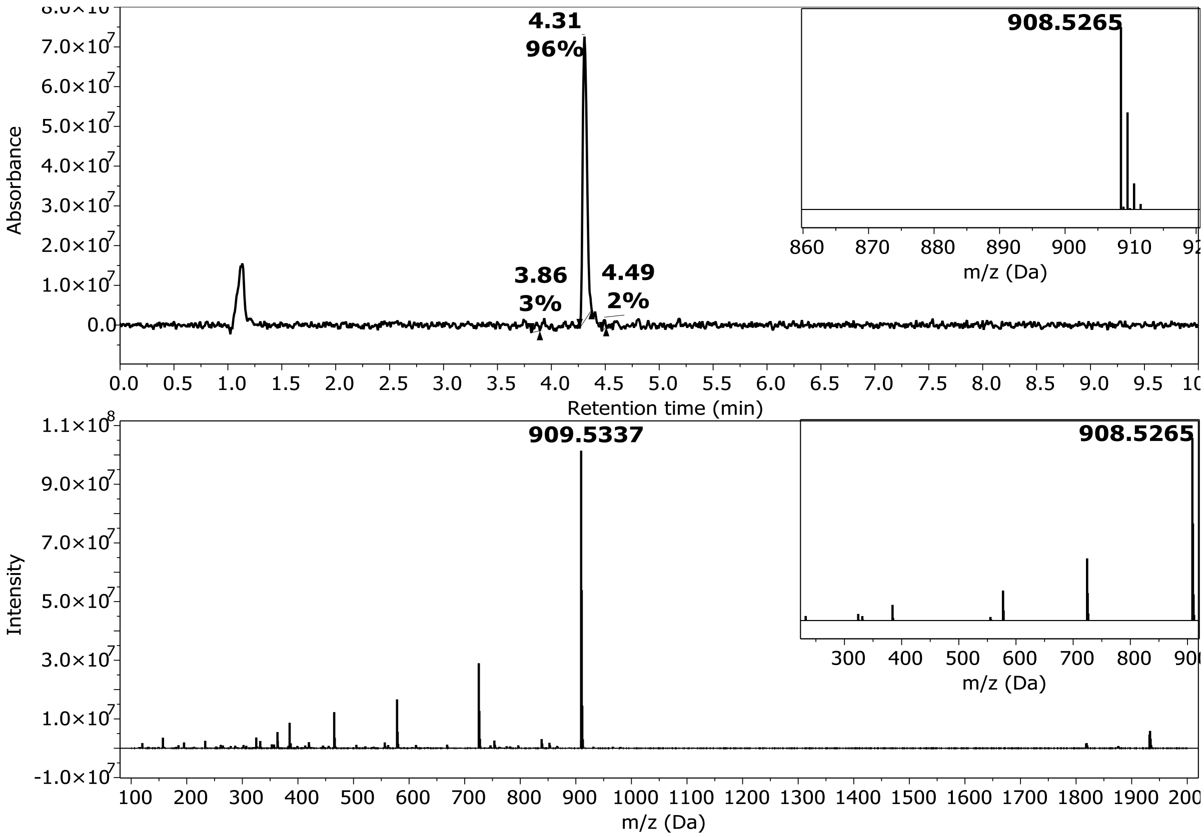

SI Figure 33: LCMS Profile of crude ALFLYRAG. Absorbance chromatogram (λ = 214 nm) of ALFLYRAG; R_t 4.31 min, 96% purity. ESI-TOF spectrum found within R_t 2–8 min (insert: deconvoluted masses). Monoisotopic mass (ESI+) calcd. for $C_{44}H_{68}N_{12}O_9$ 908.5232, found 908.5265. LCMS Gradient A.

8.4.2 Second experiment, t = 1 day

The sequence ALF was synthesized on a previously synthesized LYRAG-tag (LYRAG TAG 2, 11.7 mg, 4.8 μmol) using the immobilized base-SPPS standard protocol (Section SI3). Cleavage of the peptidyl resin according to Cleavage Protocol (Section 1.3) afforded the crude peptide (90% purity by LCMS [SI Figure 34]).

AA	m(AA) [mg]	Immob. base	Flow rate [mL/min]	Base temp. [°C]	Reactor temp. [°C]	Activator	t_{sw} [min]	t_{fw} [min]
A	62.9	DIPA 4	0.6	90	90	HATU	4	2
L	70.8	DIPA 4	0.6	90	90	HATU	4	2
F	77.0	DIPA 4	0.6	90	90	HATU	4	2

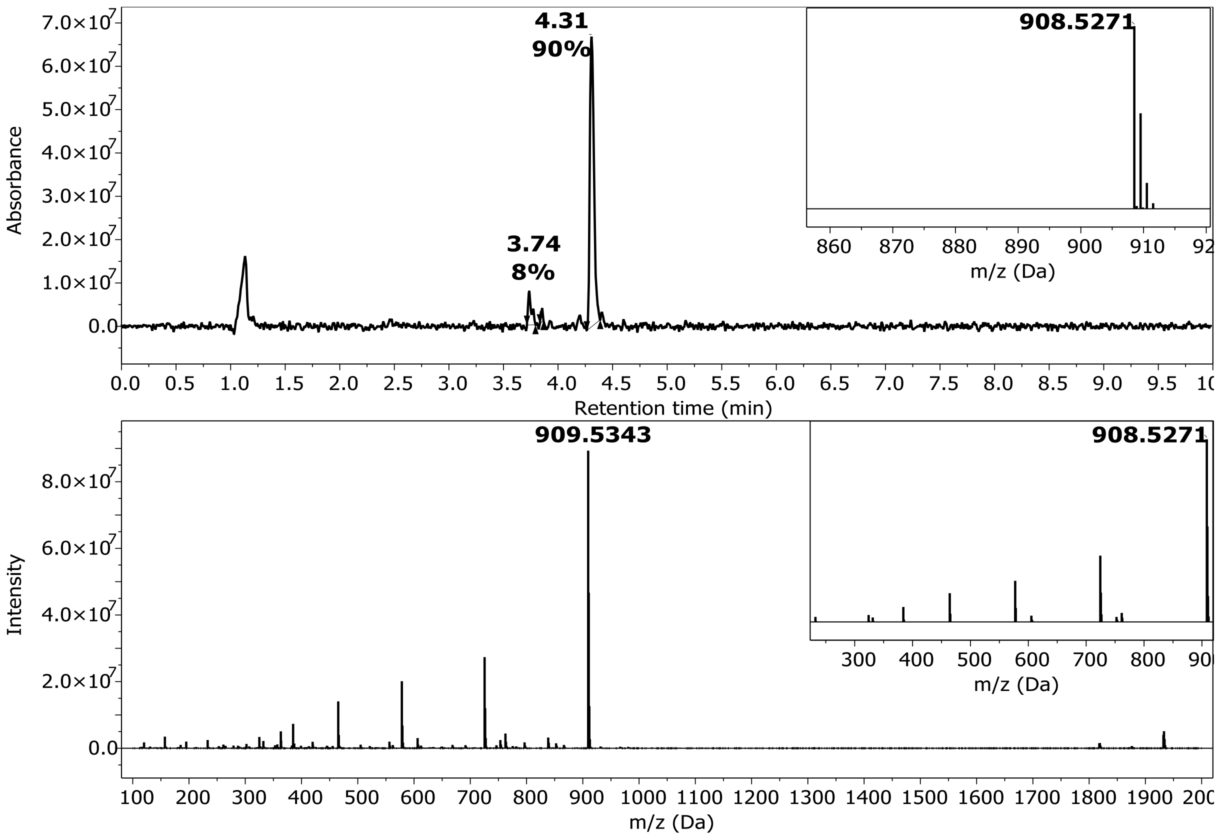

SI Figure 34: LCMS Profile of crude ALFLYRAG. Absorbance chromatogram (λ = 214 nm) of ALFLYRAG; R_t 4.31 min, 90% purity. ESI-TOF spectrum found within R_t 2–8 min (insert: deconvoluted masses). Monoisotopic mass (ESI+) calcd. for $C_{44}H_{68}N_{12}O_9$ 908.5232, found 908.5271. LCMS Gradient A.

8.4.3 Third experiment, t = 3 days

The sequence ALF was synthesized on a previously synthesized LYRAG-tag (LYRAG TAG 3, 9.9 mg, 4.1 µmol) using the immobilized base-SPPS standard protocol (Section SI3). Cleavage of the peptidyl resin according to Cleavage Protocol (Section 1.3) afforded the crude peptide (91% purity by LCMS [SI Figure 35]).

AA	m(AA) [mg]	Immob. base	Flow rate [mL/min]	Base temp. [°C]	Reactor temp. [°C]	Activator	t_{sw} [min]	t_{fw} [min]
A	62.4	DIPA 4	0.6	90	90	HATU	4	2
L	71.3	DIPA 4	0.6	90	90	HATU	4	2
F	77.7	DIPA 4	0.6	90	90	HATU	4	2

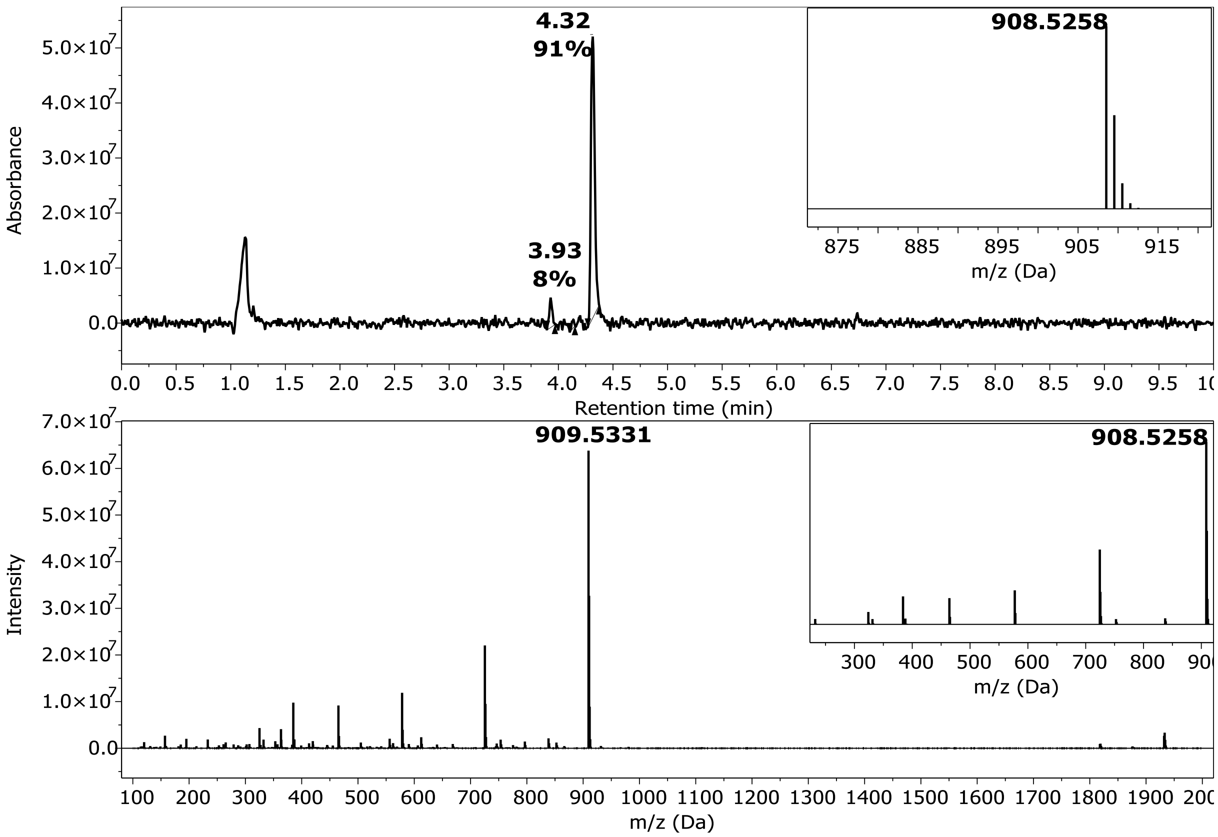

SI Figure 35: LCMS Profile of crude ALFLYRAG. Absorbance chromatogram (λ = 214 nm) of ALFLYRAG; R_t 4.32 min, 91% purity. ESI-TOF spectrum found within R_t 2–8 min (insert: deconvoluted masses). Monoisotopic mass (ESI+) calcd. for $C_{44}H_{68}N_{12}O_9$ 908.5232, found 908.5258. LCMS Gradient A.

8.4.4 Fourth experiment, t = 13 days

The sequence ALF was synthesized on a previously synthesized LYRAG-tag (LYRAG TAG 3, 9.2 mg, 3.8 μmol) using the immobilized base-SPPS standard protocol (Section SI3). Cleavage of the peptidyl resin according to Cleavage Protocol (Section 1.3) afforded the crude peptide (86% purity by LCMS [SI Figure 36]).

AA	m(AA) [mg]	Immob. base	Flow rate [mL/min]	Base temp. [°C]	Reactor temp. [°C]	Activator	t_{sw} [min]	t_{fw} [min]
A	63.5	DIPA 4	0.6	90	90	HATU	4	2
L	71.6	DIPA 4	0.6	90	90	HATU	4	2
F	77.9	DIPA 4	0.6	90	90	HATU	4	2

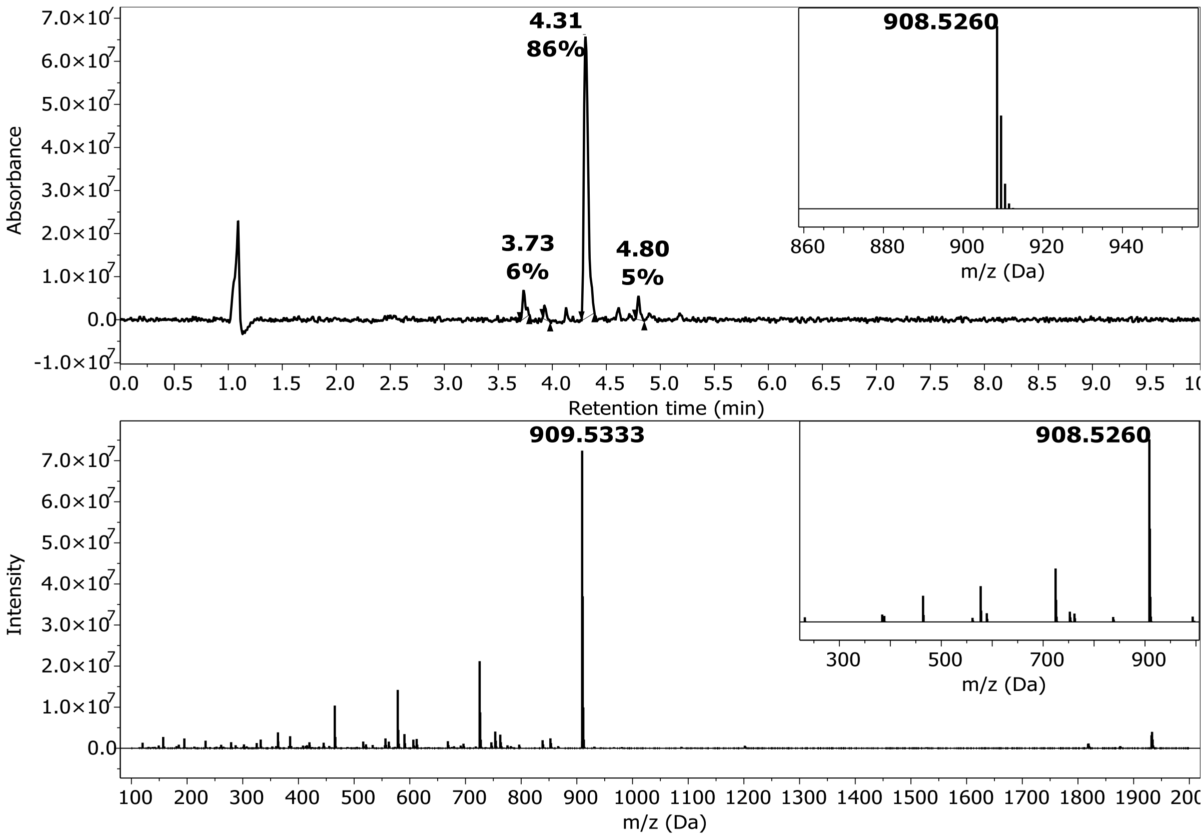

SI Figure 36: LCMS Profile of crude ALFLYRAG. Absorbance chromatogram (λ = 214 nm) of ALFLYRAG; R_t 4.31 min, 86% purity. ESI-TOF spectrum found within R_t 2–8 min (insert: deconvoluted masses). Monoisotopic mass (ESI+) calcd. for $C_{44}H_{68}N_{12}O_9$ 908.5232, found 908.5260. LCMS Gradient A.

8.5 Washed with DMF and stored airtight

8.5.1 First experiment, t = 0 days

The sequence ALF was synthesized on a previously synthesized LYRAG-tag (LYRAG TAG 4, 11.1 mg, 4.6 µmol) using the immobilized base-SPPS standard protocol (Section SI3). Cleavage of the peptidyl resin according to Cleavage Protocol (Section 1.3) afforded the crude peptide (94% purity by LCMS [SI Figure 37]).

AA	m(AA) [mg]	Immob. base	Flow rate [mL/min]	Base temp. [°C]	Reactor temp. [°C]	Activator	t_{sw} [min]	t_{fw} [min]
A	63.1	DIPA 7	0.6	90	90	HATU	4	2
L	71.1	DIPA 7	0.6	90	90	HATU	4	2
F	78.5	DIPA 7	0.6	90	90	HATU	4	2

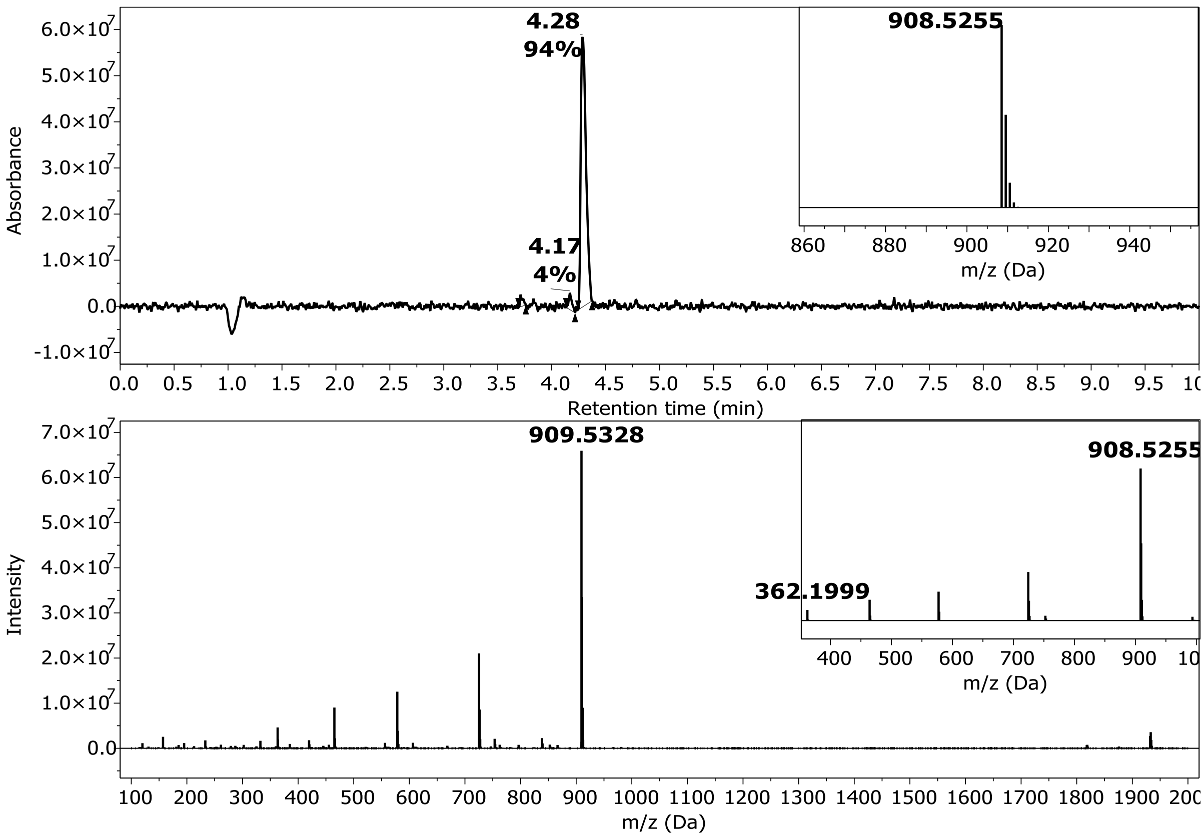

SI Figure 37: LCMS Profile of crude ALFLYRAG. Absorbance chromatogram (λ = 214 nm) of ALFLYRAG; R_t 4.28 min, 94% purity. ESI-TOF spectrum found within R_t 2–8 min (insert: deconvoluted masses). Monoisotopic mass (ESI+) calcd. for $C_{44}H_{68}N_{12}O_9$ 908.5232, found 908.5255. LCMS Gradient A.

8.5.2 Second experiment, t = 6 days

The sequence ALF was synthesized on a previously synthesized LYRAG-tag (LYRAG TAG 4, 9.1 mg, 3.7 µmol) using the immobilized base-SPPS standard protocol (Section SI3). Cleavage of the peptidyl resin according to Cleavage Protocol (Section 1.3) afforded the crude peptide (95% purity by LCMS [SI Figure 38]).

AA	m(AA) [mg]	Immob. base	Flow rate [mL/min]	Base temp. [°C]	Reactor temp. [°C]	Activator	t_{sw} [min]	t_{fw} [min]
A	63.7	DIPA 7	0.6	90	90	HATU	4	2
L	71.5	DIPA 7	0.6	90	90	HATU	4	2
F	76.6	DIPA 7	0.6	90	90	HATU	4	2

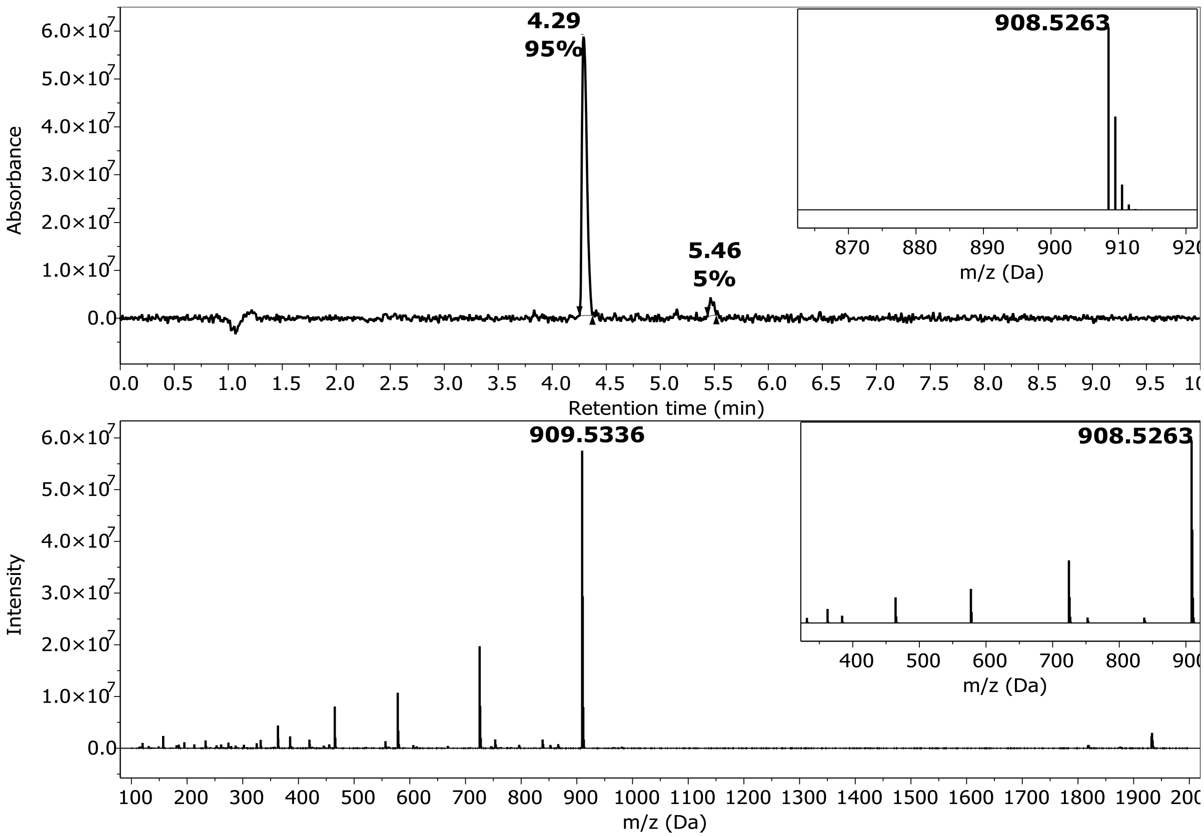

SI Figure 38: LCMS Profile of crude ALFLYRAG. Absorbance chromatogram (λ = 214 nm) of ALFLYRAG; R_t 4.29 min, 95% purity. ESI-TOF spectrum found within R_t 2–8 min (insert: deconvoluted masses). Monoisotopic mass (ESI+) calcd. for $C_{44}H_{68}N_{12}O_9$ 908.5232, found 908.5263. LCMS Gradient A.

8.5.3 Third experiment, t = 8 days

The sequence ALF was synthesized on a previously synthesized LYRAG-tag (LYRAG TAG 4, 11.5 mg, 4.7 µmol) using the immobilized base-SPPS standard protocol (Section SI3). Cleavage of the peptidyl resin according to Cleavage Protocol (Section 1.3) afforded the crude peptide (95% purity by LCMS [SI Figure 39]).

AA	m(AA) [mg]	Immob. base	Flow rate [mL/min]	Base temp. [°C]	Reactor temp. [°C]	Activator	t_{sw} [min]	t_{fw} [min]
A	62.8	DIPA 7	0.6	90	90	HATU	4	2
L	71.6	DIPA 7	0.6	90	90	HATU	4	2
F	77.1	DIPA 7	0.6	90	90	HATU	4	2

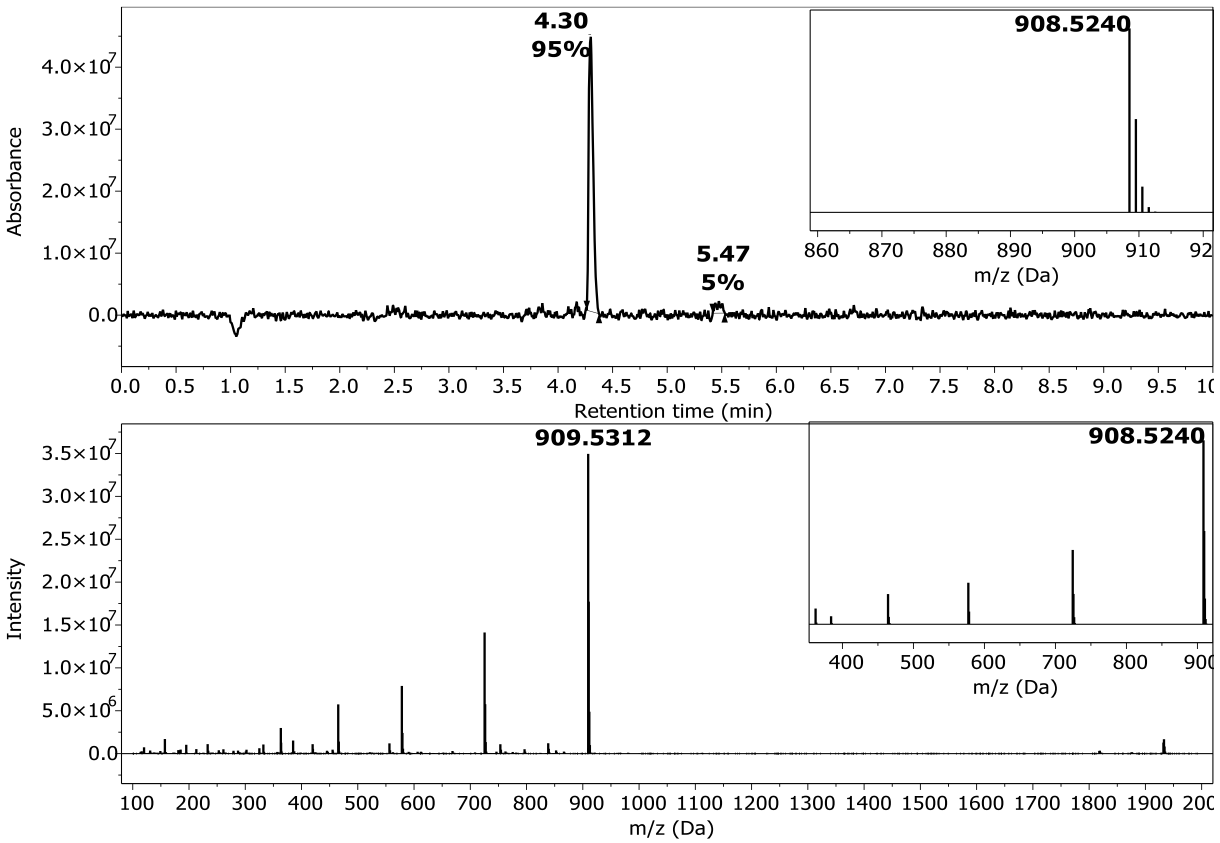

SI Figure 39: LCMS Profile of crude ALFLYRAG. Absorbance chromatogram (λ = 214 nm) of ALFLYRAG; R_t 4.30 min, 95% purity. ESI-TOF spectrum found within R_t 2–8 min (insert: deconvoluted masses). Monoisotopic mass (ESI+) calcd. for $C_{44}H_{68}N_{12}O_9$ 908.5232, found 908.5240. LCMS Gradient A.

8.5.4 Fourth experiment, t = 20 days

The sequence ALF was synthesized on a previously synthesized LYRAG-tag (LYRAG TAG 4, 11.0 mg, 4.5 μmol) using the immobilized base-SPPS standard protocol (Section SI3). Cleavage of the peptidyl resin according to Cleavage Protocol (Section 1.3) afforded the crude peptide (93% purity by LCMS [SI Figure 40]).

AA	m(AA) [mg]	Immob. base	Flow rate [mL/min]	Base temp. [°C]	Reactor temp. [°C]	Activator	t_{sw} [min]	t_{fw} [min]
A	62.2	DIPA 7	0.6	90	90	HATU	4	2
L	71.9	DIPA 7	0.6	90	90	HATU	4	2
F	79.0	DIPA 7	0.6	90	90	HATU	4	2

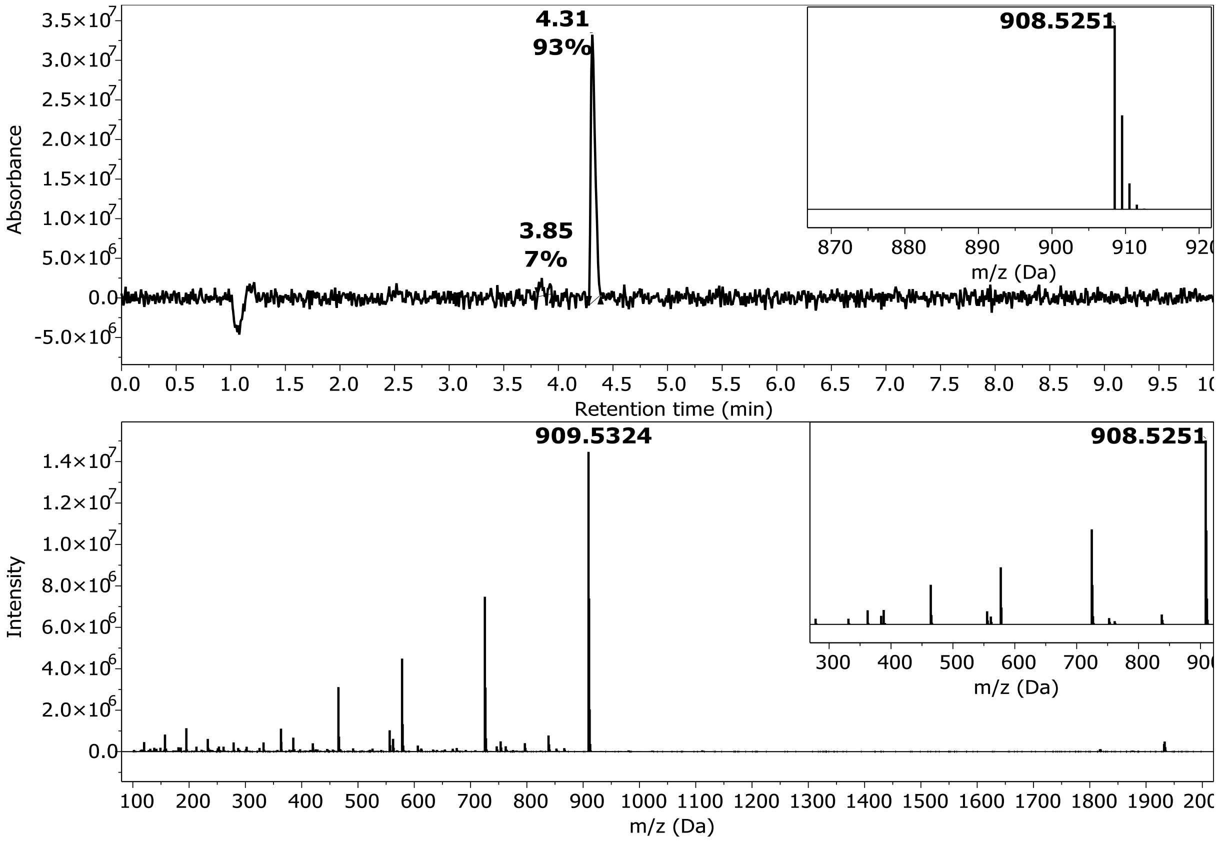

SI Figure 40: LCMS Profile of crude ALFLYRAG. Absorbance chromatogram (λ = 214 nm) of ALFLYRAG; R_t 4.31 min, 93% purity. ESI-TOF spectrum found within R_t 2–8 min (insert: deconvoluted masses). Monoisotopic mass (ESI+) calcd. for $C_{44}H_{68}N_{12}O_9$ 908.5232, found 908.5251. LCMS Gradient A.

8.5.5 Fifth experiment, t = 49 days

The sequence ALF was synthesized on a previously synthesized LYRAG-tag (LYRAG TAG 6, 9.8 mg, 4.0 μmol) using the immobilized base-SPPS standard protocol (Section SI3). Cleavage of the peptidyl resin according to Cleavage Protocol (Section 1.3) afforded the crude peptide (2 mg, 87% purity by LCMS [SI Figure 41])

AA	m(AA) [mg]	Immob. base	Flow rate [mL/min]	Base temp. [°C]	Reactor temp. [°C]	Activator	t_{sw} [min]	t_{fw} [min]
A	64.6	DIPA 7	0.6	90	90	HATU	4	2
L	71.5	DIPA 7	0.6	90	90	HATU	4	2
F	78.1	DIPA 7	0.6	90	90	HATU	4	2

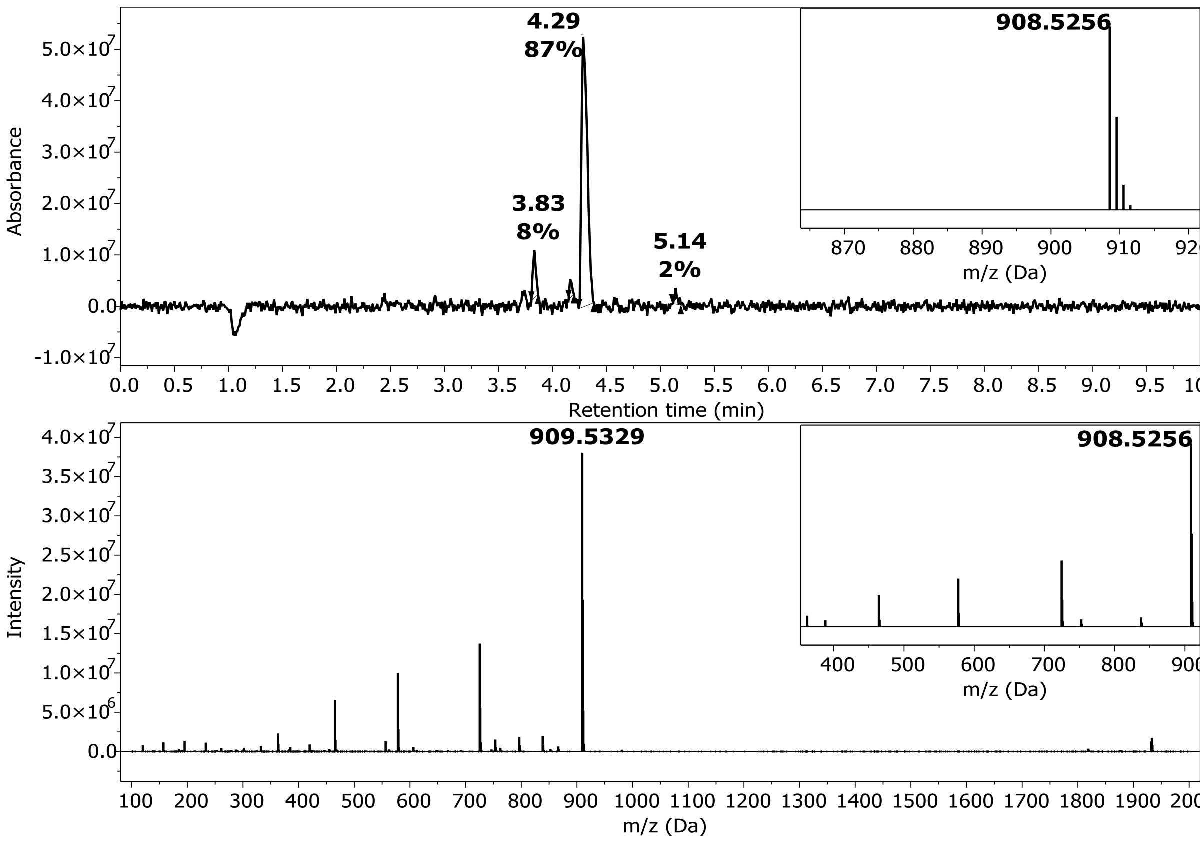

SI Figure 41: LCMS Profile of crude ALFLYRAG. Absorbance chromatogram (λ = 214 nm) of ALFLYRAG; R_t 4.29 min, 87% purity. ESI-TOF spectrum found within R_t 2–8 min (insert: deconvoluted masses). Monoisotopic mass (ESI+) calcd. for $C_{44}H_{68}N_{12}O_9$ 908.5232, found 908.5256. LCMS Gradient A.

8.6 Storage sealed in -20°C freezer

8.6.1 After 12 months

The sequence ALF was synthesized on a previously synthesized LYRAG-tag (LYRAG TAG 6, 11.0 mg, 4.0 µmol) using the immobilized base-SPPS standard protocol (Section SI3). Cleavage of the peptidyl resin according to Cleavage Protocol (Section 1.3) afforded the crude peptide (1.8 mg, 92% purity by LCMS [SI Figure 42] , 91% purity by UHPLC [SI Figure 43])

AA	m(AA) [mg]	Immob. base	Flow rate [mL/min]	Base temp. [°C]	Reactor temp. [°C]	Activator	t_{sw} [min]	t_{fw} [min]
A	67.1	DIPA 11	0.6	90	90	HATU	4	2
L	72.1	DIPA 11	0.6	90	90	HATU	4	2
F	78.4	DIPA 11	0.6	90	90	HATU	4	2

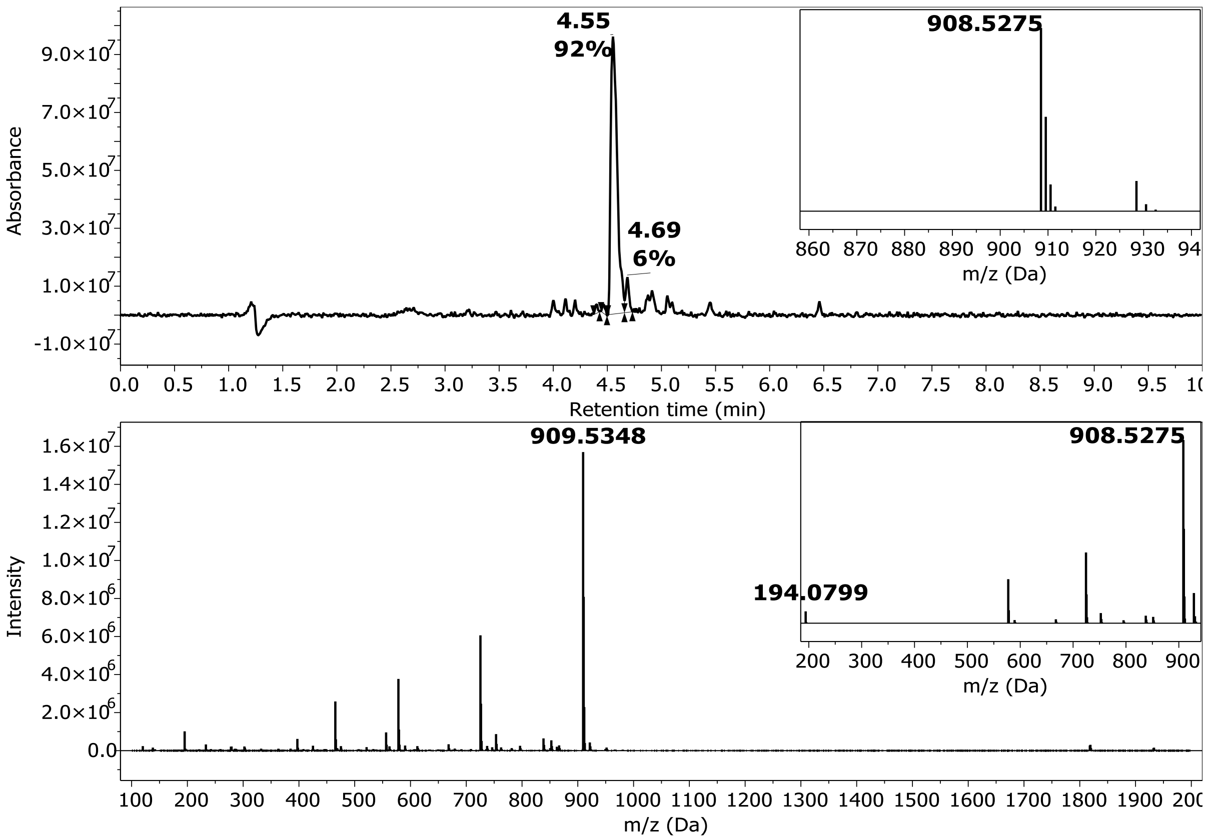

SI Figure 42: LCMS Profile of crude ALFLYRAG. Absorbance chromatogram (λ = 214 nm) of ALFLYRAG; R_t 4.55 min, 92% purity. ESI-TOF spectrum found within R_t 2–8 min (insert: deconvoluted masses). Monoisotopic mass (ESI+) calcd. for $C_{44}H_{68}N_{12}O_9$ 908.5232, found 908.5275. LCMS Gradient A.

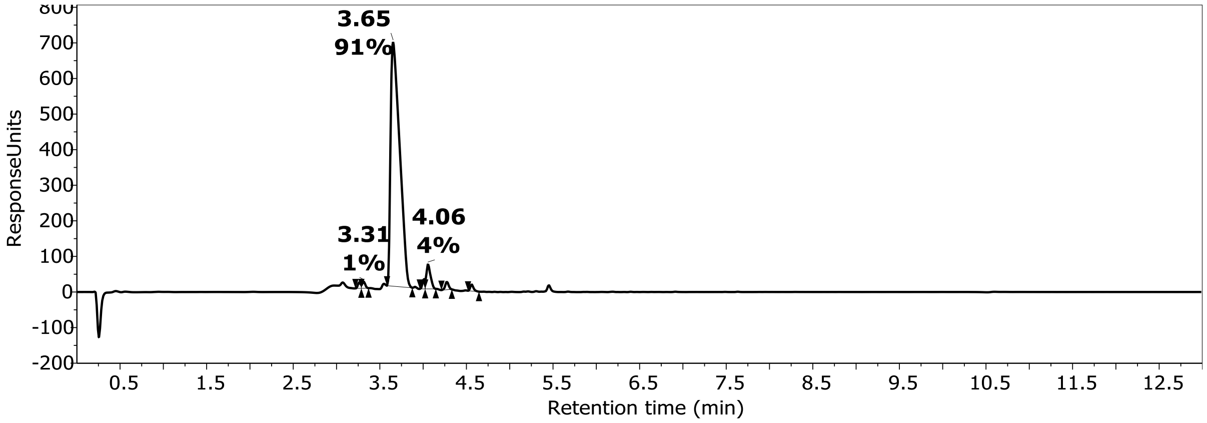

SI Figure 43: UHPLC profile of crude ALFLYRAG. R_t 3.65 min. 91% purity based on Area Under Curve (AUC) at λ = 214 nm.

9 Optimization of all canonical amino acids

9.1 A, F, L

Experiment presented in 3.1.2.

9.2 C, G

Experiment presented in 3.2.2.

9.3 D, E

9.3.1 Standard conditions

The sequence DEI was synthesized on a previously synthesized LYRAG-tag (LYRAG TAG 3, 8.70 mg, 3.6 μmol) using the immobilized base-SPPS standard protocol (Section SI3). Cleavage of the peptidyl resin according to Cleavage Protocol (Section 1.3) afforded the crude peptide (1.1 mg, 74% purity by LCMS [SI Figure 44]).

AA	m(AA) [mg]	Immob. base	Flow rate [mL/min]	Base temp. [°C]	Reactor temp. [°C]	Activator	t_{sw} [min]	t_{fw} [min]
D	83.6	DIPA 5	0.6	90	90	HATU	4	2
E	89.1	DIPA 5	0.6	90	90	HATU	4	2
I	71.6	DIPA 5	0.6	90	90	HATU	4	2

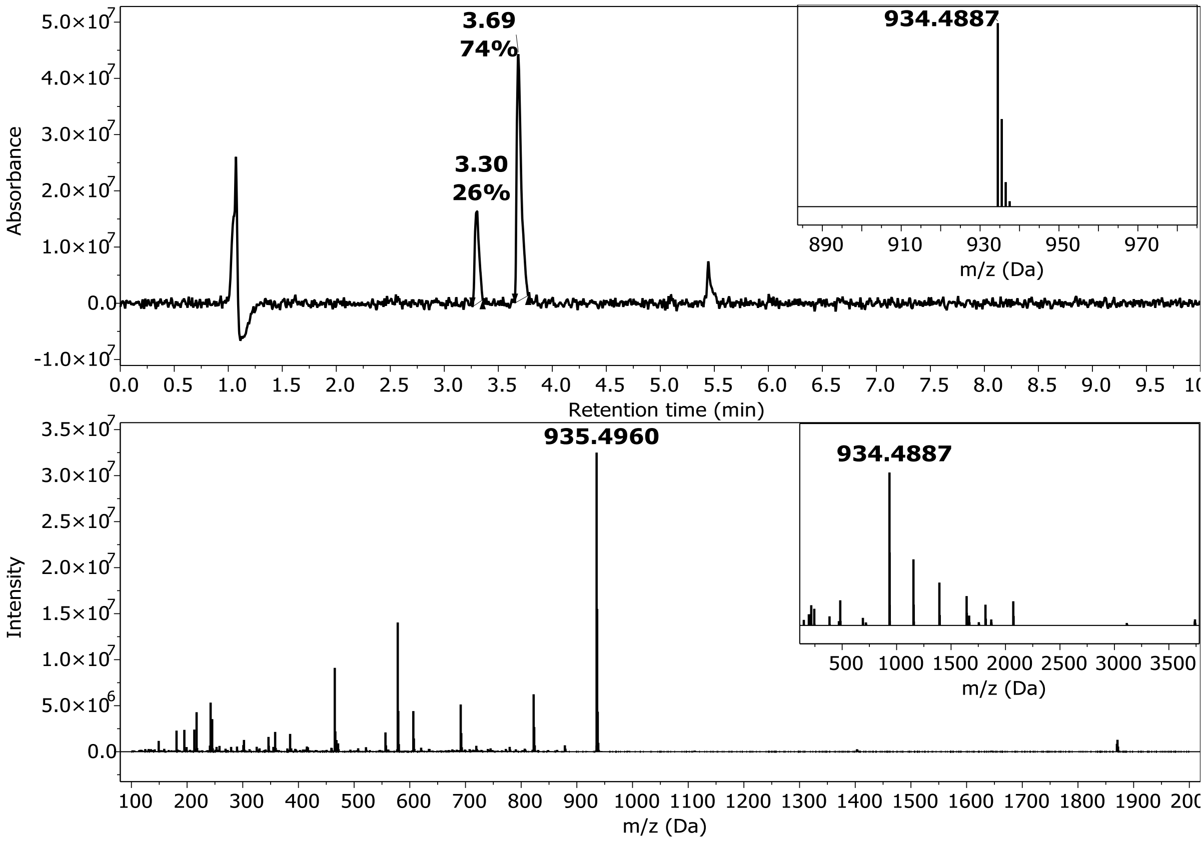

SI Figure 44: LCMS Profile of crude DEILYRAG. Absorbance chromatogram (λ = 214 nm) of DEILYRAG; R_t 3.69 min, 74% purity. ESI-TOF spectrum found within R_t 2–8 min (insert: deconvoluted masses). Monoisotopic mass (ESI+) calcd. For $C_{41}H_{66}N_{12}O_{13}$ 934.4872, found 934.4887. LCMS Gradient A.

9.4 H

9.4.1 Standard conditions

Reported in paragraph 3.1.2.

9.4.2 His(Boc)

The sequence FHL was synthesized on a previously synthesized LYRAG-tag (LYRAG TAG 5, 9.9 mg, 3.9 µmol) using the immobilized base-SPPS standard protocol (Section SI3). Cleavage of the peptidyl resin according to Cleavage Protocol (Section 1.3) afforded the crude peptide (1.4 mg, 79% purity by LCMS [SI Figure 45]).

AA	m(AA) [mg]	Immob. base	Flow rate [mL/min]	Base temp. [°C]	Reactor temp. [°C]	Activator	t_{sw} [min]	t_{fw} [min]
F	76.1	DIPA 7	0.6	90	90	HATU	4	2
H(Boc)	94.7	DIPA 7	0.6	90	90	HATU	4	2
L	70.5	DIPA 7	0.6	90	90	HATU	4	2
L/D	No epimerization							

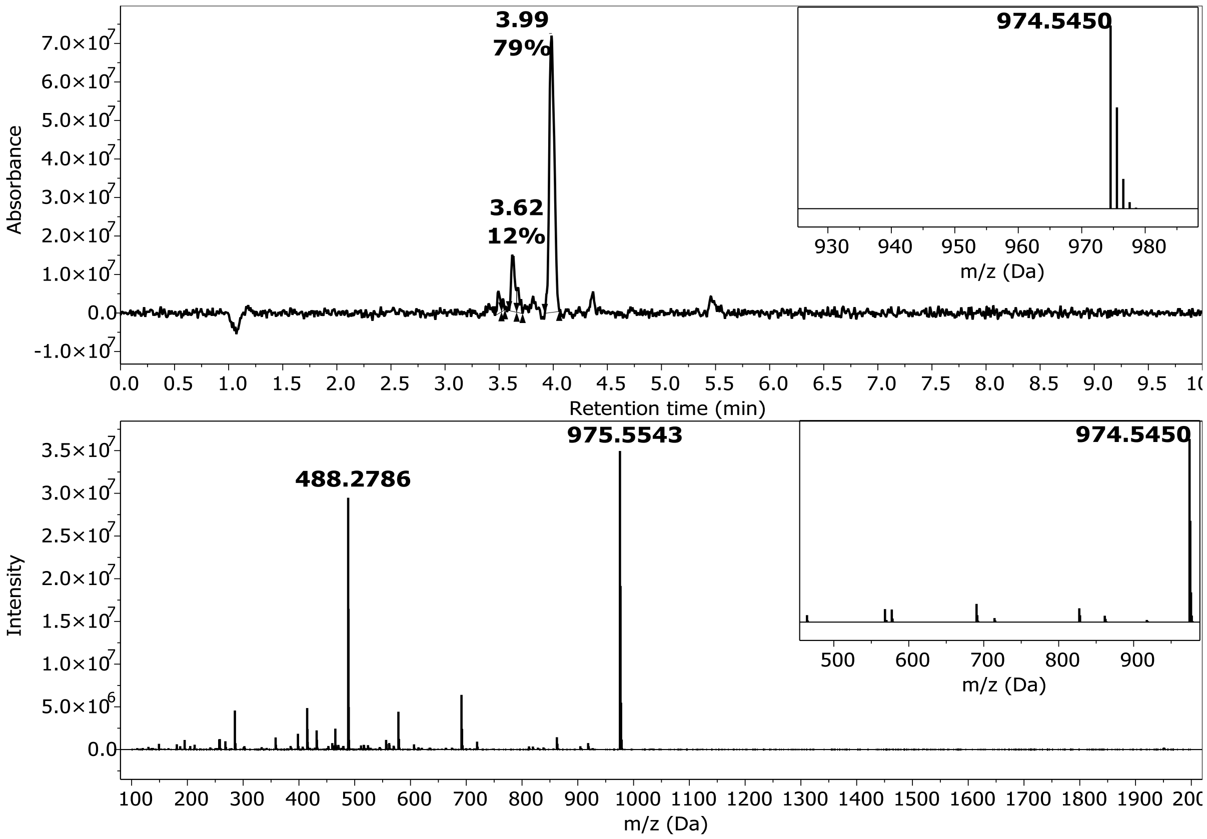

SI Figure 45: LCMS Profile of crude FHLLYRAG. Absorbance chromatogram (λ = 214 nm) of FHLLYRAG; R_t 3.99 min, 79% purity. ESI-TOF spectrum found within R_t 2–8 min (insert: deconvoluted masses). Monoisotopic mass (ESI+) calcd. for $C_{47}H_{70}N_{14}O_9$ 974.5450, found 974.5450. LCMS Gradient A.

9.5 I, V

9.5.1 Standard conditions

The sequence LIV was synthesized on a previously synthesized LYRAG-tag (LYRAG TAG 4, 8.20 mg, 3.4 μmol) using the immobilized base-SPPS standard protocol (Section SI3). Cleavage of the peptidyl resin according to Cleavage Protocol (Section 1.3) afforded the crude peptide (1.8 mg, 85% purity by LCMS [SI Figure 46], 86% purity by UHPLC [SI Figure 47]).

AA	m(AA) [mg]	Immob. base	Flow rate [mL/min]	Base temp. [°C]	Reactor temp. [°C]	Activator	t_{sw} [min]	t_{fw} [min]
L	69.0	DIPA 7	0.6	90	90	HATU	4	2
I	71.6	DIPA 7	0.6	90	90	HATU	4	2
V	69.0	DIPA 7	0.6	90	90	HATU	4	2

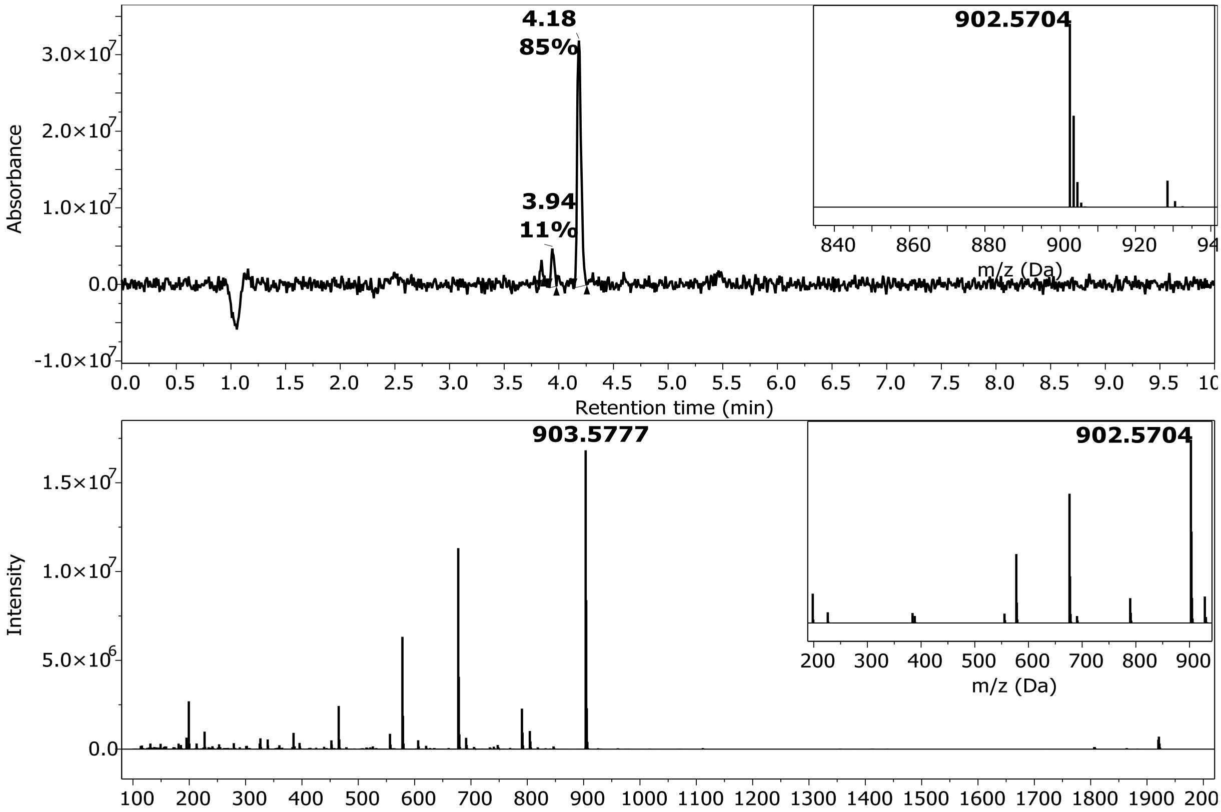

SI Figure 46: LCMS Profile of crude LIVLYRAG. Absorbance chromatogram (λ = 214 nm) of LIVLYRAG; R_t 4.18 min, 85% purity. ESI-TOF spectrum found within R_t 2-8 min (insert: deconvoluted masses). Monoisotopic mass (ESI+) calcd. For $C_{43}H_{74}N_{12}O_9$ 902.5702, found 902.5704. LCMS Gradient A.

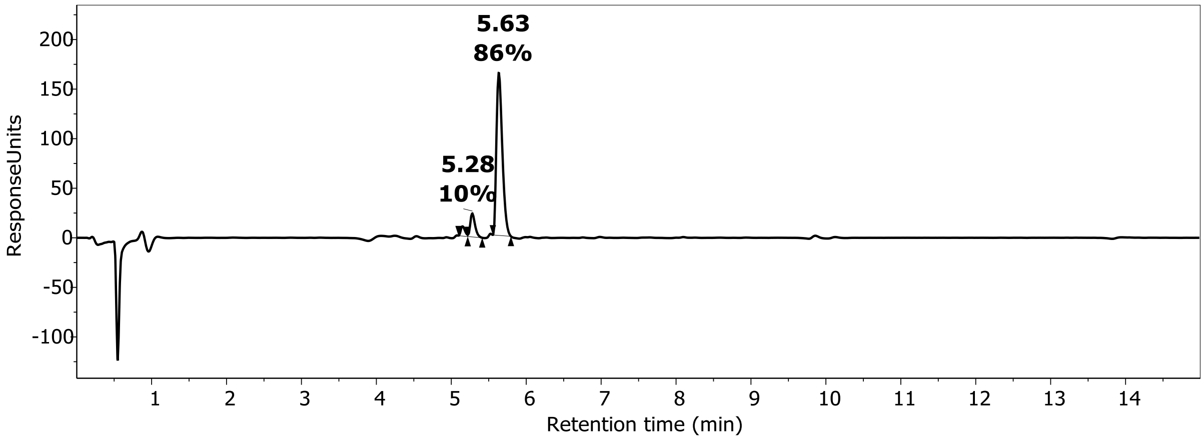

SI Figure 47: UHPLC Profile of crude LIVLYRAG. Absorbance chromatogram (λ = 214 nm) of LIVLYRAG; R_t 5.63 min, 86% purity.

9.5.2 Optimized conditions

The sequence LIV was synthesized on a previously synthesized LYRAG-tag (LYRAG TAG 4, 9.60 mg, 3.9 µmol) using the immobilized base-SPPS standard protocol (Section SI3). Cleavage of the peptidyl resin according to Cleavage Protocol (Section 1.3) afforded the crude peptide (1.8 mg, 100% purity by LCMS [SI Figure 48], 98% purity by UHPLC [SI Figure 49]).

AA	m(AA) [mg]	Immob. base	Flow rate [mL/min]	Base temp. [°C]	Reactor temp. [°C]	Activator	t_{sw} [min]	t_{fw} [min]
L	71.9	DIPA 7	0.4	90	90	HATU	6	2
I	70.5	DIPA 7	0.4	90	90	HATU	6	2
V	66.5	DIPA 7	0.4	90	90	HATU	6	2

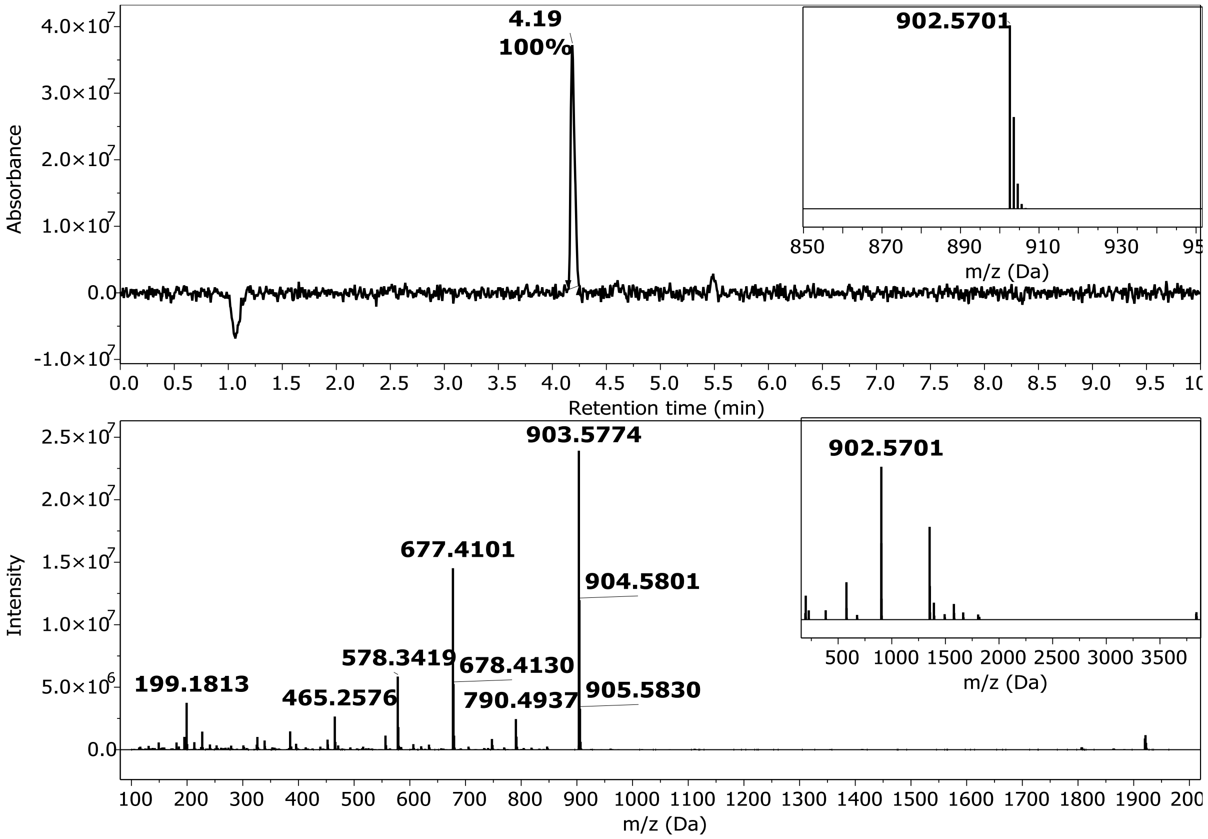

SI Figure 48: LCMS Profile of crude LIVLYRAG. Absorbance chromatogram (λ = 214 nm) of LIVLYRAG; R_t 4.19 min, 100% purity. ESI-TOF spectrum found within R_t 2–8 min (insert: deconvoluted masses). Monoisotopic mass (ESI+) calcd. For $C_{43}H_{74}N_{12}O_9$ 902.5702, found 902.5701. LCMS Gradient A.

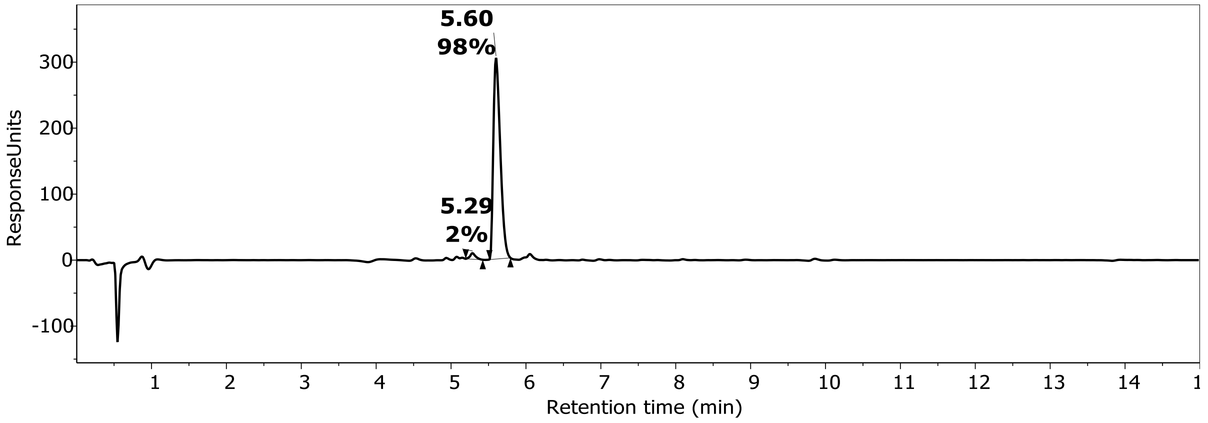

SI Figure 49: UHPLC Profile of crude LIVLYRAG. Absorbance chromatogram (λ = 214 nm) of LIVLYRAG; R_t 5.60 min, 98% purity.

9.6 K, M, N

9.6.1 Standard conditions

The sequence KMN was synthesized on a previously synthesized LYRAG-tag (LYRAG TAG 3, 8.90 mg, 3.6 μmol) using the immobilized base-SPPS standard protocol (Section SI3). Cleavage of the peptidyl resin according to Cleavage Protocol (Section 1.3) afforded the crude peptide (1.8 mg, 93% purity by LCMS [SI Figure 50]).

AA	m(AA) [mg]	Immob. base	Flow rate [mL/min]	Base temp. [°C]	Reactor temp. [°C]	Activator	t_{sw} [min]	t_{fw} [min]
K	94.3	DIPA 5	0.6	90	90	HATU	4	2
M	74.7	DIPA 5	0.6	90	90	HATU	4	2
N	120.4	DIPA 5	0.6	90	90	HATU	4	2

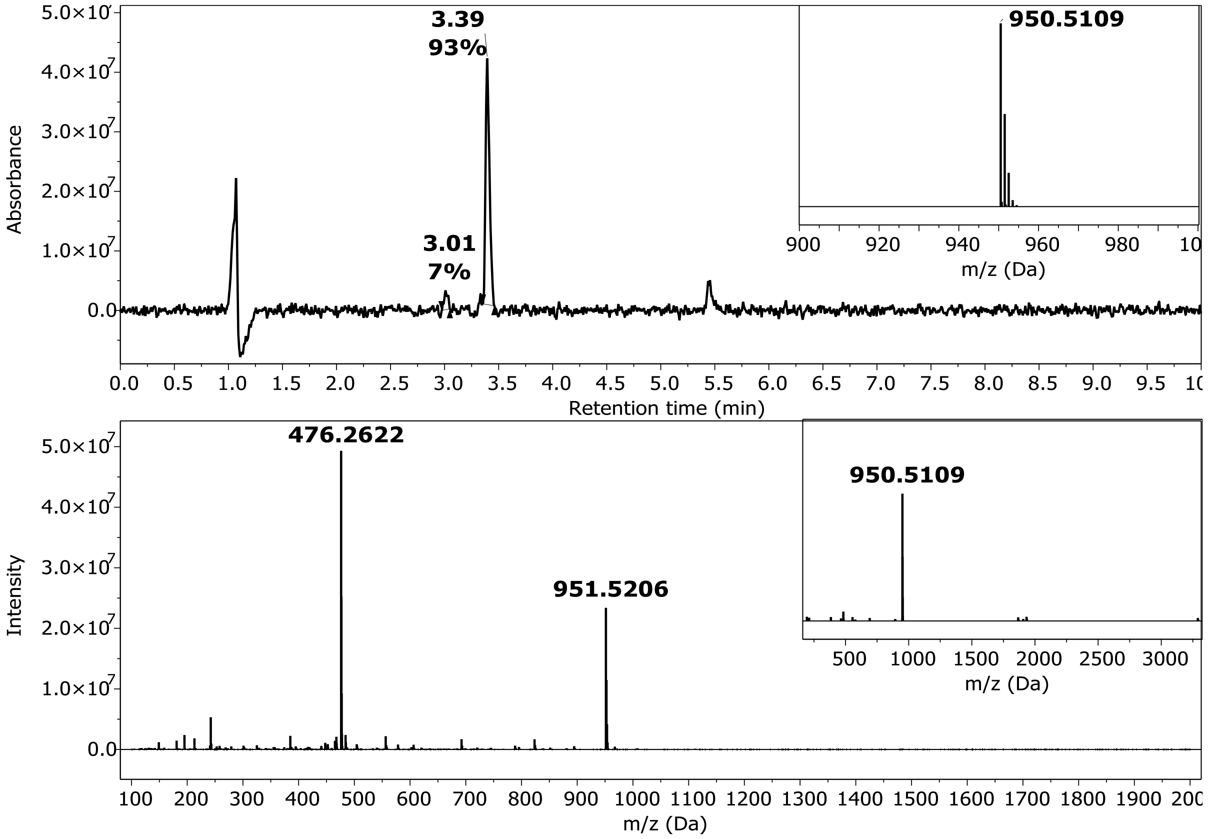

SI Figure 50: LCMS Profile of crude KMNLYRAG. Absorbance chromatogram (λ = 214 nm) of KMNLYRAG; $_{Rt}$ 3.39 min, 93% purity. ESI-TOF spectrum found within $_{Rt}$ 2–8 min (insert: deconvoluted masses). Monoisotopic mass (ESI+) calcd. For $C_{41}H_{70}N_{14}O_{10}S_3$ 950.5120, found 950.5109. LCMS Gradient A.

9.7 P, Q, R, S

9.7.1 Standard conditions

The sequence PQRS was synthesized on a previously synthesized LYRAG-tag (LYRAG TAG 3, 11.6 mg, 4.8 µmol) using the immobilized base-SPPS standard protocol (Section SI3). Cleavage of the peptidyl resin according to Cleavage Protocol (Section 1.3) afforded the crude peptide (1.4 mg, 90% purity by LCMS [SI Figure 51]

AA	m(AA) [mg]	Immob. base	Flow rate [mL/min]	Base temp. [°C]	Reactor temp. [°C]	Activator	t_{sw} [min]	t_{fw} [min]
P	68.3	DIPA 5	0.6	90	90	HATU	4	2
Q	121.2	DIPA 5	0.6	90	90	HATU	4	2
R	130.6	DIPA 5	0.6	90	90	HATU	4	2
S	77.3	DIPA 5	0.6	90	90	HATU	4	2

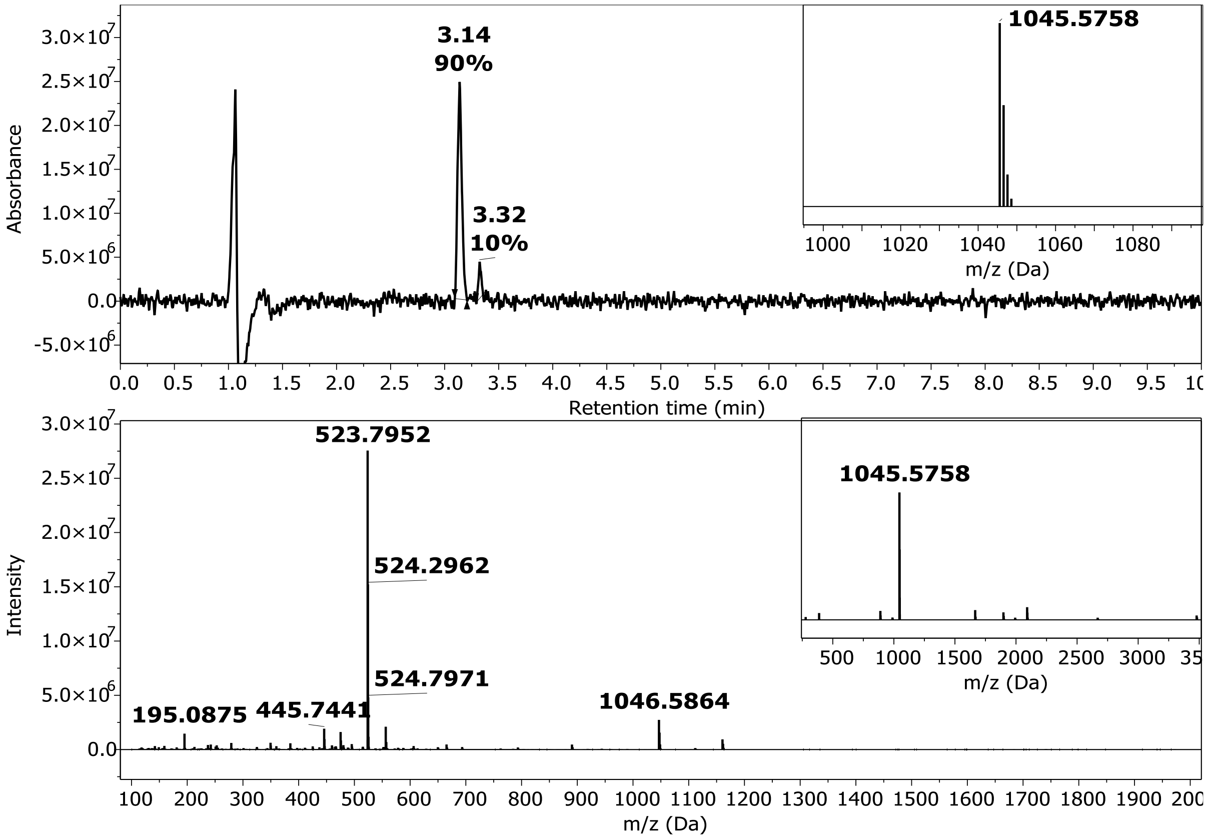

SI Figure 51: LCMS Profile of crude PQRSLYRAG. Absorbance chromatogram (λ = 214 nm) of PQRSLYRAG; R_t 3.14 min, 90% purity. ESI-TOF spectrum found within R_t 2–8 min (insert: deconvoluted masses). Monoisotopic mass (ESI+) calcd. For $C_{45}H_{75}N_{17}O_{12}$ 1045.5781, found 1045.5758. LCMS Gradient A.

9.7.2 Optimized condition for R

The sequence R was synthesized on a previously synthesized LYRAG-tag (LYRAG TAG 3, 11.6 mg, 4.8 μmol) using the immobilized base-SPPS standard protocol (Section SI3). Cleavage of the peptidyl resin according to Cleavage Protocol (Section 1.3) afforded the crude peptide (1.4 mg, 100% purity by LCMS [SI Figure 52]).

AA	m(AA) [mg]	Immob. base	Flow rate [mL/min]	Base temp. [°C]	Reactor temp. [°C]	Activator	t_{sw} [min]	t_{fw} [min]
R		DIPA 5	0.4	90	90	PyAOP	4	2

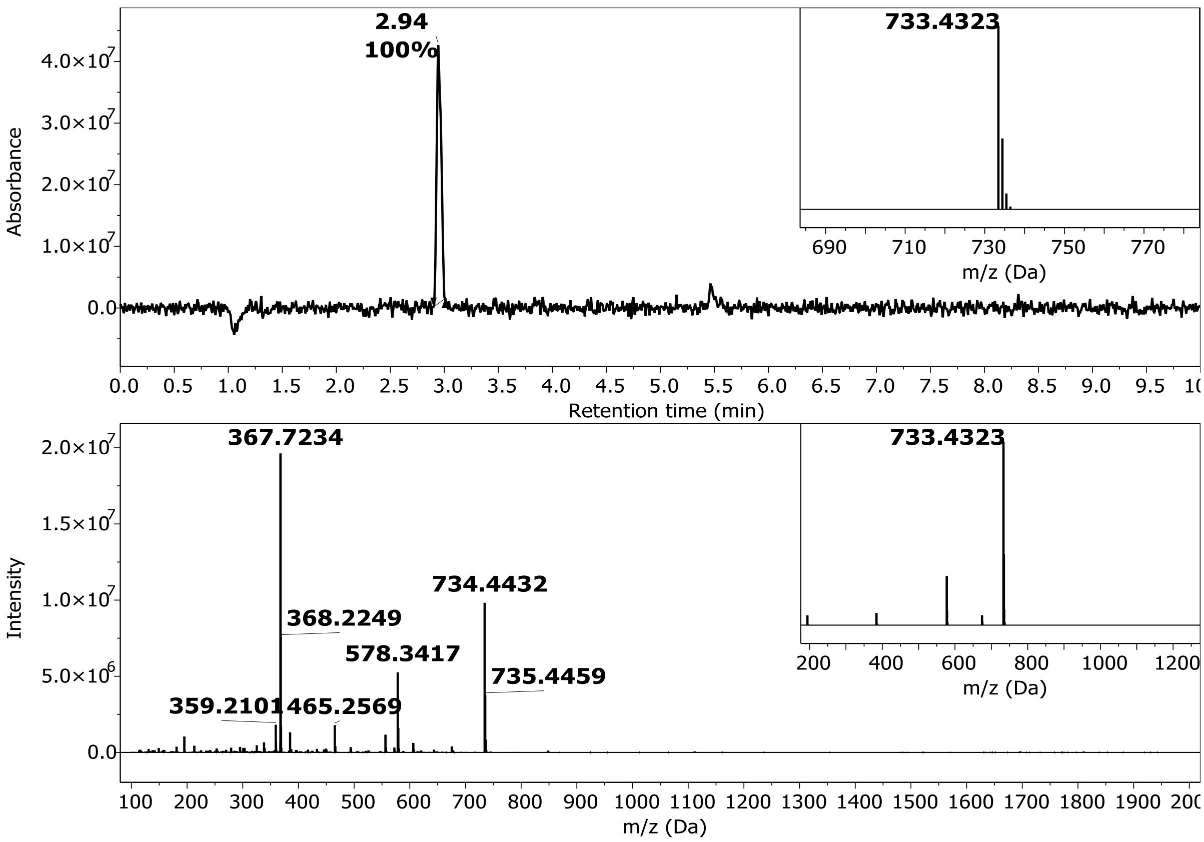

SI Figure 52: LCMS Profile of crude RLYRAG. Absorbance chromatogram (λ = 214 nm) of RLYRAG; R_t 2.94 min, 100% purity. ESI-TOF spectrum found within R_t 2–8 min (insert: deconvoluted masses). Monoisotopic mass (ESI+) calcd. For $C_{32}H_{55}N_{13}O_7$ 733.4347, found 733.4323. LCMS Gradient A.

9.8 T, W, Y

The sequence TVWY was synthesized on a previously synthesized LYRAG-tag (LYRAG TAG 3, 11.6 mg, 4.8 μmol) using the immobilized base-SPPS standard protocol (Section SI3). Cleavage of the peptidyl resin according to Cleavage Protocol (Section 1.3) afforded the crude peptide (2.0 mg, 95% purity by LCMS [SI Figure 53]).

AA	m(AA) [mg]	Immob. base	Flow rate [mL/min]	Base temp. [°C]	Reactor temp. [°C]	Activator	t_{sw} [min]	t_{fw} [min]
T	79.2	DIPA 5	0.6	90	90	HATU	4	2
V	69.8	DIPA 5	0.6	90	90	HATU	4	2
W	106.6	DIPA 5	0.6	90	90	HATU	4	2
Y	91.7	DIPA 5	0.6	90	90	HATU	4	2

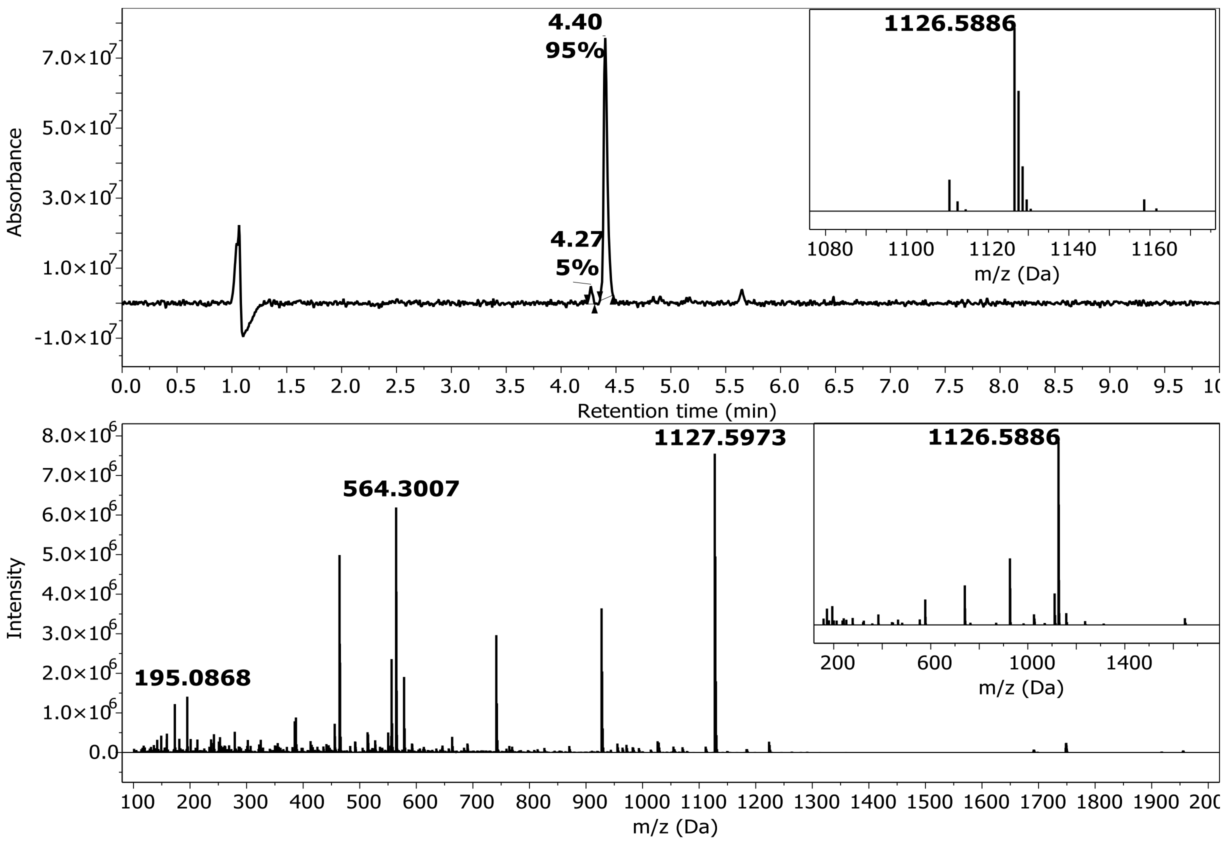

SI Figure 53: LCMS Profile of crude TVWYLYRAG. Absorbance chromatogram (λ = 214 nm) of TVWYLYRAG; R_t 4.40 min, 95% purity. ESI-TOF spectrum found within R_t 2–8 min (insert: deconvoluted masses). Monoisotopic mass (ESI+) calcd. For $C_{55}H_{78}N_{14}O_{12}$ 1126.5924, found 1126.5886. LCMS Gradient A.

10 Piperidine reduction

10.1 0.3 mL piperidine

The sequence ALF was synthesized on a previously synthesized LYRAG-tag (LYRAG TAG 5, 10.2 mg, 4.2 μmol) using an adapted reduced piperidine immobilized base-SPPS standard protocol (Section SI3): The adapted deprotection step is specified in the table below. Cleavage of the peptidyl resin according to Cleavage Protocol (Section 1.3) afforded the crude peptide (2.0 mg, 64 % purity by LCMS [SI Figure 54]).

AA	m(AA) [mg]	Immob. base	Flow rate [mL/min]	Base temp. [°C]	Reactor temp. [°C]	Activator	t_{sw} [min]
A	62.0	DIPA 7	0.6	90	90	HATU	4
L	72.3	DIPA 7	0.6	90	90	HATU	4
F	78.7	DIPA 7	0.6	90	90	HATU	4

Depro.	V(pip, 20%) [mL]	Flow rate [mL/min]	t_{fw1} [min]	t_{fw2} [min]	$t_{fw,final}$ [min]
Pip.	0.3	5	0.6	3.4	t_{fw2}

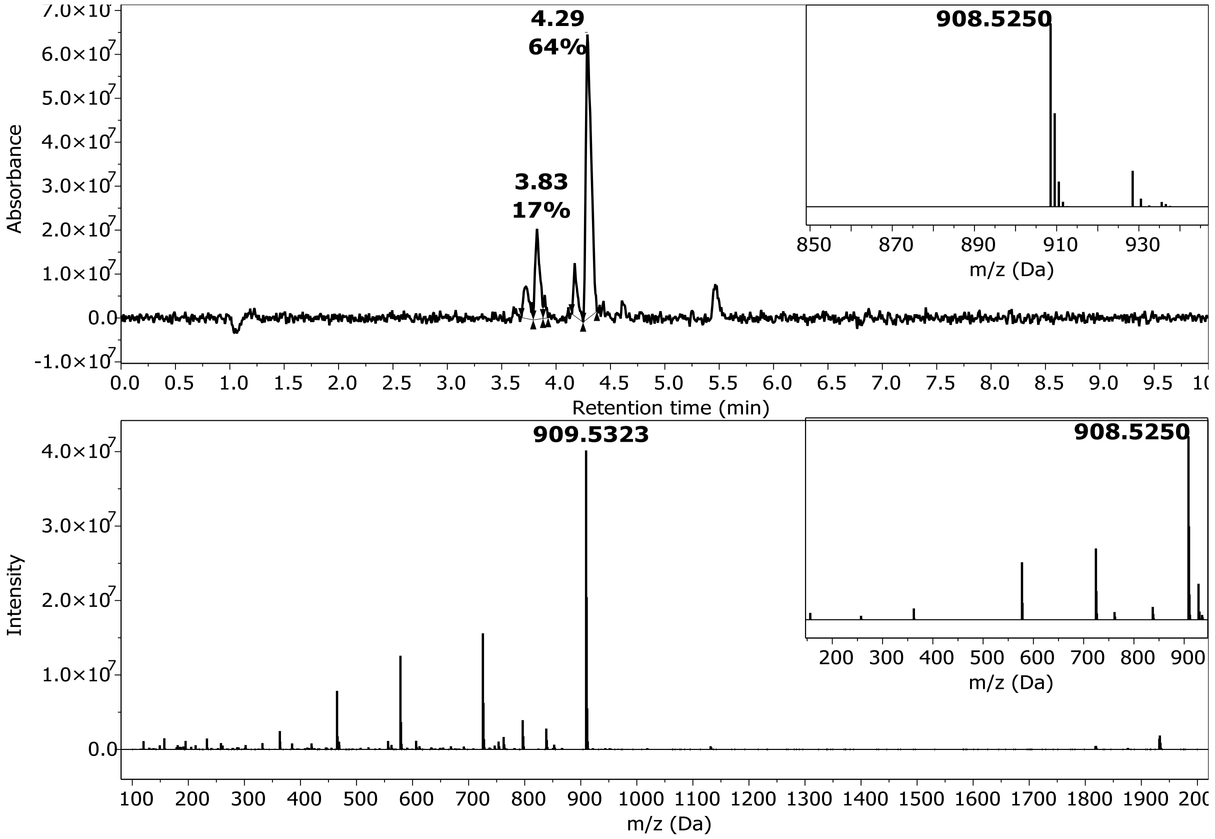

SI Figure 54: LCMS Profile of crude ALFLYRAG. Absorbance chromatogram (λ = 214 nm) of ALFLYRAG; R_t 4.29 min, 64% purity. ESI-TOF spectrum found within R_t 2–8 min (insert: deconvoluted masses). Monoisotopic mass (ESI+) calcd. for $C_{44}H_{68}N_{12}O_9$ 908.5232, found 908.5250. LCMS Gradient A.

10.2 0.6 mL piperidine

The sequence ALF was synthesized on a previously synthesized LYRAG-tag (LYRAG TAG 5, 10.2 mg, 4.2 μmol) using an adapted reduced piperidine immobilized base-SPPS standard protocol (Section SI3): The adapted deprotection step is specified in the table below. Cleavage of the peptidyl resin according to Cleavage Protocol (Section 1.3) afforded the crude peptide (3.8 mg, 95% purity by LCMS [SI Figure 55]).

AA	m(AA) [mg]	Immob. base	Flow rate [mL/min]	Base temp. [°C]	Reactor temp. [°C]	Activator	t_{sw} [min]
A	64.3	DIPA 7	0.6	90	90	HATU	4
L	71.8	DIPA 7	0.6	90	90	HATU	4
F	77.1	DIPA 7	0.6	90	90	HATU	4

Depro.	V(pip, 20%) [mL]	Flow rate [mL/min]	t_{fw1} [min]	t_{fw2} [min]	$t_{fw,final}$ [min]
Pip.	0.6	5	0.6	3.4	t_{fw2}

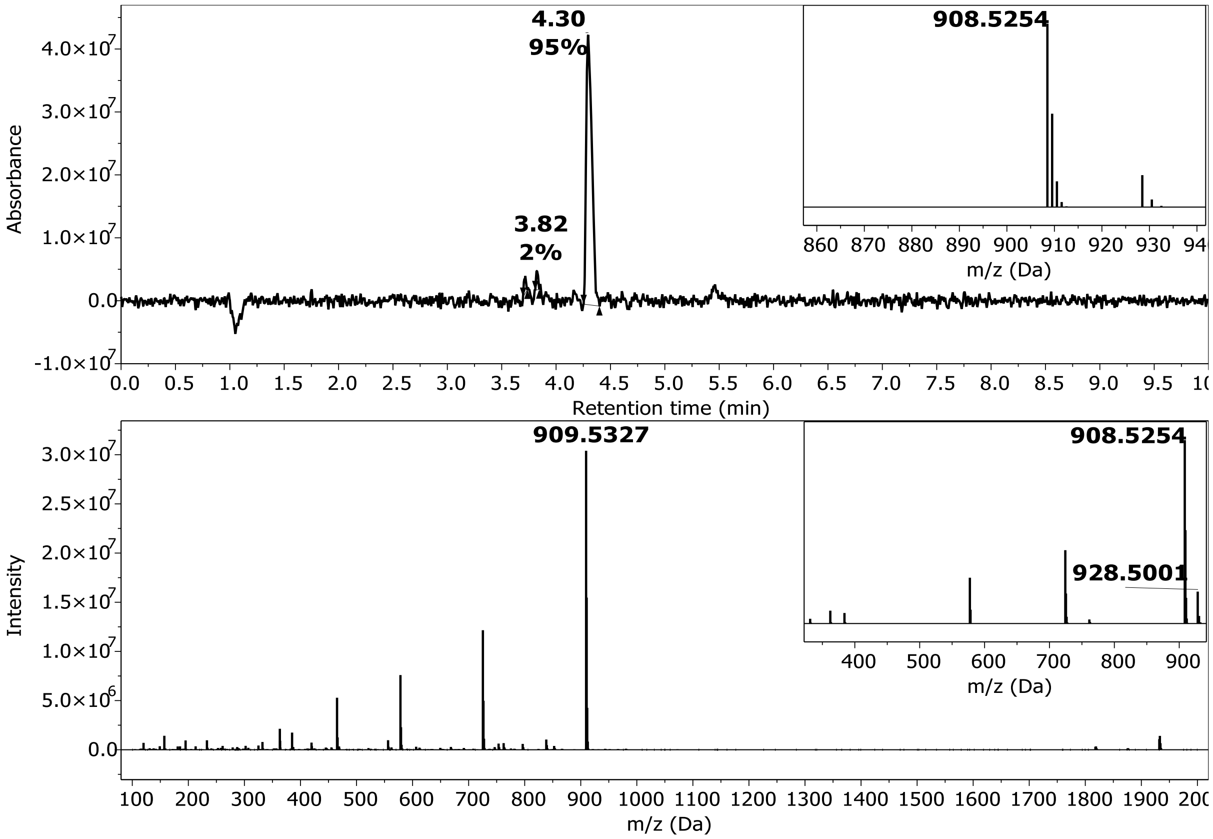

SI Figure 55: LCMS Profile of crude ALFLYRAG. Absorbance chromatogram (λ = 214 nm) of ALFLYRAG; R_t 4.30 min, 95% purity. ESI-TOF spectrum found within R_t 2–8 min (insert: deconvoluted masses). Monoisotopic mass (ESI+) calcd. for $C_{44}H_{68}N_{12}O_9$ 908.5232, found 908.525. LCMS Gradient A.

11 Synthesis of peptides using the DIPA-functionalized base as catalyst

11.1 LYRAG

11.1.1 Synthesis with the optimized conditions for each individual amino acid

The sequence LYRAG was synthesized on commercially available Novabiochem® NovaPEG Rink Amide resin (0.41 mmol/g, 9.6 mg, 3.90 µmol) using the immobilized base-SPPS standard protocol (Section SI3). Cleavage of the peptidyl resin according to Cleavage Protocol (Section 1.3) afforded the crude peptide (63% purity by LCMS [SI Figure 56]).

AA	m(AA) [mg]	Immob. base	Equiv.	Flow rate [mL/min]	Base temp. [°C]	Reactor temp. [°C]	Activator	t_{sw} [min]	t_{fw} [min]
L	71.6	DIPA 7	1	0.5	90	90	HATU	5	2
Y	92.4	DIPA 7	1	0.6	90	90	HATU	4	2
R	260.1	DIPA 7	2*	0.6	90	90	PyAOP	4	2
A	63.8	DIPA 7	1	0.6	90	90	HATU	4	2
G	60.3	DIPA7	1	0.4	90	90	HATU	6	2

*0.4 M, 1.0 mL

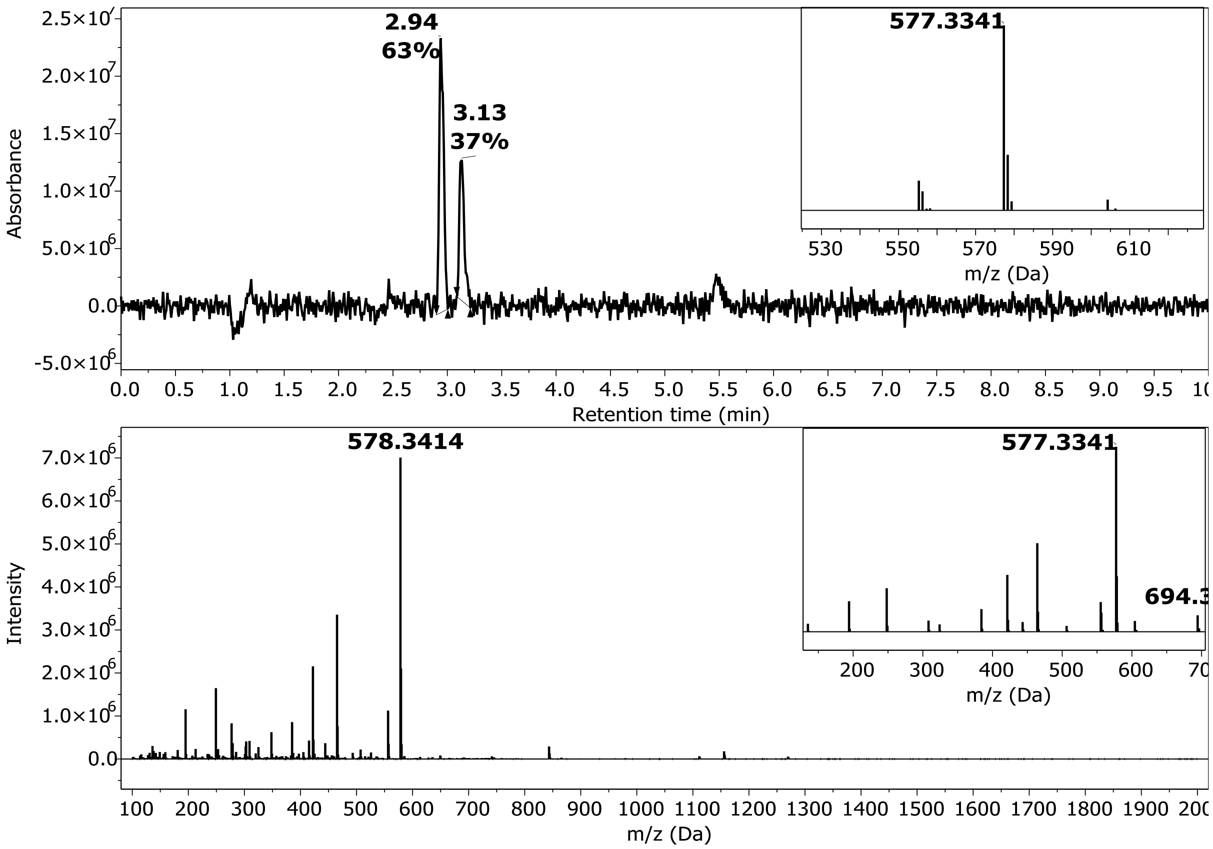

SI Figure 56: LCMS Profile of crude LYRAG. Absorbance chromatogram (λ = 214 nm) of LYRAG; $_{Rt}$ 2.94 min, 63% purity. ESI-TOF spectrum found within $_{Rt}$ 2–8 min (insert: deconvoluted masses). Monoisotopic mass (ESI+) calcd. for $C_{26}H_{43}N_9O_6$ 577.3336 found 577.3341. LCMS Gradient A.

11.1.2 Synthesis with double addition of R

The sequence LYRAG was synthesized on commercially available Novabiochem® NovaPEG Rink Amide resin (0.41 mmol/g, 9.90 mg, 4.1 µmol) using the immobilized base-SPPS, with no deprotection between the arginine additions to minimize double coupling. Cleavage of the peptidyl resin according to Cleavage Protocol (Section 1.3) afforded the crude peptide (85% purity by LCMS [SI Figure 57]).

AA	m(AA) [mg]	Immob. base	Equiv.	Flow rate [mL/min]	Base temp. [°C]	Reactor temp. [°C]	Activator	t_{sw} [min]	t_{fw1} [min]	t_{fw2} [min]
L	71.7	DIPA 7	1	0.5	90	90	HATU	5	-	2
Y	91.1	DIPA 7	1	0.6	90	90	HATU	4	-	2
R	130.45	DIPA 7	1*	0.6	90	90	PyAOP	4	2	2
R	130.45	DIPA 7	1*	0.6	90	90	PyAOP	2	2	3*
A	62.2	DIPA 7	1	0.6	90	90	HATU	4	-	2
G	59.6	DIPA7	1	0.5	90	90	HATU	5	-	2

*2 x 0.4 M, 0.5 mL with base regeneration, but no deprotection

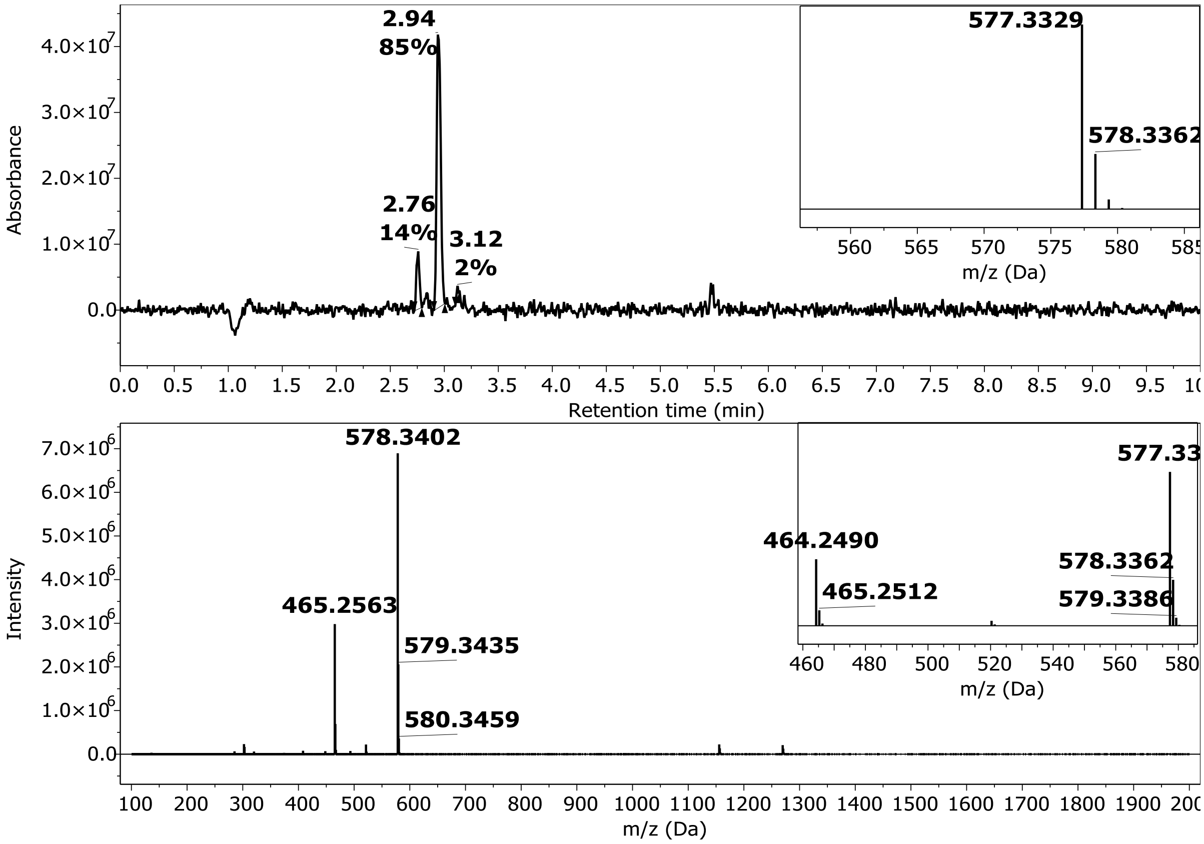

SI Figure 57: LCMS Profile of crude LYRAG. Absorbance chromatogram (λ = 214 nm) of LYRAG; R_t 2.94 min, 85% purity. ESI-TOF spectrum found within R_t 2–8 min (insert: deconvoluted masses). Monoisotopic mass (ESI+) calcd. for $C_{26}H_{43}N_9O_6$577.3336 found 577.3334. LCMS Gradient A.

11.2 NBDY [53–68]

11.2.1 Synthesis with the optimized conditions for each individual amino acid

The sequence PPASAGLKSHPPPPPEK was synthesized on commercially available Novabiochem® NovaPEG Rink Amide resin (0.41 mmol/g, 10.4 mg, 4.3 µmol) using the reduced piperidine immobilized base-SPPS standard protocol (Section SI3). Cleavage of the peptidyl resin according to Cleavage Protocol (Section 1.3) afforded the crude peptide (68% purity by LCMS [SI Figure 58], 66% purity by UHPLC [SI Figure 59]).

AA	m(AA) [mg]	Immob. base	Flow rate [mL/min]	Base temp. [°C]	Reactor temp. [°C]	Activator	t_{sw} [min]
6 x P	403.5	DIPA 8	0.6	90	90	HATU	4
2 x A	132.1	DIPA 8	0.6	90	90	HATU	4
2 x S	155.4	DIPA 8	0.4	90	90	HATU	8
K[60]	93.3	DIPA 8	0.6	90	90	HATU	4
K[68]	93.3	DIPA 8	0.4	90	90	HATU	8
G	60.2	DIPA 8	0.6	90	90	HATU	4
L	71.3	DIPA 8	0.6	90	90	HATU	4
H(Boc)	94.0	DIPA 8	0.6	90	90	HATU	4
E	90.4	DIPA 8	0.4	90	90	HATU	8
Depro.	V(pip, 20%) [mL]	Flow rate [mL/min]	t_{fw1} [min]	t_{fw2} [min]	$t_{fw,final}$ [min]		
Pip.	0.6	5	0.6	3.4	t_{fw2}		

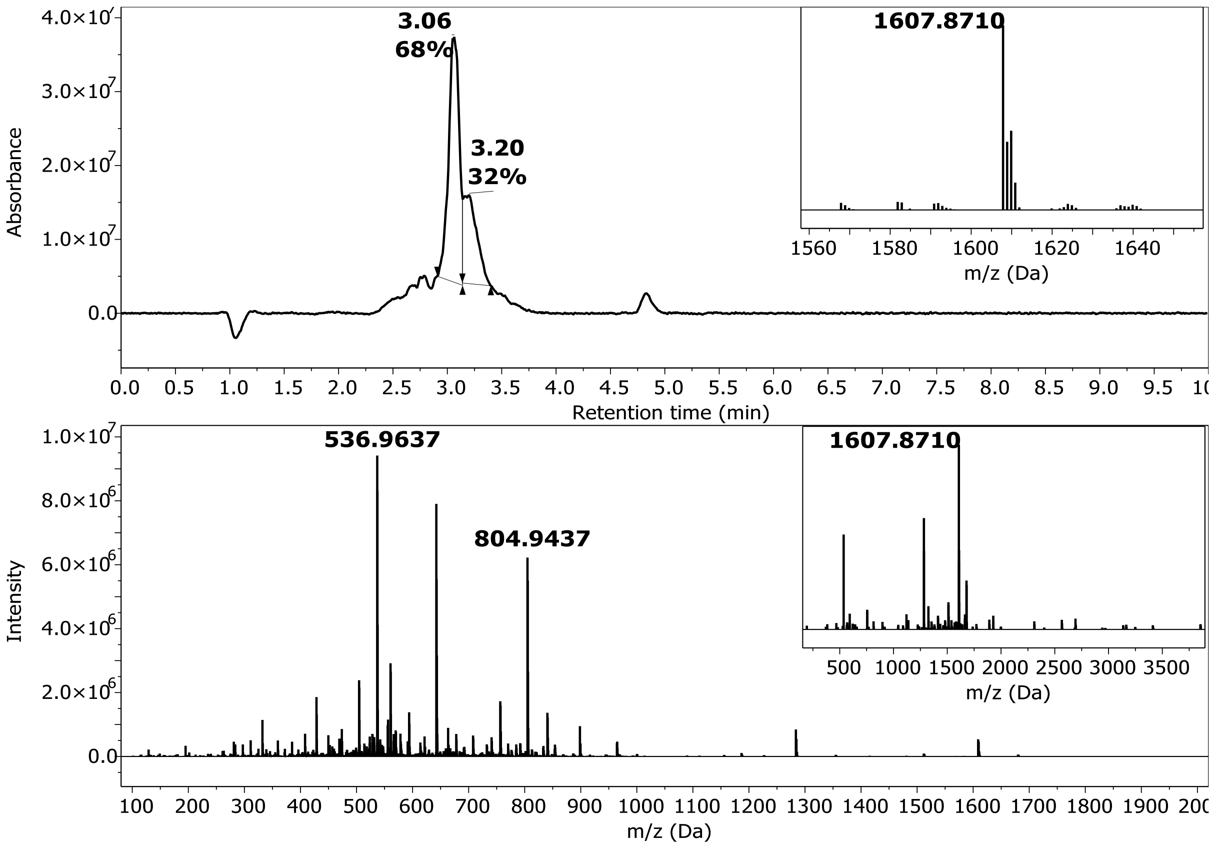

SI Figure 58: LCMS Profile of crude PPASAGLKSHPPPPEK. Absorbance chromatogram (λ = 214 nm) of PPASAGLKSHPPPPEK; R_t 3.06 min, 68% purity. ESI-TOF spectrum found within R_t 2–8 min (insert: deconvoluted masses). Monoisotopic mass (ESI+) calcd. for C73H117N21O20 1607.8784, found 1607.8710. LCMS gradient A.

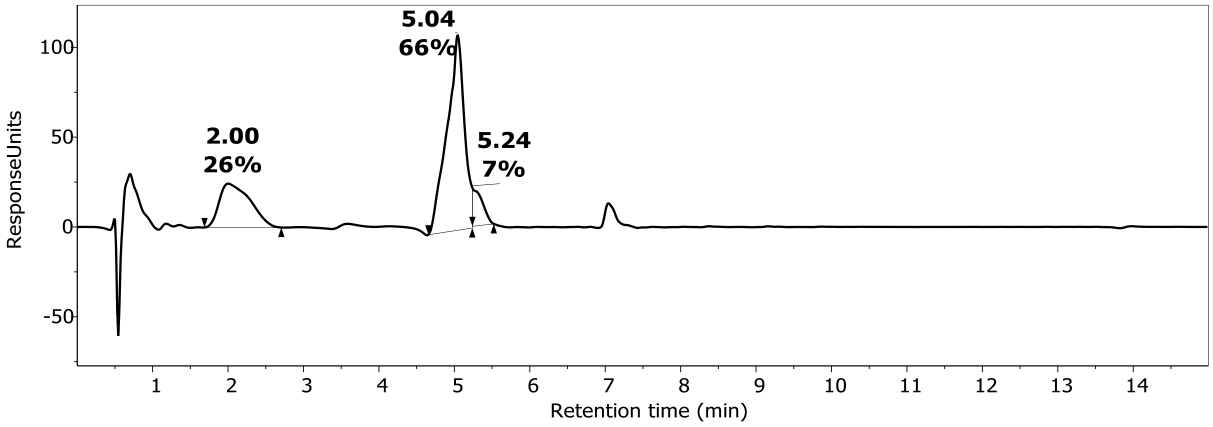

SI Figure 59: UHPLC profile of crude NBDY[53–68]. R_t 5.04 min. 66% purity based on Area Under Curve (AUC) at λ = 214 nm.

11.3 HIV-1 protease [88–99]

11.3.1 Synthesis with the conditions optimized for each individual amino acid

The sequence NLLTQLGCTLNF was synthesized on commercially available Novabiochem® NovaPEG Rink Amide resin (0.41 mmol/g, 10.8 mg, 4.43 μmol) using the reduced piperidine immobilized base-SPPS standard protocol (Section SI3). Cleavage of the peptidyl resin according to Cleavage Protocol (Section 1.3) afforded the crude peptide (1.5 mg, 50% purity by LCMS [SI Figure 60], 64% purity by UHPLC [SI Figure 61]).

AA	m(AA) [mg]	Immob. base	Flow rate [mL/min]	Base temp. [°C]	Reactor temp. [°C]	Activator	t_{sw} [min]
N[1]	120.4	DIPA 11	0.6	90	90	HATU	4
N[11]	120.5	DIPA 11	0.6	90	90	HATU	4
L[2]	71.7	DIPA 11	0.4	90	90	HATU	8
L[3]	71.8	DIPA 11	0.6	90	90	HATU	4
L[6]	71.0	DIPA 11	0.6	90	90	HATU	4
L[10]	71.6	DIPA 11	0.6	90	90	HATU	4
T[4]	79.5	DIPA 11	0.6	90	90	HATU	4
T[9]	80.8	DIPA 11	0.6	90	90	HATU	4
Q	122.5	DIPA 11	0.6	90	90	HATU	4
G	61.0	DIPA 11	0.6	90	90	HATU	4
C	117.9	DIPA 11	0.6	90	90	HATU	4
F	77.4	DIPA 11	0.6	90	90	HATU	4
Depro.	V(pip, 20%) [mL]	Flow Rate [mL/min]	t_{fw1} [min]	t_{fw2} [min]	$t_{fw,final}$ [min]		
Pip.	1.0	5	0.6	3.4	t_{fw2}		

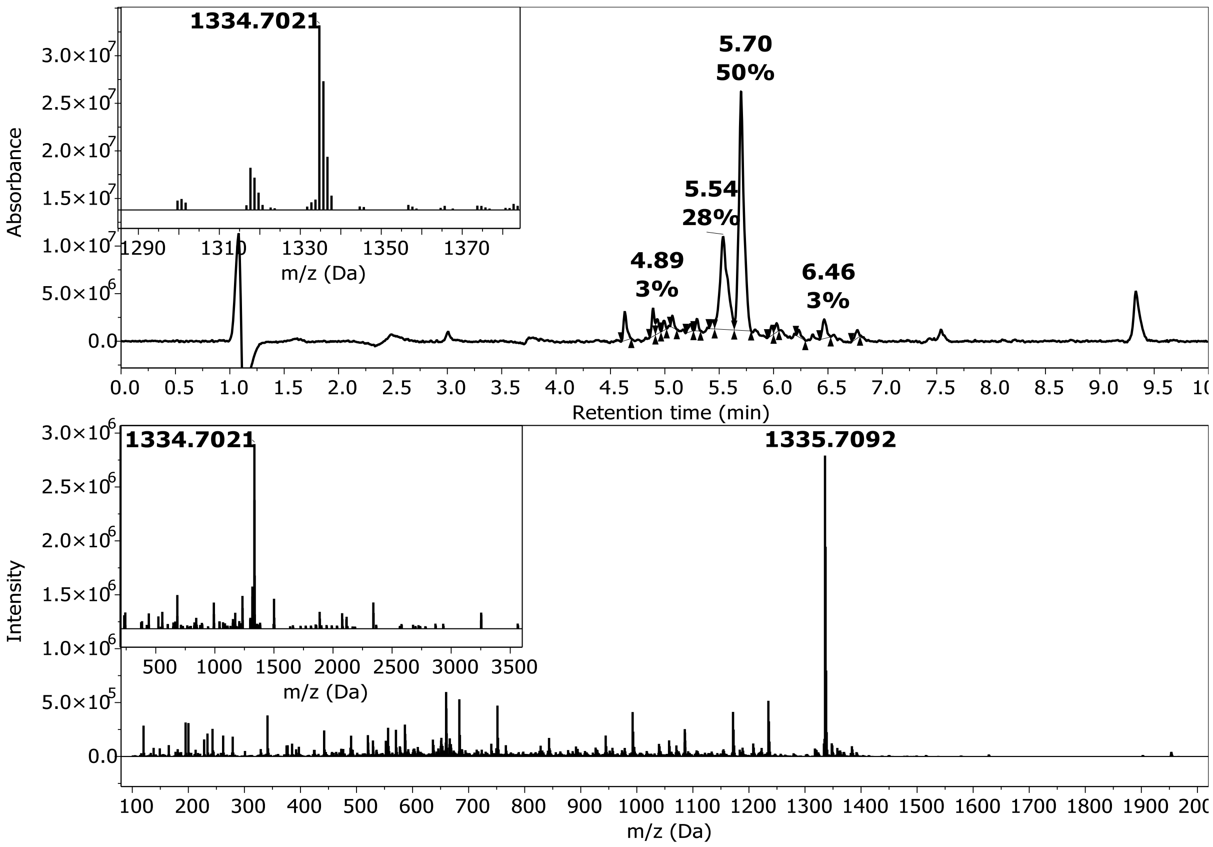

SI Figure 60: LCMS Profile of crude NLLTQLGCTLNF. Absorbance chromatogram (λ = 214 nm) of NLLTQLGCTLNF; R_t 5.70 min, 50% purity. ESI-TOF spectrum found within $_{Rt}$ 2–8 min (insert: deconvoluted masses). Monoisotopic mass (ESI+) calcd. for $C_{59}H_{98}N_{16}O_{17}S$ 1334.7017, found 1334.7021. LCMS gradient A.

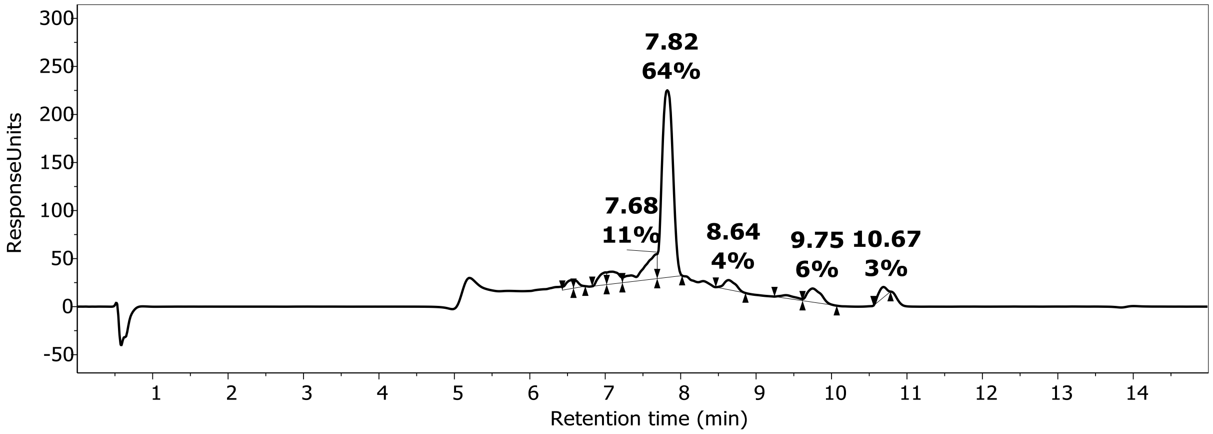

SI Figure 61: UHPLC profile of crude. HIV1-Protease[88—99]. R_t 7.82 min. 64% purity based on Area Under Curve (AUC) at λ = 214 nm.

11.3.2 110°C reactor temperature for all amino acids

The sequence NLLTQLGCTLNF was synthesized on commercially available Novabiochem® NovaPEG Rink Amide resin (0.41 mmol/g, 10.9 mg, 4.47 μmol) using the reduced piperidine immobilized base-SPPS standard protocol (Section SI3). Cleavage of the peptidyl resin according to Cleavage Protocol (Section 1.3)

afforded the crude peptide (3.1 mg, 58% purity by LCMS [SI Figure 62], 61% purity by UHPLC [SI Figure 63]).

AA	m(AA) [mg]	Immob. base	Flow rate [mL/min]	Base temp. [°C]	Reactor temp. [°C]	Activator	t_{sw} [min]
N[1]	120.2	DIPA 11	0.6	90	110	HATU	4
N[11]	119.4	DIPA 11	0.6	90	110	HATU	4
L[2]	70.5	DIPA 11	0.4	90	110	HATU	8
L[3]	71.8	DIPA 11	0.6	90	110	HATU	4
L[6]	71.7	DIPA 11	0.6	90	110	HATU	4
L[10]	70.7	DIPA 11	0.6	90	110	HATU	4
T[4]	80.5	DIPA 11	0.6	90	110	HATU	4
T[9]	81.0	DIPA 11	0.6	90	110	HATU	4
Q	122.4	DIPA 11	0.6	90	110	HATU	4
G	60.2	DIPA 11	0.6	90	110	HATU	4
C	117.7	DIPA 11	0.6	90	110	HATU	4
F	79.2	DIPA 11	0.6	90	110	HATU	4
Depro.	V(pip, 20%) [mL]	Flow Rate [mL/min]	t_{fw1} [min]	t_{fw2} [min]	$t_{fw,final}$ [min]		
Pip.	1.0	5	0.6	3.4	t_{fw2}		

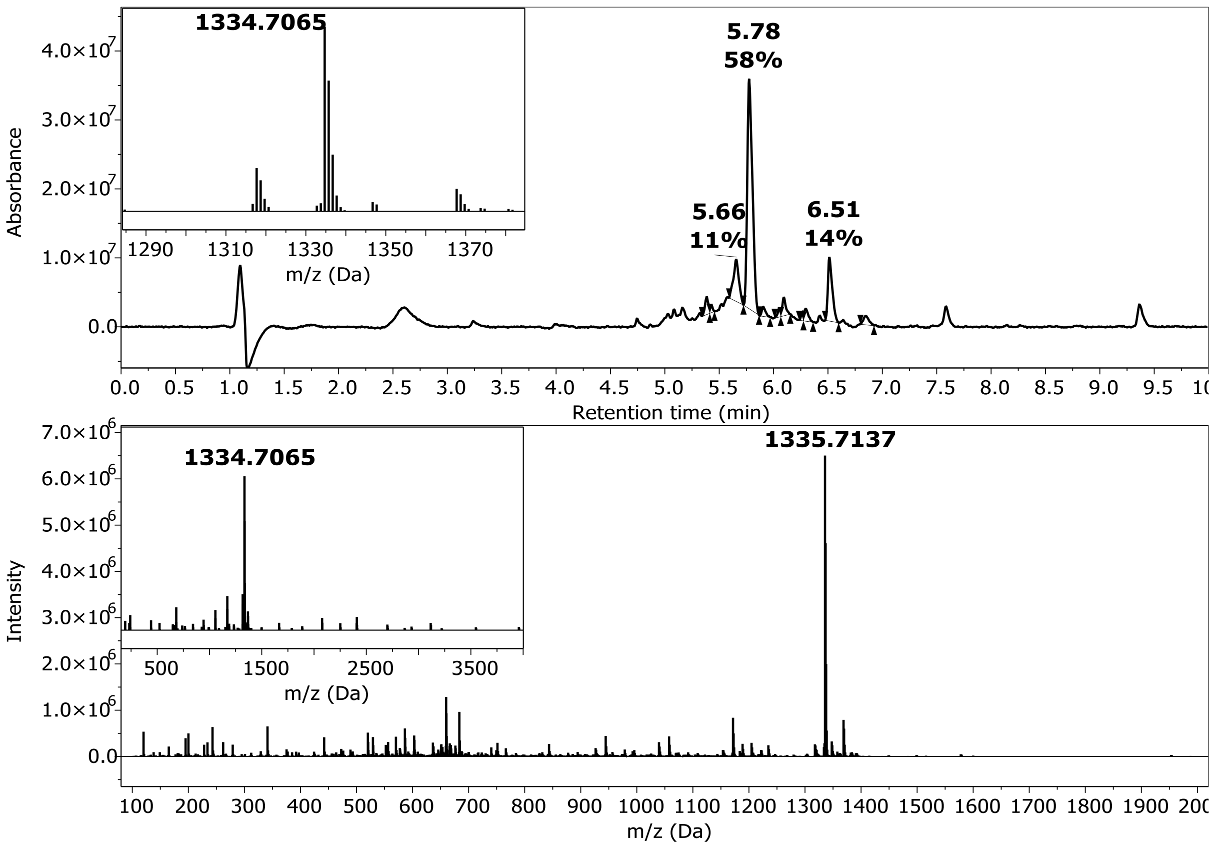

SI Figure 62: LCMS Profile of crude NLLTQLGCTLNF. Absorbance chromatogram (λ = 214 nm) of NLLTQLGCTLNF; R_t 5.78 min, 58% purity. ESI-TOF spectrum found within $_{Rt}$ 2–8 min (insert: deconvoluted masses). Monoisotopic mass (ESI+) calcd. for $C_{59}H_{98}N_{16}O_{17}S$ 1334.7017, found 1334.7065. LCMS gradient A

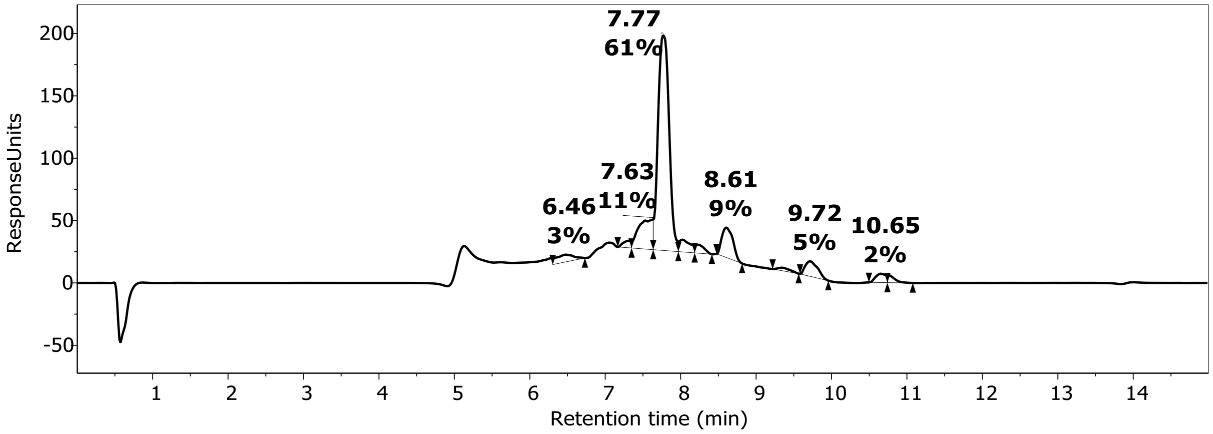

SI Figure 63: UHPLC profile of crude. HIV1-Protease[88—99]. R_t 7.77 min. 61% purity based on Area Under Curve (AUC) at λ = 214 nm.

12 Test of the effect of temperature on epimerization sensitive amino acids cysteine and histidine

12.1 GCF added to LYRAG tag at 100 °C

The sequence GCF was synthesized on a previously synthesized LYRAG-tag (LYRAG TAG 6, 9.9 mg, 4.1 μmol) using the immobilized base-SPPS standard protocol (Section SI3). Cleavage of the peptidyl resin according to Cleavage Protocol (Section 1.3) afforded the crude peptide (2.5 mg, 94% purity by LCMS [SI Figure 64], 99% purity by UHPLC [SI Figure 65]).

AA	m(AA) [mg]	Immob. base	Flow rate [mL/min]	Base temp. [°C]	Reactor temp. [°C]	Activator	t_{sw} [min]	t_{fw} [min]
G	60.0	DIPA 8	0.6	90	100	HATU	4	2
C	116.4	DIPA 8	0.6	90	100	HATU	4	2
F	78.1	DIPA 8	0.6	90	100	HATU	4	2
L:D	80:1							

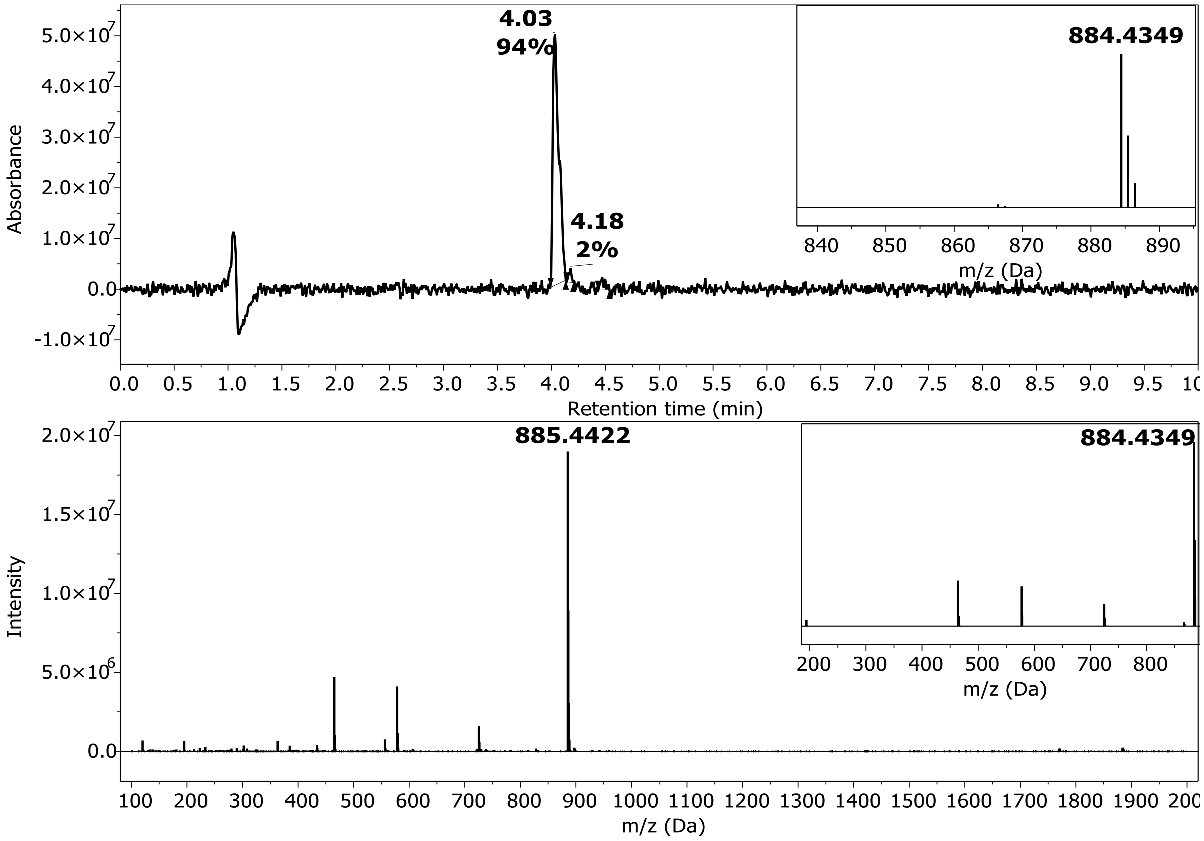

SI Figure 64: LCMS Profile of crude GCFLYRAG. Absorbance chromatogram (λ = 214 nm) of GCFLYRAG; R_t 4.03 min, 94% purity. ESI-TOF spectrum found within R_t 2–8 min (insert: deconvoluted masses). Monoisotopic mass (ESI+) calcd. for $C_{40}H_{60}N_{12}O_9S$ 884.4327, found 884.4349. LCMS Gradient A.

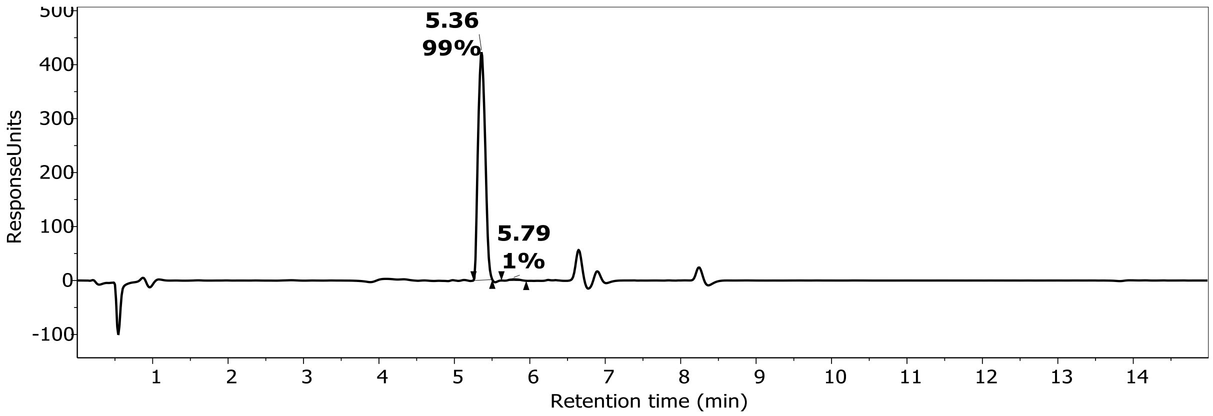

SI Figure 65: UHPLC Profile of crude GCFLYRAG. Absorbance chromatogram (λ = 214 nm) of GCFLYRAG; R_t 5.36 min, 99% purity.

12.2 GCF added to LYRAG tag at 110 °C

The sequence GCF was synthesized on a previously synthesized LYRAG-tag (LYRAG TAG 6, 10.0 mg, 4.1 µmol) using the immobilized base-SPPS standard protocol (Section SI3). Cleavage of the peptidyl resin according to Cleavage Protocol (Section 1.3) afforded the crude peptide (2.5 mg, 95% purity by LCMS [SI Figure 66], 100% purity by UHPLC [SI Figure 67]).

AA	m(AA) [mg]	Immob. base	Flow rate [mL/min]	Base temp. [°C]	Reactor temp. [°C]	Activator	t_{sw} [min]	t_{fw} [min]
G	60.1	DIPA 8	0.6	90	110	HATU	4	2
C	117.0	DIPA 8	0.6	90	110	HATU	4	2
F	78.0	DIPA 8	0.6	90	110	HATU	4	2
L:D	41:1							

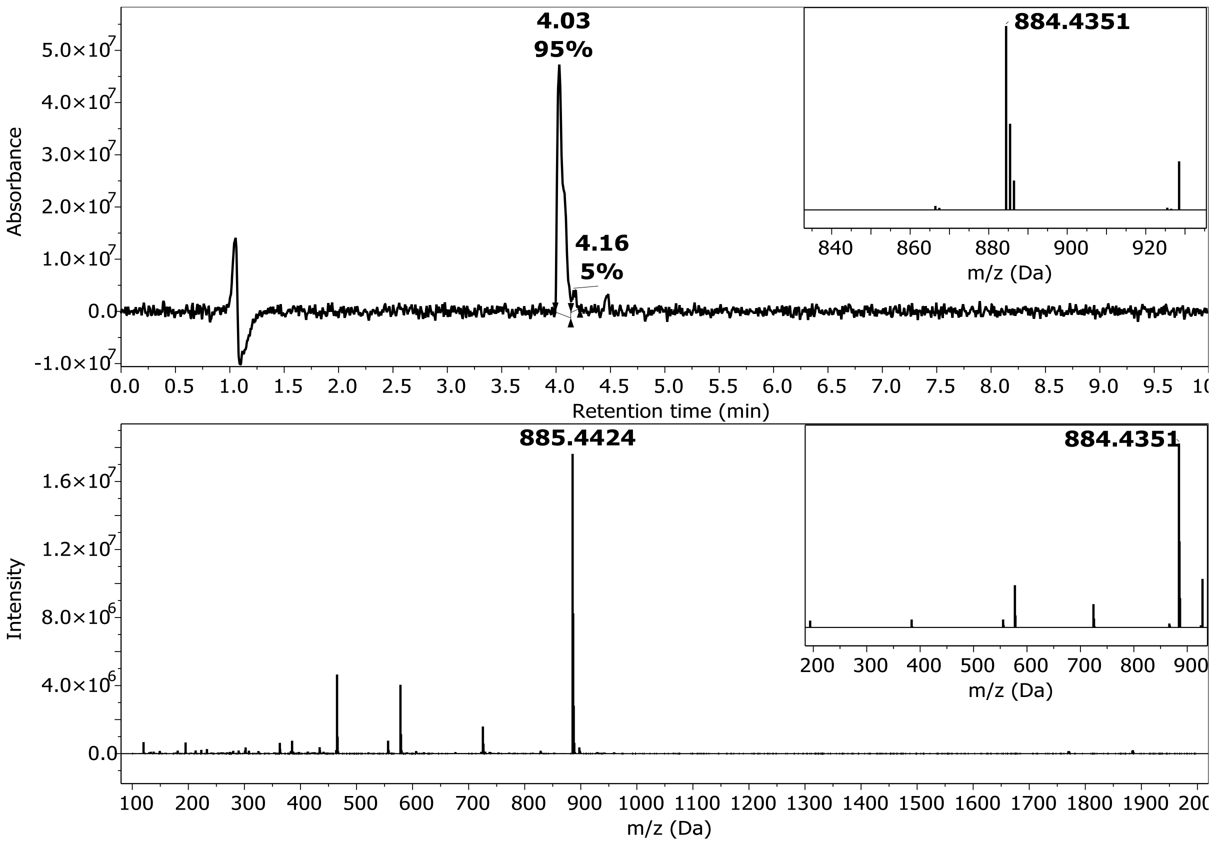

SI Figure 66: LCMS Profile of crude GCFLYRAG. Absorbance chromatogram (λ = 214 nm) of GCFLYRAG; R_t 4.03 min, 95% purity. ESI-TOF spectrum found within R_t 2–8 min (insert: deconvoluted masses). Monoisotopic mass (ESI+) calcd. for $C_{40}H_{60}N_{12}O_9S$ 884.4327, found 884.4351. LCMS Gradient A.

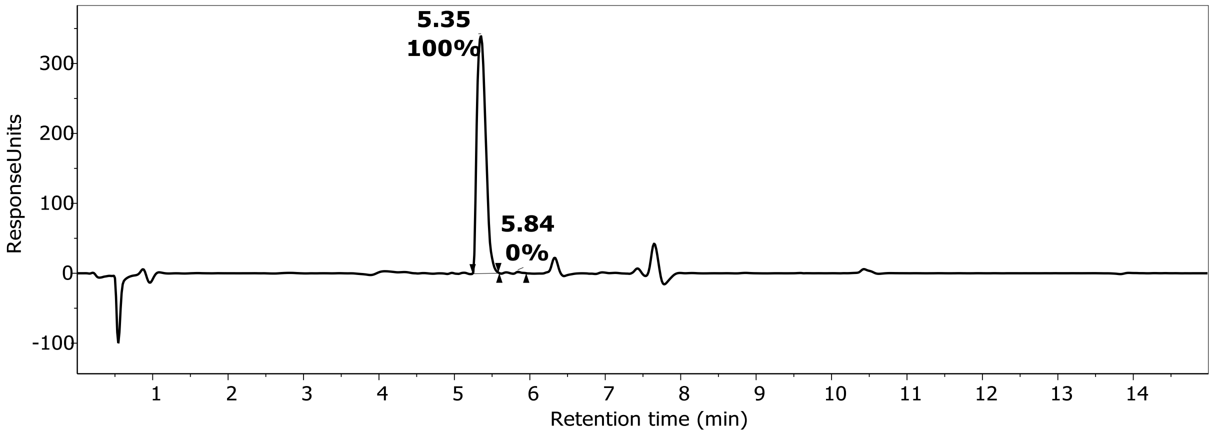

SI Figure 67: UHPLC Profile of crude GCFLYRAG. Absorbance chromatogram (λ = 214 nm) of GCFLYRAG; R_t 5.35 min, 100% purity.

12.3 FHL added to LYRAG tag at 110 °C

The sequence FH(Boc)L was synthesized on a previously synthesized LYRAG-tag (LYRAG TAG 5, 10.6 mg, 3.9 µmol) using the immobilized base-SPPS standard protocol (Section SI3). Cleavage of the peptidyl resin according to Cleavage Protocol (Section 1.3) afforded the crude peptide (1.3 mg, 86% purity by LCMS [SI Figure 68], 85% purity by UHPLC [SI Figure 69]).

AA	m(AA) [mg]	Immob. base	Flow rate [mL/min]	Base temp. [°C]	Reactor temp. [°C]	Activator	t_{sw} [min]	t_{fw} [min]
F	77.9	DIPA 11	0.6	90	110	HATU	4	2
H	96.6	DIPA 11	0.6	90	110	HATU	4	4
L	70.2	DIPA 11	0.4	90	110	HATU	8	4
L:D	14.3:1							

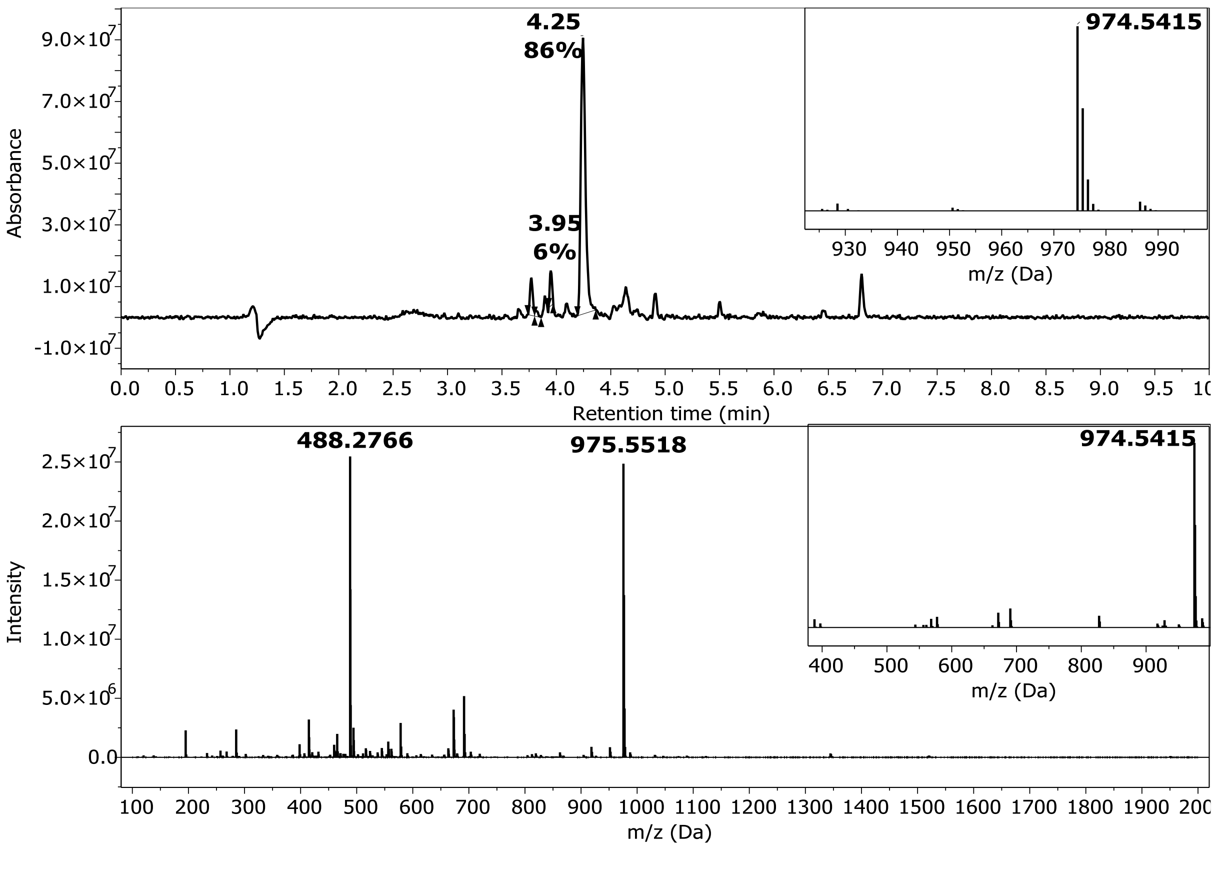

SI Figure 68: LCMS Profile of crude FHLLYRAG. Absorbance chromatogram (λ = 214 nm) of FHLLYRAG; R_t 4.25 min, 86% purity. ESI-TOF spectrum found within R_t 2–8 min (insert: deconvoluted masses). Monoisotopic mass (ESI+) calcd. for $C_{47}H_{70}N_{14}O_9$ 974.5450, found 974.5415. LCMS Gradient A.

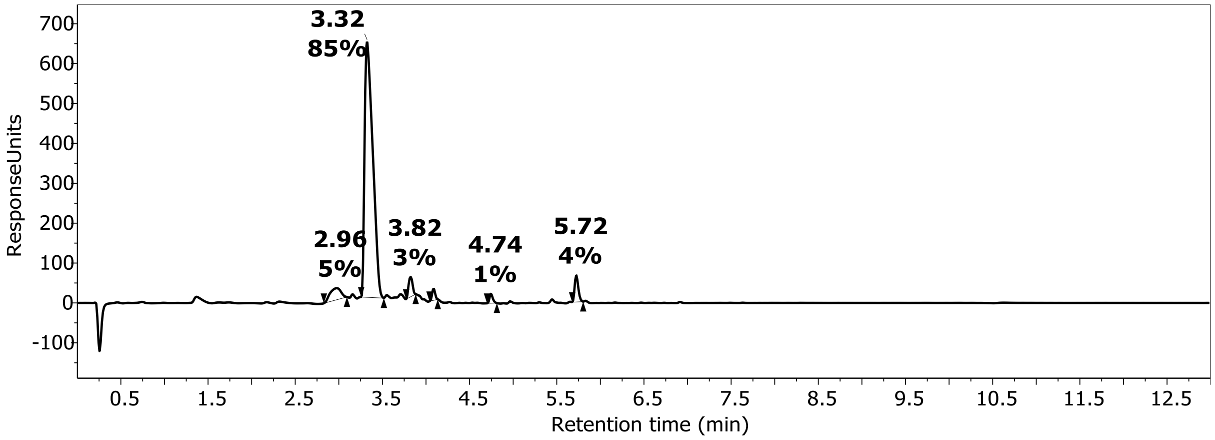

SI Figure 69: UHPLC Profile of crude FHLLYRAG. Absorbance chromatogram (λ = 214 nm) of FHLLYRAG; R_t 3.32 min, 85% purity.

13 Synthesis of aspartimide formation prone sequence NN92[2–29]

NN92 as a test sequence known from literature was selected for investigating aspartimide formation. This sequence is known to have good resolution between desired product and various aspartimide-dependent side products.[1] NN92[15–29] was synthesized on the AFPS to shorten the manual synthesis and shift the focus to the aspartimide-prone segment.

13.1 Synthesis of NN92[15–29] on the AFPS

The sequence TEQYVNVQKIVSHPY was synthesized on commercially available Novabiochem® NovaPEG Rink Amide resin (0.41 mmol/g, 208.3 mg, 85 μmol) using the standard AFPS protocol (4.2.1). Total synthesis time to afford resin-bound TEQYVNVQKIVSHPY was approximately 0.75 h. Cleavage of the peptidyl resin (11.4 mg, 4.7 μmol) according to Cleavage Protocol (Section 4.3) afforded the crude peptide as a colorless solid (85 % purity by LCMS [SI Figure 70]).

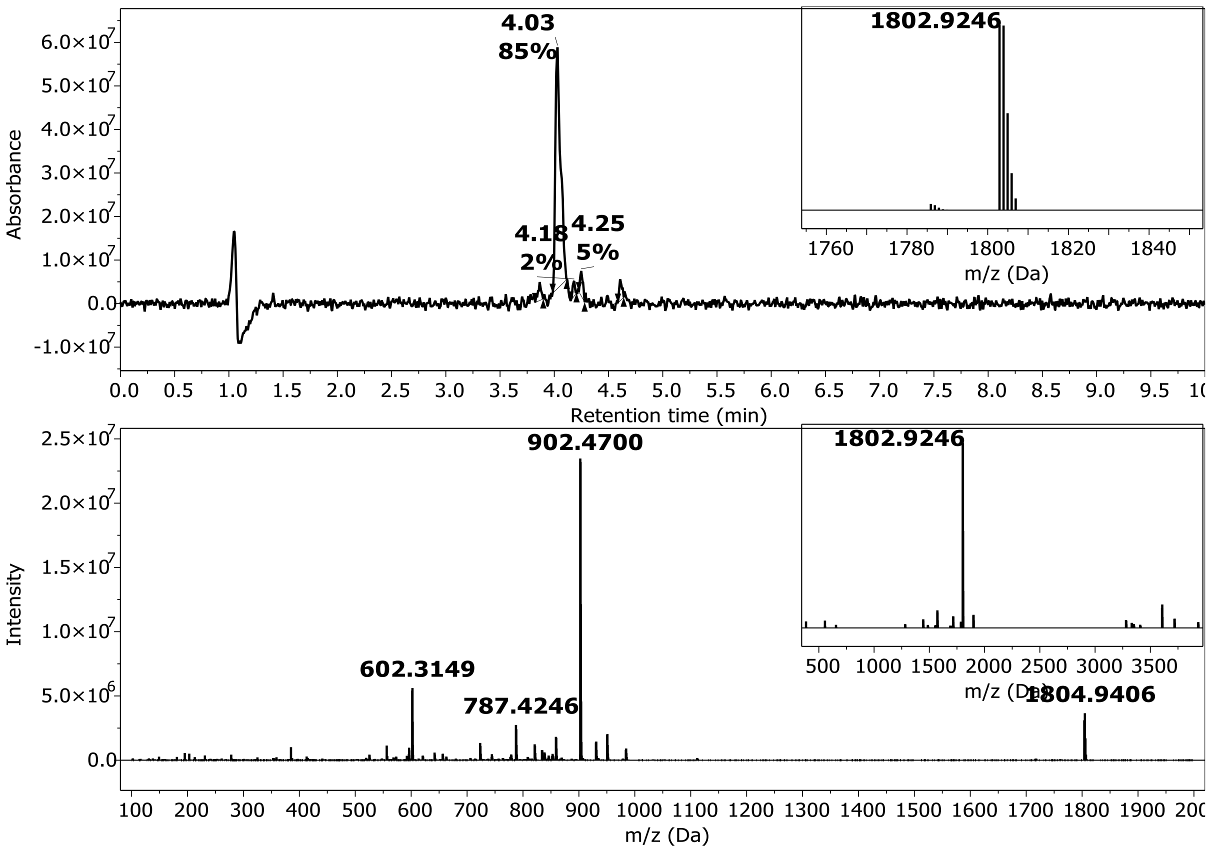

SI Figure 70: LCMS Profile of crude TEQYVNVQKIVSHPY. Absorbance chromatogram (λ = 214 nm) of TEQYVNVQKIVSHPY; R_t 4.03 min, 85 % purity. ESI-TOF spectrum found within $_{Rt}$ 2–8 min (insert: deconvoluted masses). Monoisotopic mass (ESI+) calcd. for $C_{82}H_{126}N_{22}O_{24}$ 1802.9315, found 1802.9246. LCMS Gradient A.

13.2 Standard synthesis of NN92[2–14] onto resin-bound NN92[15–29]

The sequence VVVGEHNLSQNDG was synthesized on previously synthesized, resin-bound TEQYVNVQKIVSHPY (11.2 mg, 4.6 µmol) using the reduced piperidine immobilized base-SPPS standard protocol (Section SI3). Cleavage of the peptidyl resin according to Cleavage Protocol (Section 4.3) afforded the crude aspartimide-containing peptide (2.4 mg, [SI Figure 71], and UHPLC [SI Figure 72]).

AA	m(AA) [mg]	Immob. base	Flow rate [mL/min]	Base temp. [°C]	Reactor temp. [°C]	Activator	t_{sw} [min]
3 x V	205.0	DIPA 8	V2: 0.4<br>V3: 0.4<br>V4: 0.6	90	90	HATU	V2: 8<br>V3: 8<br>V4: 4
2 x N	239.5	DIPA 8	0.6	90	90	HATU	4
2 x G	120.6	DIPA 8	0.6	90	90	HATU	4
E	88.7	DIPA 8	0.4	90	90	HATU	8
H(Boc)	94.5	DIPA 8	0.6	90	90	HATU	4

L	71.8	DIPA 8	0.6	90	90	HATU	4
S	77.7	DIPA 8	0.4	90	90	HATU	8
Q	121.3	DIPA 8	0.6	90	90	HATU	4
D	81.7	DIPA 8	0.6	90	90	HATU	4

Depro.	V(pip, 20%) [mL]	Flow rate [mL/min]	t_{fw1} [min]	t_{fw2} [min]	$t_{fw,final}$ [min]
Pip.	1.0	5	0.6	3.4	t_{fw2}

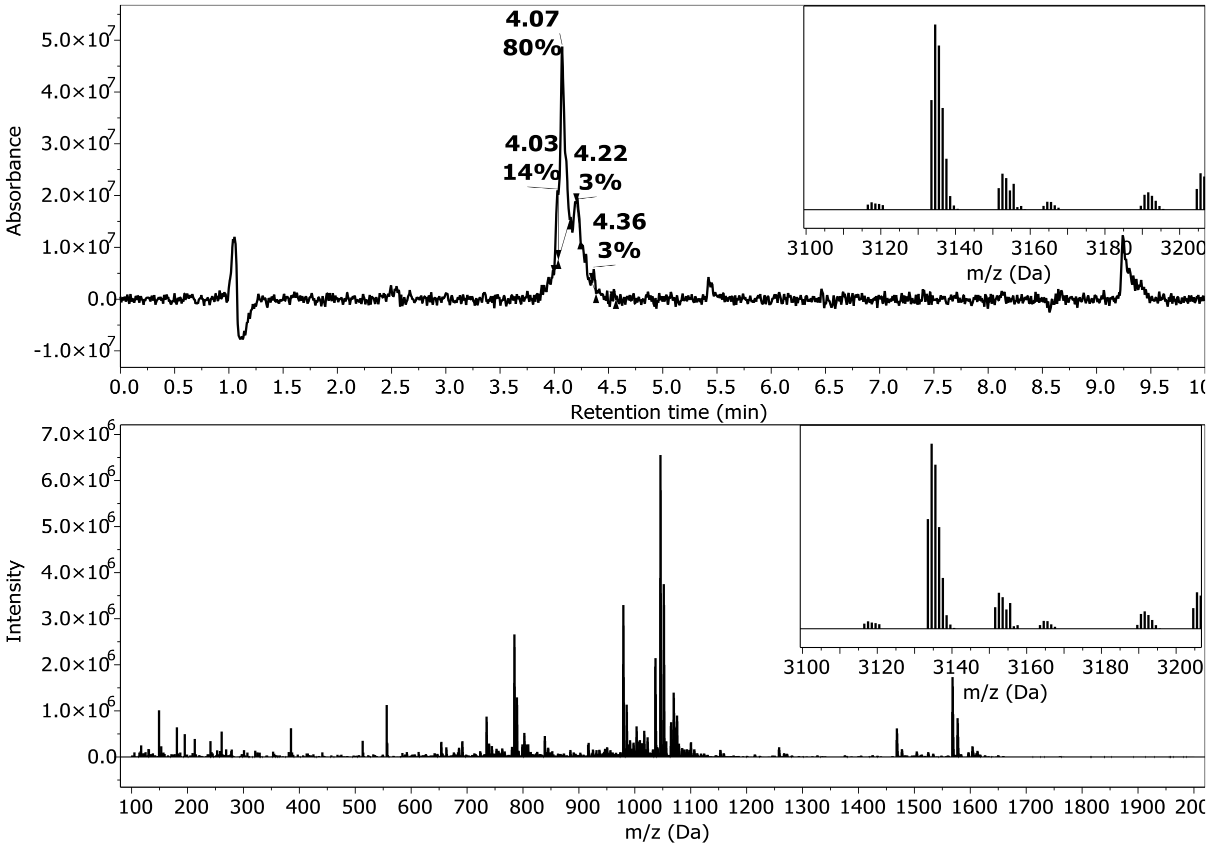

SI Figure 71: LCMS Profile of crude VVVGEHNLSQNDGTEQYVNVQKIVSHPY. Absorbance chromatogram (λ = 214 nm) of VVVGEHNLSQNDGTEQYVNVQKIVSHPY; Rt not found, 0 % purity. ESI-TOF spectrum found within Rt 2–8 min (insert: deconvoluted masses). Monoisotopic mass (ESI+) calcd. for $C_{138}H_{214}N_{40}O_{45}$ 3152.5687, found 3133.5460 (= aspartimide). LCMS Gradient A.

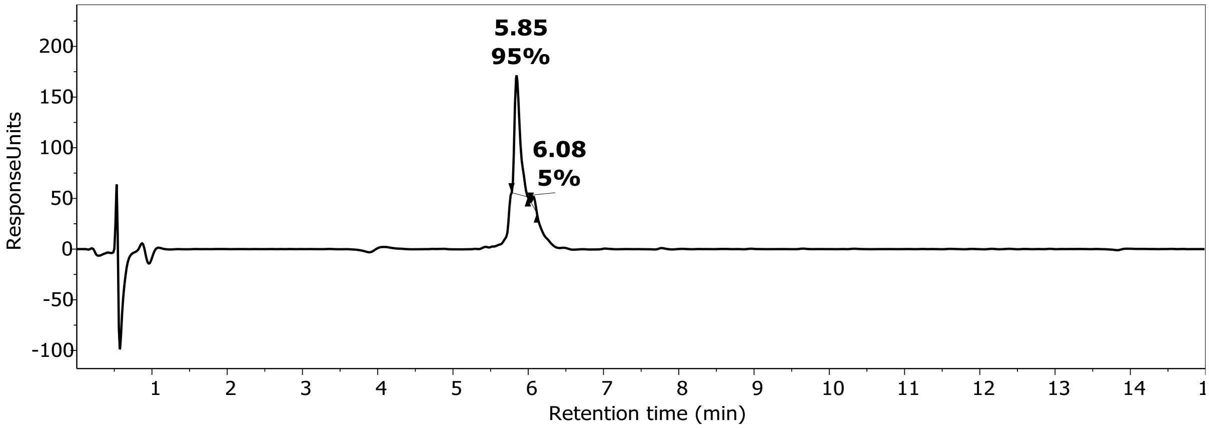

SI Figure 72: UHPLC profile of crude NN91[2–29]. Area Under Curve (AUC) at λ = 214 nm.

Synthesis of NN92[2–14] (VVVGEHNLSQNDG) was also attempted using the reduced piperidine immobilized base SPPS protocol (Section SI3) at 100 °C, but the target mass could not be identified. Synthesis was repeated with 25% dipropylamine in DMF as deprotection base at 90 °C and 110 °C, but again the desired mass could not be found. Instead, we exclusively detected the corresponding aspartimide mass.

14 Synthesis of aggregating sequences

14.1 JR-10

14.1.1 Synthesis with the conditions optimized for each individual amino acid

The sequence WFFTLISTIM was synthesized on Novabiochem® NovaPEG Rink Amide resin (0.41 mmol/g loading, 9.7 mg, 4.0 μmol) using the reduced piperidine immobilized base-SPPS standard protocol (Section SI3). Cleavage of the peptidyl resin according to Cleavage Protocol (Section 1.3) afforded the crude peptide (1.7 mg, 58 % purity by LCMS [SI Figure 73], 56% purity by UHPLC [SI Figure 74]).

AA	m(AA) [mg]	Immob. base	Flow rate [mL/min]	Base temp. [°C]	Reactor temp. [°C]	Activator	t_{sw} [min]
2 x F	156.1	DIPA 8	0.6	90	90	HATU	4
2 x T	160.7	DIPA 8	0.6	90	90	HATU	4
2 x I	140.7	DIPA 8	0.6	90	90	HATU	4
L	71.8	DIPA 8	0.4	90	90	HATU	8
S	76.2	DIPA 8	0.4	90	90	HATU	8
M	73.4	DIPA 8	0.6	90	90	HATU	4
W	106.1	DIPA 8	0.6	90	90	HATU	4
Depro.	V(pip, 20%) [mL]	Flow rate [mL/min]	t_{fw1} [min]	t_{fw2} [min]	$t_{fw,final}$ [min]		

Pip.	1.0	5	0.6	3.4	t_{fw2}

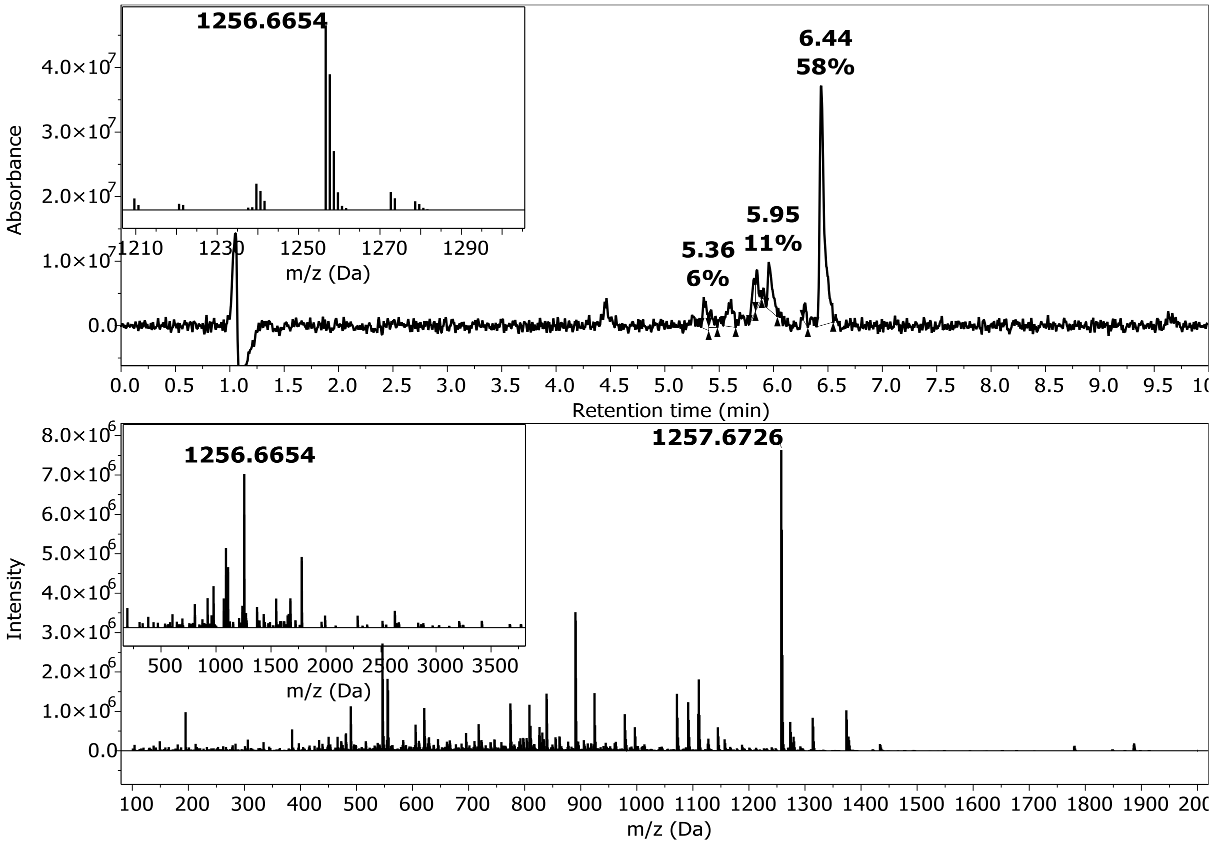

SI Figure 73: LCMS Profile of crude WFFTLISTIM. Absorbance chromatogram (λ = 214 nm) of WFFTLISTIM; R_t 6.44 min, 58% purity. ESI-TOF spectrum found within R_t 2–8 min (insert: deconvoluted masses). Monoisotopic mass (ESI+) calcd. for $C_{63}H_{92}N_{12}O_{13}S$ 1256.6628, found 1256.6654. LCMS Gradient A.

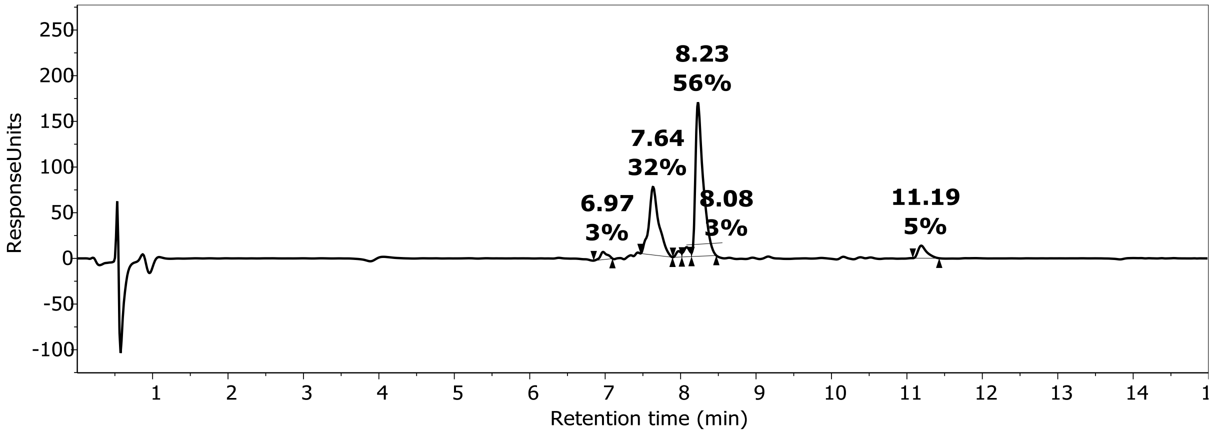

SI Figure 74: UHPLC profile of crude jR-10. Rt 8.23 min. 56% purity based on Area Under Curve (AUC) at λ = 214 nm.

14.1.2 100 °C reactor temperature for all amino acids

The sequence WFFTLISTIM was synthesized on Novabiochem® NovaPEG Rink Amide resin (0.41 mmol/g loading, 9.9 mg, 4.1 µmol) using the reduced piperidine immobilized base-SPPS standard protocol (Section SI3). Cleavage of the peptidyl resin according to Cleavage Protocol (Section 1.3) afforded the crude peptide (2.4 mg, 54 % purity by LCMS [SI Figure 75], 55% purity by UHPLC [SI Figure 76]).

AA	m(AA) [mg]	Immob. base	Flow rate [mL/min]	Base temp. [°C]	Reactor temp. [°C]	Activator	t_{sw} [min]
2 x F	155.1	DIPA 8	0.6	90	100	HATU	4
2 x T	159.7	DIPA 8	0.6	90	100	HATU	4
2 x I	142.0	DIPA 8	0.6	90	100	HATU	4
L	71.8	DIPA 8	0.4	90	100	HATU	8
S	77.4	DIPA 8	0.4	90	100	HATU	8
M	74.5	DIPA 8	0.6	90	100	HATU	4
W	104.7	DIPA 8	0.6	90	100	HATU	4

Depro.	V(pip, 20%) [mL]	Flow rate [mL/min]	t_{fw1} [min]	t_{fw2} [min]	$t_{fw,final}$ [min]
Pip.	1.0	5	0.6	3.4	t_{fw2}

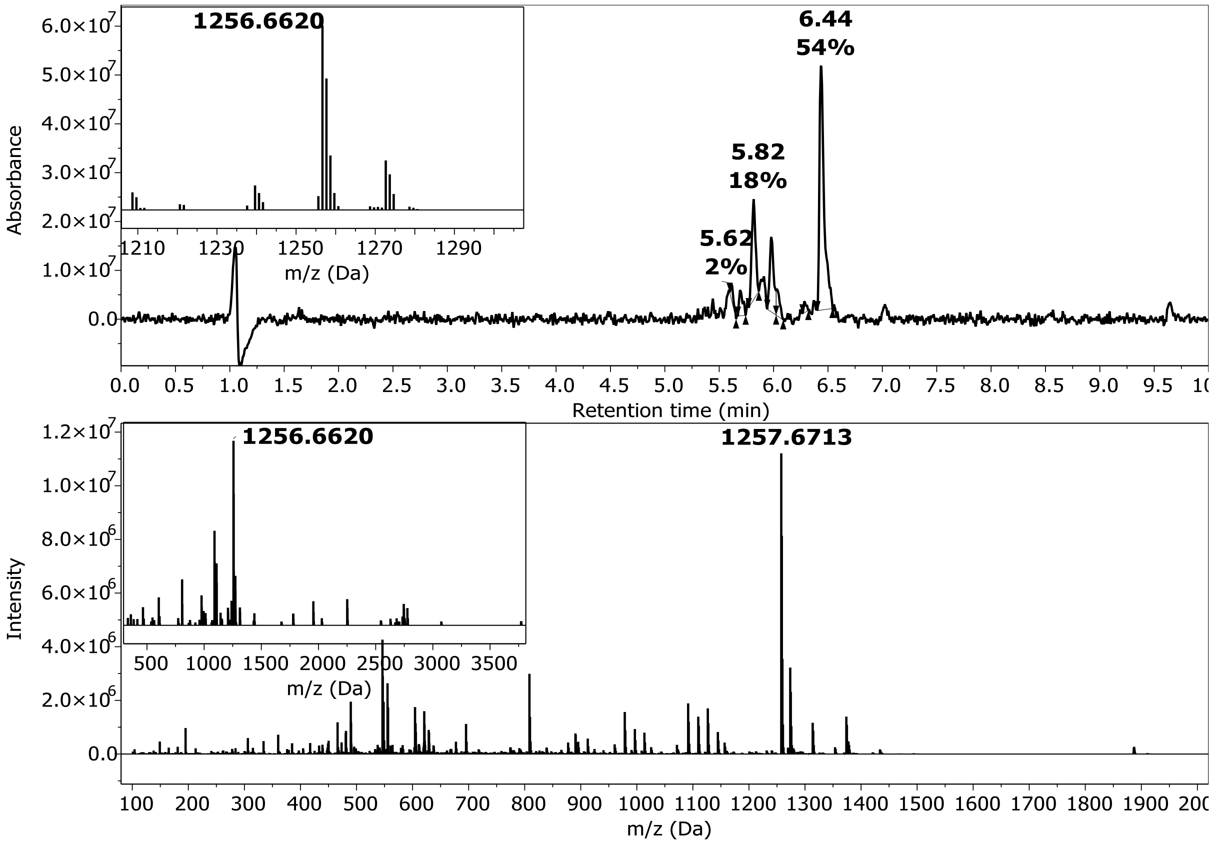

SI Figure 75: LCMS Profile of crude WFFTLISTIM. Absorbance chromatogram (λ = 214 nm) of WFFTLISTIM; R_t 6.44 min, 54% purity. ESI-TOF spectrum found within R_t 2–8 min (insert: deconvoluted masses). Monoisotopic mass (ESI+) calcd. for $C_{63}H_{92}N_{12}O_{13}S$ 1256.6628, found 1256.6620. LCMS Gradient A.

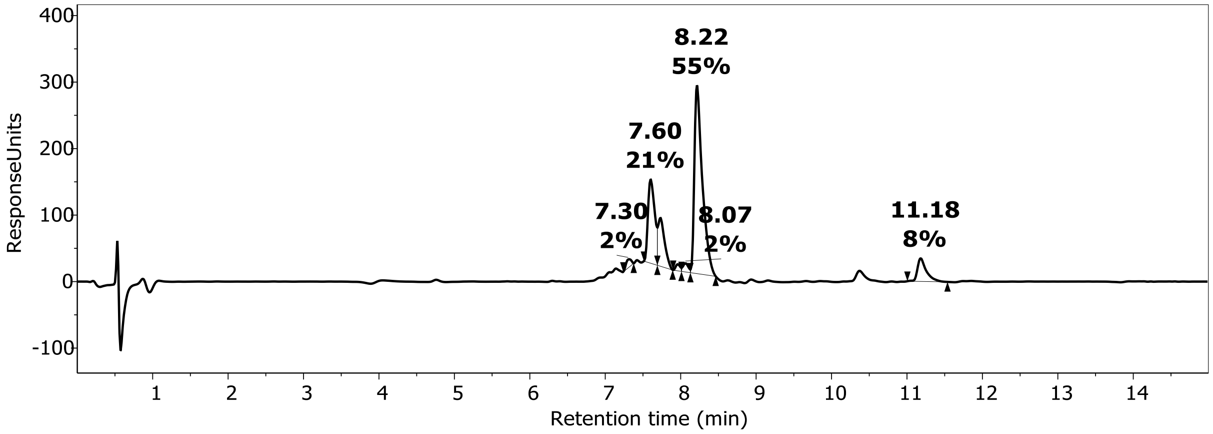

SI Figure 76: UHPLC profile of crude jR-10. R_t 8.22 min. 55% purity based on Area Under Curve (AUC) at λ = 214 nm.

14.1.3 110 °C reactor temperature for all amino acids

The sequence WFFTLISTIM was synthesized on Novabiochem® NovaPEG Rink Amide resin (0.41 mmol/g loading, 9.8 mg, 4.0 μmol) using the reduced piperidine immobilized base-SPPS standard protocol (Section SI3). Cleavage of the peptidyl resin according to Cleavage Protocol (Section 1.3) afforded the crude peptide (1.5 mg, 43 % purity by LCMS [SI Figure 77], 39% purity by UHPLC [SI Figure 78]).

AA	m(AA) [mg]	Immob. base	Flow rate [mL/min]	Base temp. [°C]	Reactor temp. [°C]	Activator	t_{sw} [min]
2 x F	155.4	DIPA 8	0.6	90	110	HATU	4
2 x T	160.1	DIPA 8	0.6	90	110	HATU	4
2 x I	140.5	DIPA 8	0.6	90	110	HATU	4
L	70.9	DIPA 8	0.4	90	110	HATU	8
S	76.7	DIPA 8	0.4	90	110	HATU	8
M	74.3	DIPA 8	0.6	90	110	HATU	4
W	104.4	DIPA 8	0.6	90	110	HATU	4
Depro.	V(pip, 20%) [mL]	Flow rate [mL/min]	t_{fw1} [min]	t_{fw2} [min]	$t_{fw,final}$ [min]		
Pip.	1.0	5	0.6	3.4	t_{fw2}		

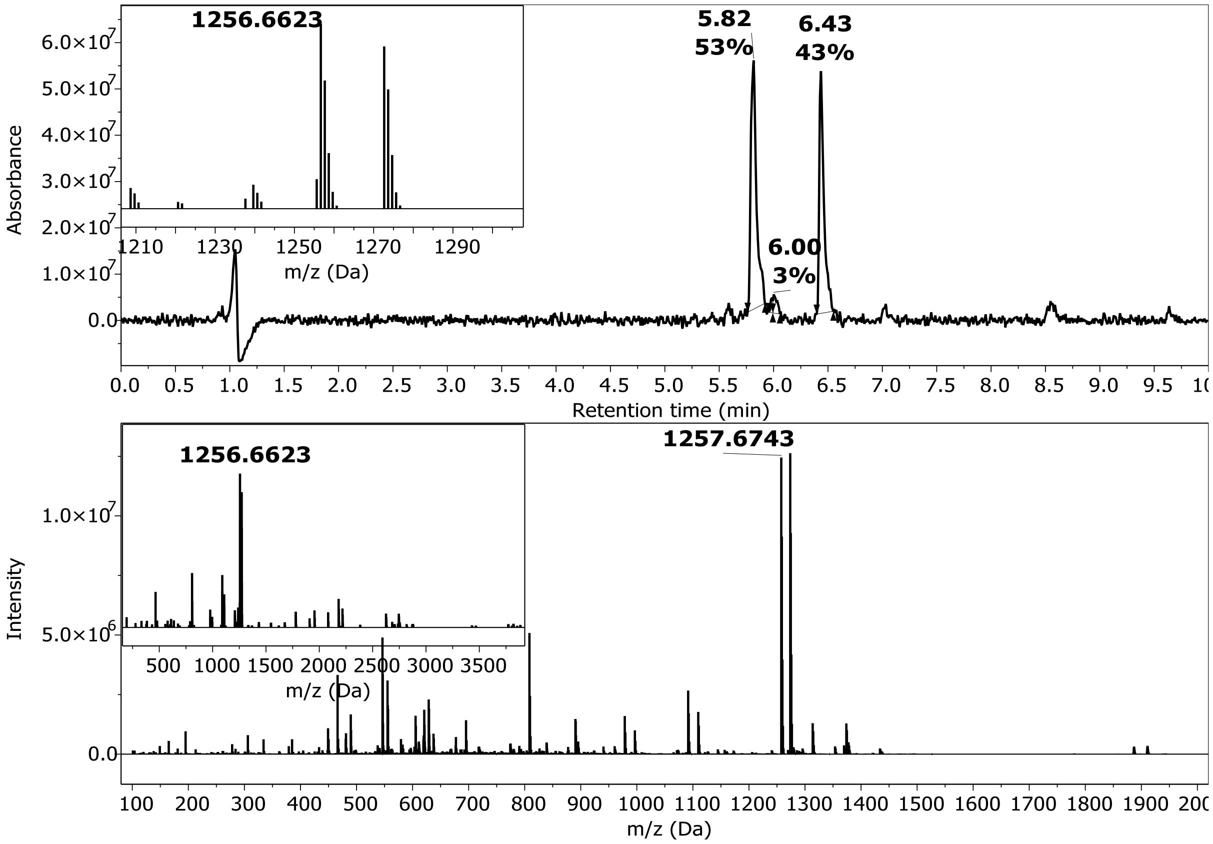

SI Figure 77: LCMS Profile of crude WFFTLISTIM. Absorbance chromatogram (λ = 214 nm) of WFFTLISTIM; R_t 6.43 min, 43% purity. ESI-TOF spectrum found within R_t 2–8 min (insert: deconvoluted masses). Monoisotopic mass (ESI+) calcd. for $C_{63}H_{92}N_{12}O_{13}S$ 1256.6628, found 1256.6623. LCMS Gradient A.

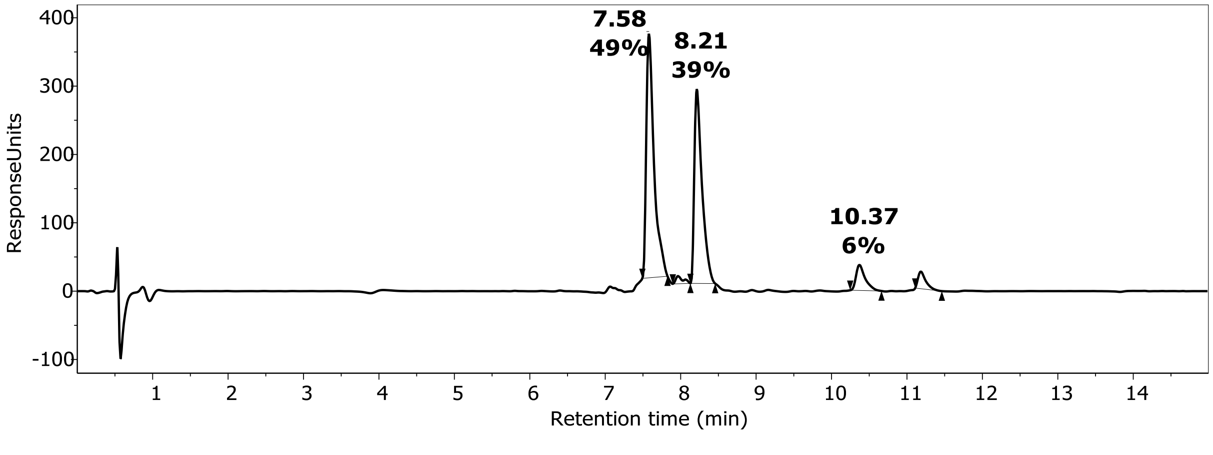

SI Figure 78: UHPLC profile of crude jR-10. R_t 8.21 min. 39% purity based on Area Under Curve (AUC) at λ = 214 nm.

14.1.4 90 °C synthesis with degassed solvent

The sequence WFFTLISTIM was synthesized on Novabiochem® NovaPEG Rink Amide resin (0.41 mmol/g loading, 9.8 mg, 4.0 μmol) using the reduced piperidine immobilized base-SPPS standard protocol

(Section SI3). Cleavage of the peptidyl resin according to Cleavage Protocol (Section 1.3) afforded the crude peptide (1.5 mg, 50 % purity by LCMS [SI Figure 79]).

AA	m(AA) [mg]	Immob. base	Flow rate [mL/min]	Base temp. [°C]	Reactor temp. [°C]	Activator	t_{sw} [min]
F[2]	78.1	DIPA 10	0.6	90	90	HATU	4
F[3]	78.1	DIPA 10	0.6	90	90	HATU	4
T[4]	79.2	DIPA 10	0.6	90	90	HATU	4
T[8]	79.2	DIPA 10	0.6	90	90	HATU	4
I[6]	70.9	DIPA 10	0.6	90	90	HATU	4
I[9]	70.9	DIPA 10	0.6	90	90	HATU	4
L	71.3	DIPA 10	0.4	90	90	HATU	8
S	75.7	DIPA 10	0.4	90	90	HATU	8
M	74.7	DIPA 10	0.6	90	90	HATU	4
W	105.9	DIPA 10	0.6	90	90	HATU	4
Depro.	V(pip, 20%) [mL]	Flow rate [mL/min]	t_{fw1} [min]	t_{fw2} [min]	$t_{fw,final}$ [min]		
Pip.	1.0	5	0.6	3.4	t_{fw2}		

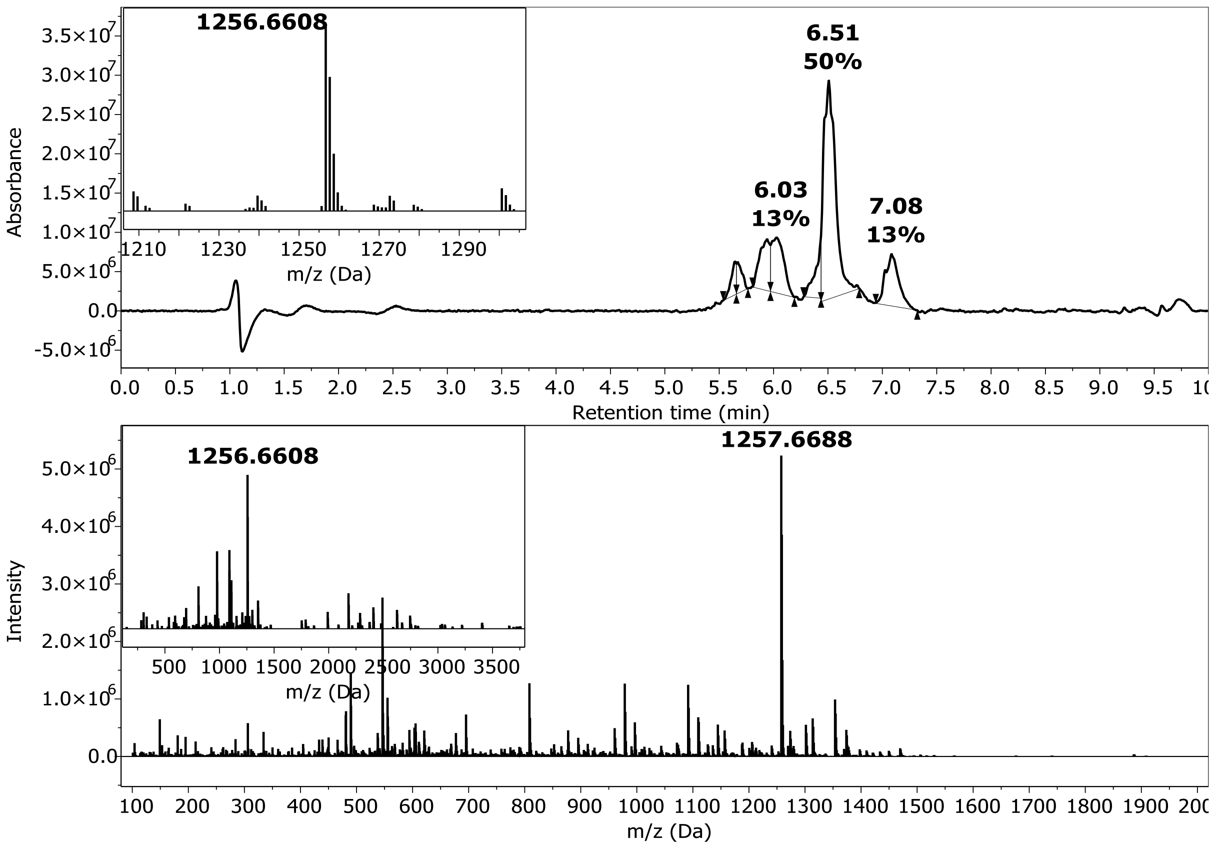

SI Figure 79: LCMS Profile of crude WFFTLISTIM. Absorbance chromatogram (λ = 214 nm) of WFFTLISTIM; R_t 6.51 min, 50% purity. ESI-TOF spectrum found within R_t 2–8 min (insert: deconvoluted masses). Monoisotopic mass (ESI+) calcd. for $C_{63}H_{92}N_{12}O_{13}S$ 1256.6628, found 1256.6608. LCMS Gradient A.

14.1.5 110 °C synthesis with degassed solvent

The sequence WFFTLISTIM was synthesized on Novabiochem® NovaPEG Rink Amide resin (0.41 mmol/g loading, 9.8 mg, 4.0 μmol) using the reduced piperidine immobilized base-SPPS standard protocol (Section SI3). Cleavage of the peptidyl resin according to Cleavage Protocol (Section 1.3) afforded the crude peptide (1.5 mg, 61% purity by LCMS [SI Figure 80]).

AA	m(AA) [mg]	Immob. base	Flow rate [mL/min]	Base temp. [°C]	Reactor temp. [°C]	Activator	t_{sw} [min]
F[2]	77.5	DIPA 11	0.6	90	110	HATU	4
F[3]	77.2	DIPA 11	0.6	90	110	HATU	4
T[4]	79.7	DIPA 11	0.6	90	110	HATU	4
T[8]	79.7	DIPA 11	0.6	90	110	HATU	4
I[6]	70.5	DIPA 11	0.6	90	110	HATU	4
I[9]	71.0	DIPA 11	0.6	90	110	HATU	4
L	71.9	DIPA 11	0.4	90	110	HATU	8

S	77.5	DIPA 11	0.4	90	110	HATU	8
M	75.5	DIPA 11	0.6	90	110	HATU	4
W	106.6	DIPA 11	0.6	90	110	HATU	4
Depro.	V(pip, 20%) [mL]	Flow rate [mL/min]	t_{fw1} [min]	t_{fw2} [min]	$t_{fw,final}$ [min]		
Pip.	1.0	5	0.6	3.4	t_{fw2}		

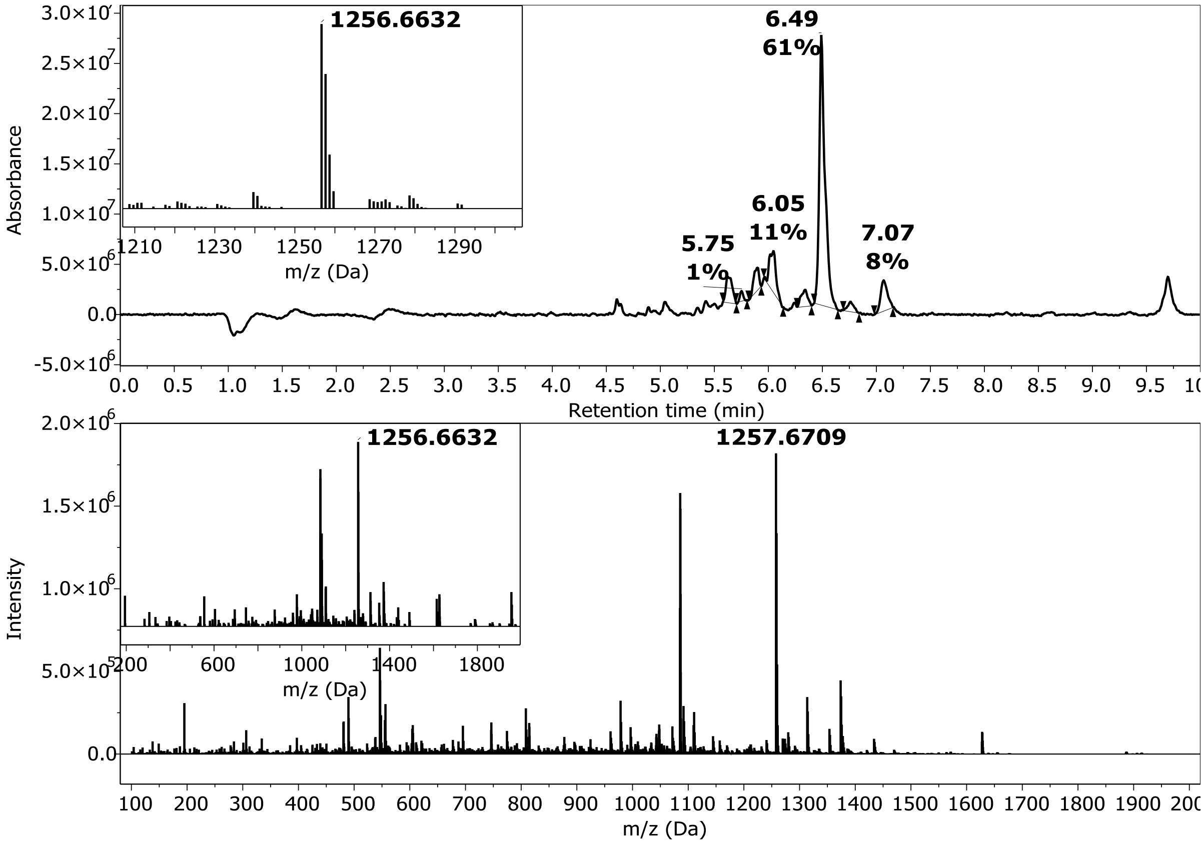

SI Figure 80: LCMS Profile of crude WFFTLISTIM. Absorbance chromatogram (λ = 214 nm) of WFFTLISTIM; R_t 6.49 min, 61% purity. ESI-TOF spectrum found within R_t 2–8 min (insert: deconvoluted masses). Monoisotopic mass (ESI+) calcd. for $C_{63}H_{92}N_{12}O_{13}S$ 1256.6628, found 1256.6632. LCMS Gradient A.

14.2 Amyloid-β42 [27–42]

14.2.1 Synthesis with the conditions optimized for each individual amino acid

The sequence KGAIIGLMVGGVVIA was synthesized on Novabiochem® NovaPEG Rink Amide resin (0.41 mmol/g loading, 11.3 mg, 4.6 μmol) using the reduced piperidine immobilized base-SPPS standard protocol (Section SI3). Cleavage of the peptidyl resin according to Cleavage Protocol (Section 1.3) afforded the crude peptide (1.8 mg, 47% purity by LCMS [SI Figure 81], 39% purity by UHPLC [SI Figure 82]).

AA	m(AA) [mg]	Immob. base	Flow rate [mL/min]	Base temp. [°C]	Reactor temp. [°C]	Activator	t_{sw} [min]
G[2]	59.5	DIPA 10	0.6	90	90	HATU	4
G[6]	60.5	DIPA 10	0.6	90	90	HATU	8
G[10]	60.4	DIPA 10	0.6	90	90	HATU	4
G[11]	61.2	DIPA 10	0.6	90	90	HATU	4
A[3]	66.4	DIPA 10	0.6	90	90	HATU	4
A[15]	67.0	DIPA 10	0.6	90	90	HATU	4
I[4]	72.1	DIPA 10	0.4	90	90	HATU	8
I[5]	72.3	DIPA 10	0.6	90	90	HATU	4
I[14]	72.5	DIPA 10	0.6	90	90	HATU	4
V[9]	68.4	DIPA 10	0.6	90	90	HATU	4
V[12]	68.3	DIPA 10	0.4	90	90	HATU	8
V[13]	68.2	DIPA 10	0.4	90	90	HATU	8
K	93.4	DIPA 10	0.6	90	90	HATU	4
L	71.0	DIPA 10	0.6	90	90	HATU	4
M	74.7	DIPA 10	0.6	90	90	HATU	4
Depro.	V(pip, 20%) [mL]	Flow rate [mL/min]	t_{fw1} [min]	t_{fw2} [min]	$t_{fw,final}$ [min]		
Pip.	1.0	5	0.6	3.4	t_{fw2}		

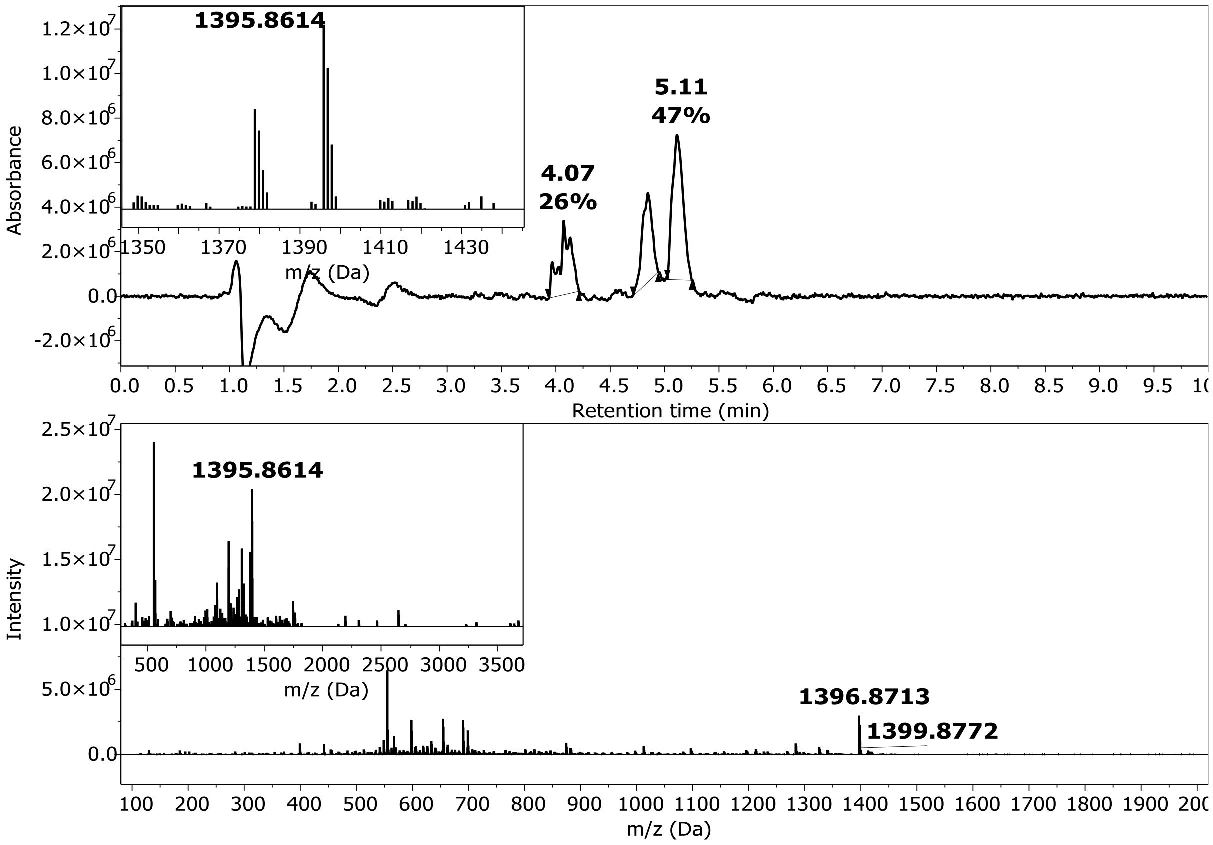

SI Figure 81: LCMS Profile of crude KGAIIGLMVGGVVIA. Absorbance chromatogram (λ = 214 nm) of KGAIIGLMVGGVVIA; R_t 5.11 min, 47% purity. ESI-TOF spectrum found within R_t 2–8 min (insert: deconvoluted masses). Monoisotopic mass (ESI+) calcd. for $C_{64}H_{117}N_{17}O_{15}S$ 1395.8636, found 1395.8614. LCMS gradient A.

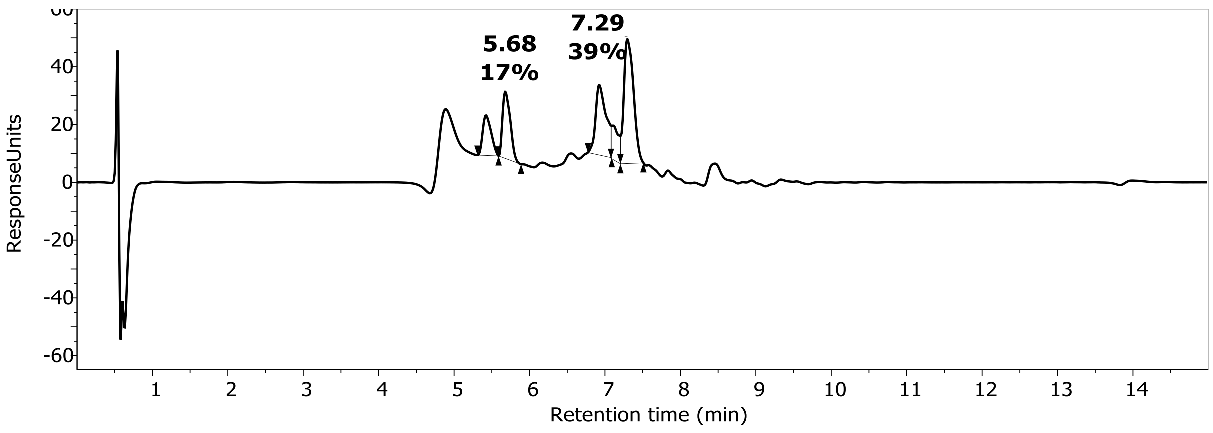

SI Figure 82: UHPLC profile of crude Amyloid-β. R_t 7.29 min. 39% purity based on Area Under Curve (AUC) at λ = 214 nm.

14.2.2 110 °C reactor temperature for all amino acids

The sequence KGAIIGLMVGGVVIA was synthesized on Novabiochem® NovaPEG Rink Amide resin (0.41 mmol/g loading, 11.3 mg, 4.6 µmol) using the reduced piperidine immobilized base-SPPS standard

protocol (Section SI3). Cleavage of the peptidyl resin according to Cleavage Protocol (Section 1.3) afforded the crude peptide (1.5 mg, 55% purity by LCMS [SI Figure 83], 42% purity by UHPLC [SI Figure 84]).

AA	m(AA) [mg]	Immob. base	Flow rate [mL/min]	Base temp. [°C]	Reactor temp. [°C]	Activator	t_{sw} [min]
G[2]	59.4	DIPA 10	0.6	90	110	HATU	4
G[6]	61.3	DIPA 10	0.6	90	110	HATU	8
G[10]	61.0	DIPA 10	0.6	90	110	HATU	4
G[11]	60.2	DIPA 10	0.6	90	110	HATU	4
A[3]	67.8	DIPA 10	0.6	90	110	HATU	4
A[15]	66.6	DIPA 10	0.6	90	110	HATU	4
I[4]	71.5	DIPA 10	0.4	90	110	HATU	8
I[5]	72.2	DIPA 10	0.6	90	110	HATU	4
I[14]	70.0	DIPA 10	0.6	90	110	HATU	4
V[9]	69.0	DIPA 10	0.6	90	110	HATU	4
V[12]	68.7	DIPA 10	0.4	90	110	HATU	8
V[13]	68.8	DIPA 10	0.4	90	110	HATU	8
K	94.9	DIPA 10	0.6	90	110	HATU	4
L	70.5	DIPA 10	0.6	90	110	HATU	4
M	75.0	DIPA 10	0.6	90	110	HATU	4
Depro.	V(pip, 20%) [mL]	Flow rate [mL/min]	t_{fw1} [min]	t_{fw2} [min]	$t_{fw,final}$ [min]		
Pip.	1.0	5	0.6	3.4	t_{fw2}		

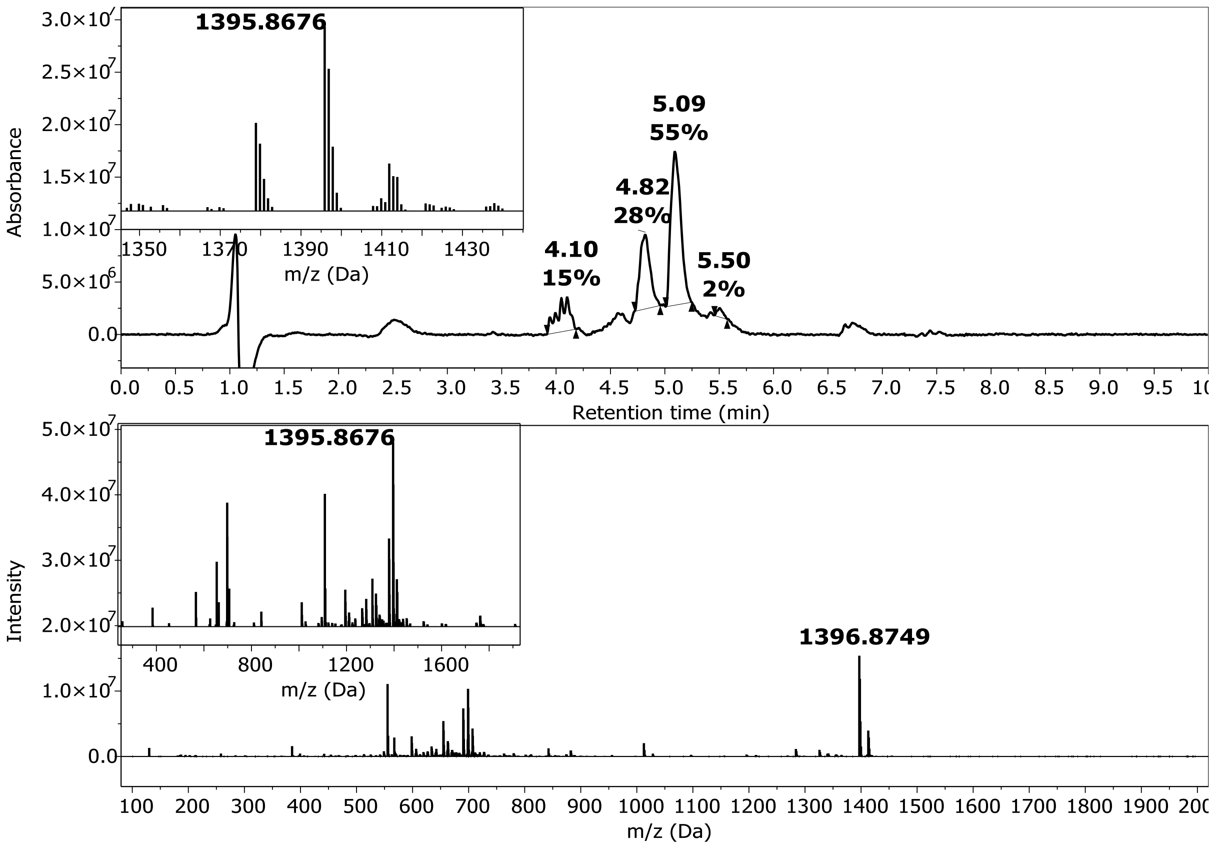

SI Figure 83: LCMS Profile of crude KGAIIGLMVGGVVIA. Absorbance chromatogram (λ = 214 nm) of KGAIIGLMVGGVVIA; R_t 5.09 min, 55% purity. ESI-TOF spectrum found within R_t 2–8 min (insert: deconvoluted masses). Monoisotopic mass (ESI+) calcd. for $C_{64}H_{117}N_{17}O_{15}S$ 1395.8636, found 1395.8676. LCMS gradient A.

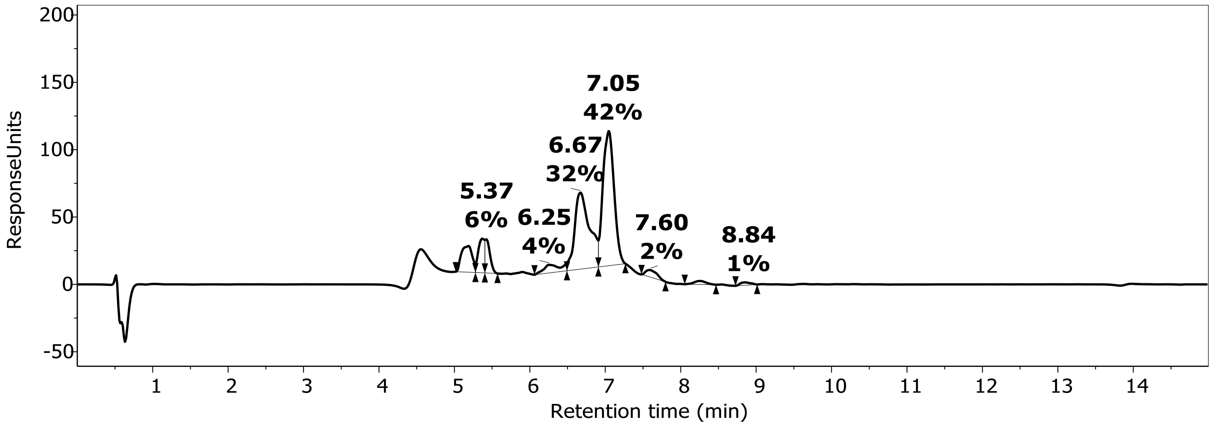

SI Figure 84: UHPLC profile of crude Amyloid-β. R_t 7.05 min. 42% purity based on Area Under Curve (AUC) at λ = 214 nm.

14.2.3 110°C synthesis with degassed solvent

The sequence KGAIIGLMVGGVVIA was synthesized on Novabiochem® NovaPEG Rink Amide resin (0.41 mmol/g loading, 10.5 mg, 4.6 μmol) using the reduced piperidine immobilized base-SPPS standard protocol (Section SI3). Cleavage of the peptidyl resin according to Cleavage Protocol (Section 1.3) afforded the crude peptide (1.3 mg, 64% purity by LCMS [SI Figure 85], 79% purity by UHPLC [SI Figure 86]).

AA	m(AA) [mg]	Immob. base	Flow rate [mL/min]	Base temp. [°C]	Reactor temp. [°C]	Activator	t_{sw} [min]
G[2]	60.3	DIPA 11	0.6	90	110	HATU	4
G[6]	61.2	DIPA 11	0.6	90	110	HATU	8
G[10]	59.9	DIPA 11	0.6	90	110	HATU	4
G[11]	60.6	DIPA 11	0.6	90	110	HATU	4
A[3]	67.0	DIPA 11	0.6	90	110	HATU	4
A[15]	66.4	DIPA 11	0.6	90	110	HATU	4
I[4]	72.0	DIPA 11	0.4	90	110	HATU	8
I[5]	70.9	DIPA 11	0.6	90	110	HATU	4
I[14]	71.6	DIPA 11	0.6	90	110	HATU	4
V[9]	67.9	DIPA 11	0.6	90	110	HATU	4
V[12]	69.7	DIPA 11	0.4	90	110	HATU	8
V[13]	68.5	DIPA 11	0.4	90	110	HATU	8
K	94.5	DIPA 11	0.6	90	110	HATU	4
L	71.1	DIPA 11	0.6	90	110	HATU	4
M	75	DIPA 11	0.6	90	110	HATU	4
Depro.	V(pip, 20%) [mL]	Flow rate [mL/min]	t_{fw1} [min]	t_{fw2} [min]	$t_{fw,final}$ [min]		
Pip.	1.0	5	0.6	3.4	t_{fw2}		

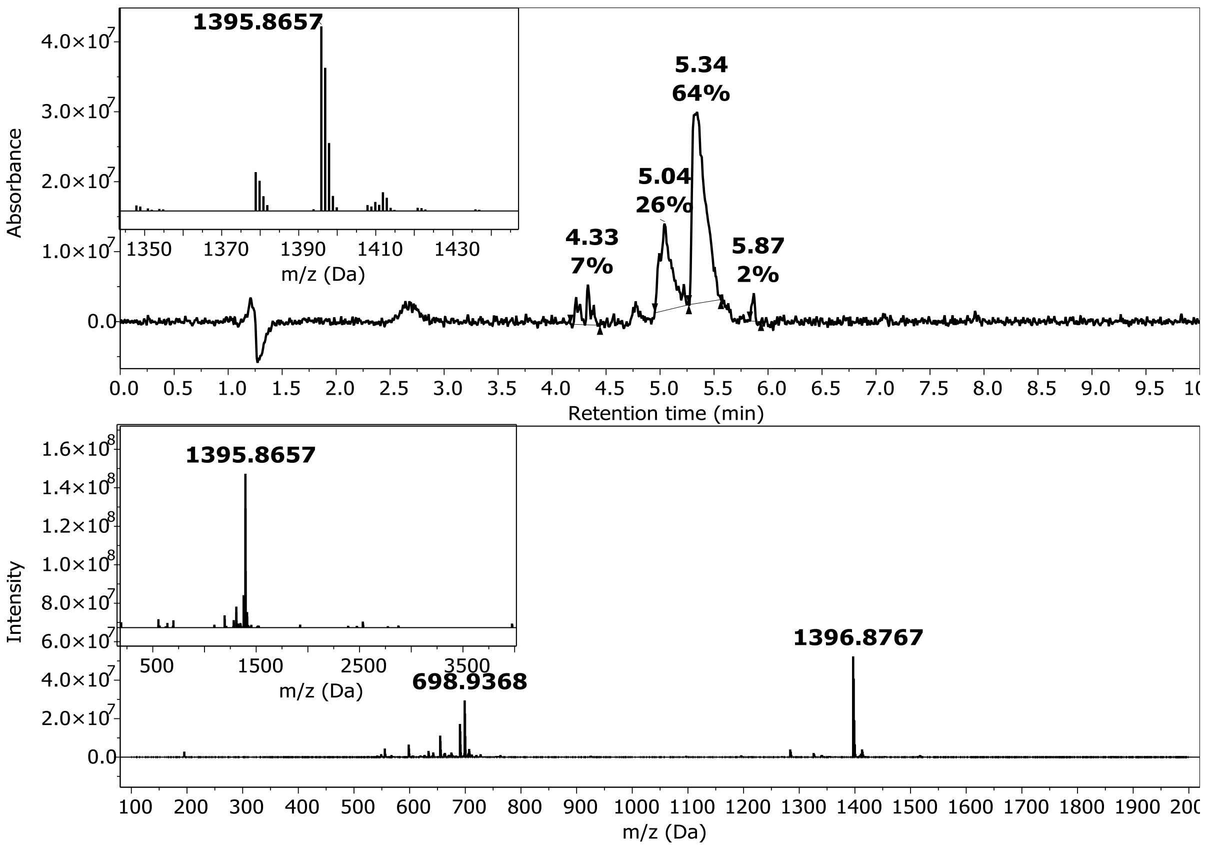

SI Figure 85: LCMS Profile of crude KGAIIGLMVGGVVIA. Absorbance chromatogram (λ = 214 nm) of KGAIIGLMVGGVVIA; R_t 5.34 min, 64% purity. ESI-TOF spectrum found within R_t 2–8 min (insert: deconvoluted masses). Monoisotopic mass (ESI+) calcd. for $C_{64}H_{117}N_{17}O_{15}S$ 1395.8636, found 1395.8657. LCMS gradient A.

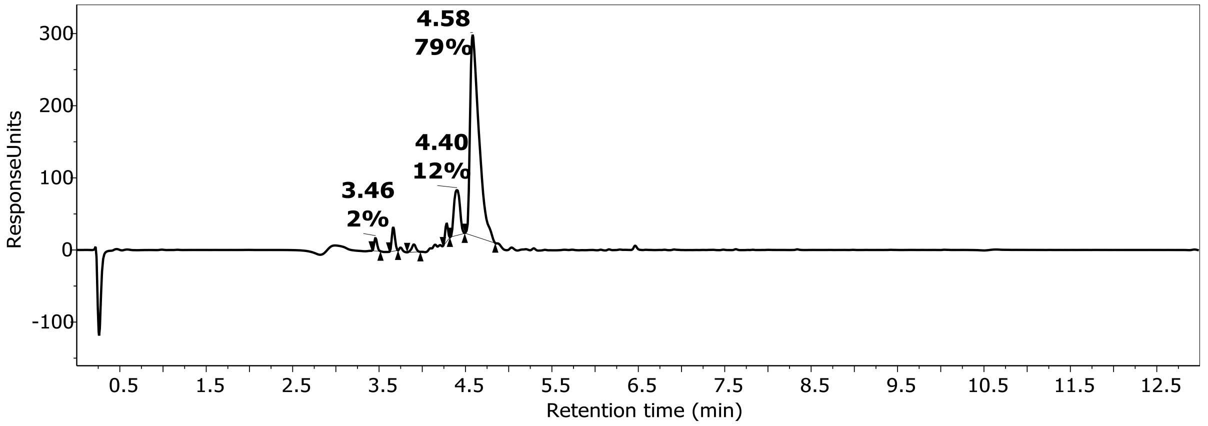

SI Figure 86: UHPLC profile of crude Amyloid β. R_t 4.58 min. 79% purity based on Area Under Curve (AUC) at λ = 214 nm.

14.3 α-synuclein [66–82].

14.3.1 Synthesis with the conditions optimized for each individual amino acid

The sequence VGGAVVTGVTAVAQKTV was synthesized on Novabiochem® NovaPEG Rink Amide resin (0.41 mmol/g loading, 10.7 mg, 4.4 μmol) using the reduced piperidine immobilized base-SPPS standard protocol (Section SI3). Cleavage of the peptidyl resin according to Cleavage Protocol (Section 1.3)

afforded the crude peptide (1.5 mg, 40% purity by LCMS [SI Figure 87], 55% purity by UHPLC [SI Figure 88]).

AA	m(AA) [mg]	Immob. base	Flow rate [mL/min]	Base temp. [°C]	Reactor temp. [°C]	Activator	t_{sw} [min]
V[1]	67.4	DIPA 10	0.6	90	90	HATU	4
V[5]	68.8	DIPA 10	0.4	90	90	HATU	8
V[6]	68.6	DIPA 10	0.6	90	90	HATU	4
V[9]	67.6	DIPA 10	0.6	90	90	HATU	4
V[12]	67.5	DIPA 10	0.6	90	90	HATU	4
V[17]	68.8	DIPA 10	0.4	90	90	HATU	8
G[2]	59.0	DIPA 10	0.6	90	90	HATU	4
G[3]	61.0	DIPA 10	0.6	90	90	HATU	4
G[8]	59.4	DIPA 10	0.6	90	90	HATU	4
A[4]	66.9	DIPA 10	0.6	90	90	HATU	4
A[11]	65.4	DIPA 10	0.6	90	90	HATU	4
A[13]	65.9	DIPA 10	0.6	90	90	HATU	4
T[7]	78.4	DIPA 10	0.6	90	90	HATU	4
T[10]	80.5	DIPA 10	0.6	90	90	HATU	4
T[16]	81.0	DIPA 10	0.6	90	90	HATU	4
Q	121.8	DIPA 10	0.6	90	90	HATU	4
K	93.7	DIPA 10	0.6	90	90	HATU	4
Depro.	V(pip, 20%) [mL]	Flow rate [mL/min]	t_{fw1} [min]	t_{fw2} [min]	$t_{fw,final}$ [min]		
Pip.	1.0	5	0.6	3.4	t_{fw2}		

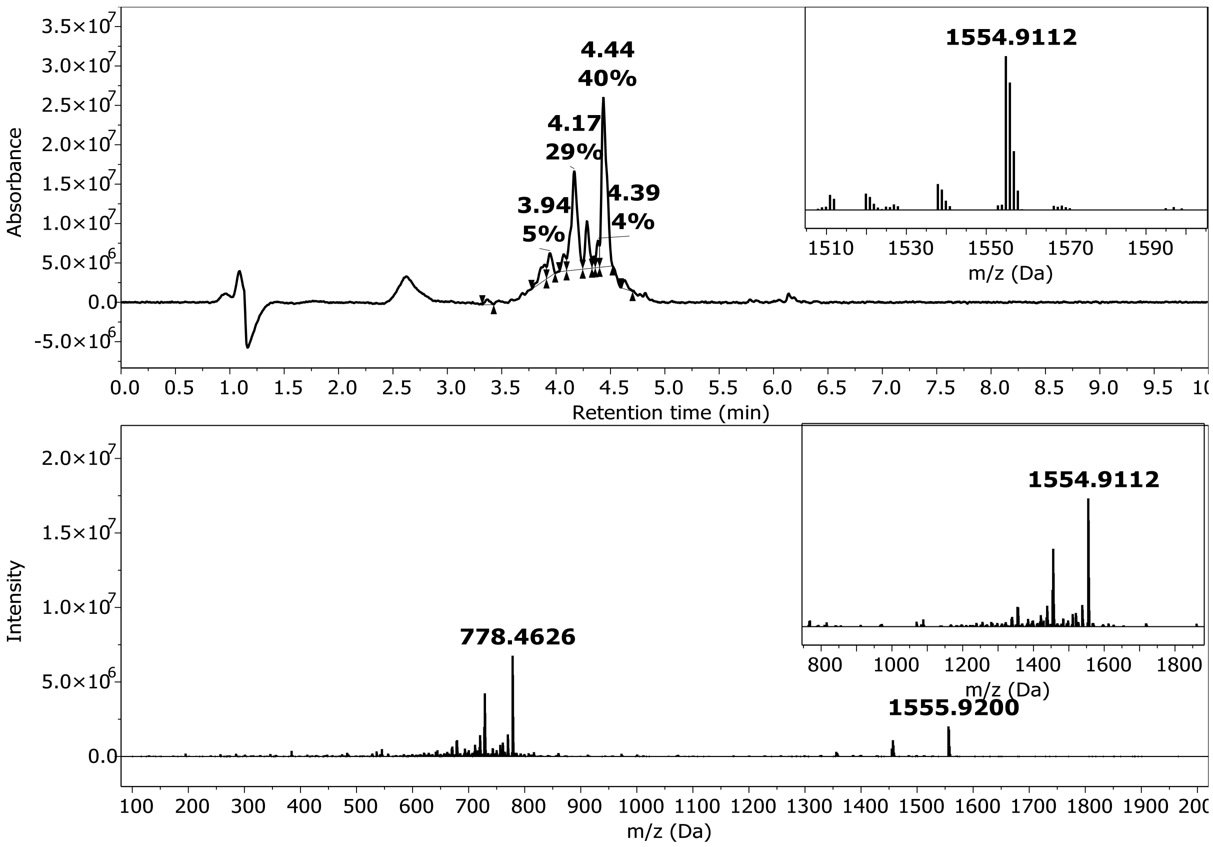

SI Figure 87: LCMS Profile of crude VGGAVVTGVTAVAQKTV. Absorbance chromatogram (λ = 214 nm) of VGGAVVTGVTAVAQKTV; R_t 4.44 min, 40% purity. ESI-TOF spectrum found within R_t 2–8 min (insert: deconvoluted masses). Monoisotopic mass (ESI+) calcd. for $C_{68}H_{122}N_{20}O_{21}$ 1554.9093, found 1554.9112. LCMS gradient A.

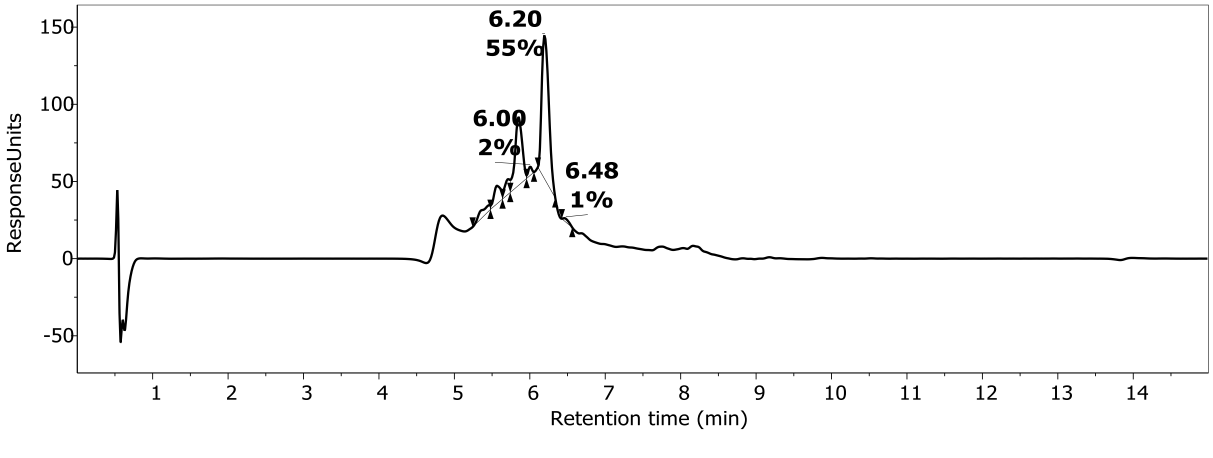

SI Figure 88: UHPLC profile of crude α-Synuclein. R_t 6.20 min. 55% purity based on Area Under Curve (AUC) at λ = 214 nm.

14.3.2 110°C reactor temperature for all amino acids

The sequence VGGAVVTGVTAVAQKTV was synthesized on Novabiochem® NovaPEG Rink Amide resin (0.41 mmol/g loading, 11.3 mg, 4.6 μmol) using the reduced piperidine immobilized base-SPPS standard protocol (Section SI3). Cleavage of the peptidyl resin according to Cleavage Protocol (Section 1.3)

afforded the crude peptide (1.7 mg, 59% purity by LCMS [SI Figure 89], 67% purity by UHPLC [SI Figure 90]).

AA	m(AA) [mg]	Immob. base	Flow rate [mL/min]	Base temp. [°C]	Reactor temp. [°C]	Activator	t_{sw} [min]
V[1]	67.4	DIPA 10	0.6	90	110	HATU	4
V[5]	68.8	DIPA 10	0.4	90	110	HATU	8
V[6]	68.6	DIPA 10	0.6	90	110	HATU	4
V[9]	67.6	DIPA 10	0.6	90	110	HATU	4
V[12]	67.5	DIPA 10	0.6	90	110	HATU	4
V[17]	68.8	DIPA 10	0.4	90	110	HATU	8
G[2]	59.0	DIPA 10	0.6	90	110	HATU	4
G[3]	61.0	DIPA 10	0.6	90	110	HATU	4
G[8]	59.4	DIPA 10	0.6	90	110	HATU	4
A[4]	66.9	DIPA 10	0.6	90	110	HATU	4
A[11]	65.4	DIPA 10	0.6	90	110	HATU	4
A[13]	65.9	DIPA 10	0.6	90	110	HATU	4
T[7]	78.4	DIPA 10	0.6	90	110	HATU	4
T[10]	80.5	DIPA 10	0.6	90	110	HATU	4
T[16]	81.0	DIPA 10	0.6	90	110	HATU	4
Q	121.8	DIPA 10	0.6	90	110	HATU	4
K	93.7	DIPA 10	0.6	90	110	HATU	4
Depro.	V(pip, 20%) [mL]	Flow rate [mL/min]	t_{fw1} [min]	t_{fw2} [min]	$t_{fw,final}$ [min]		
Pip.	1.0	5	0.6	3.4	t_{fw2}		

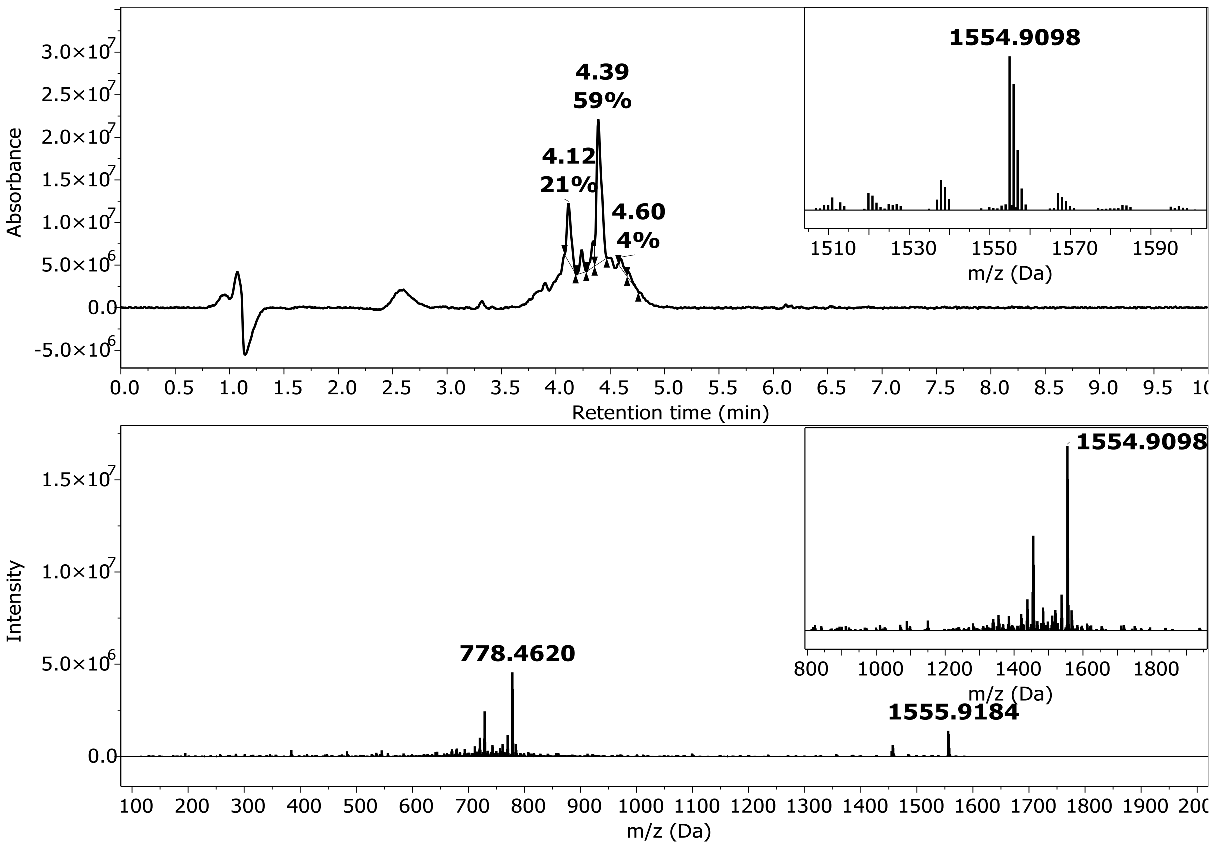

SI Figure 89: LCMS Profile of crude VGGAVVTGVTAVAQKTV. Absorbance chromatogram (λ = 214 nm) of VGGAVVTGVTAVAQKTV; R_t 4.39 min, 59% purity. ESI-TOF spectrum found within R_t 2–8 min (insert: deconvoluted masses). Monoisotopic mass (ESI+) calcd. for $C_{68}H_{122}N_{20}O_{21}$ 1554.9093, found 1554.9098. LCMS gradient A.

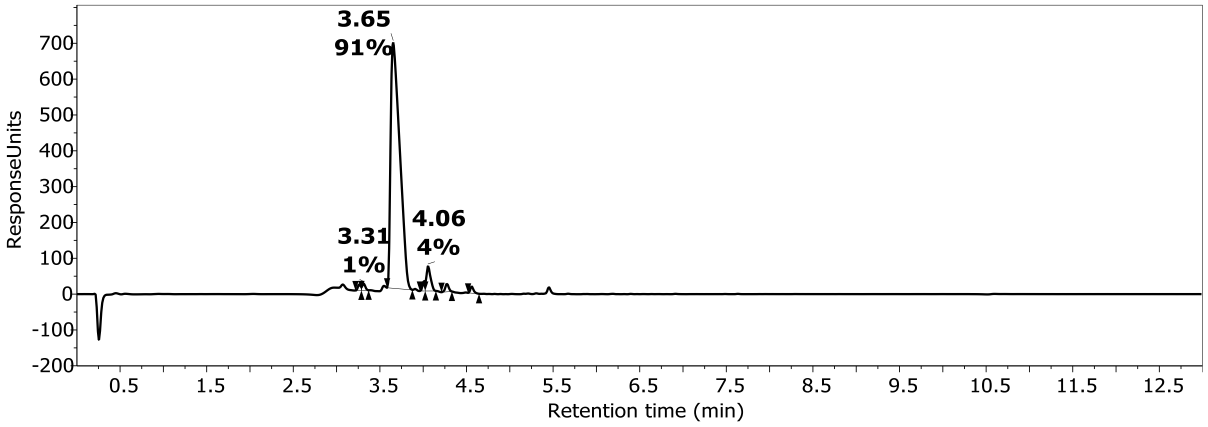

SI Figure 90: UHPLC profile of crude α-Synuclein. R_t 6.20 min. 67% purity based on Area Under Curve (AUC) at λ = 214 nm.

15 References

1. Hartrampf, N. *et al.* Synthesis of proteins by automated flow chemistry. *Science* **368**, 980–987 (2020).

2. Esteve, F., Porcar, R., Luis, S. V., Altava, B. & García-Verdugo, E. Continuous Flow Processes as an Enabling Tool for the Synthesis of Constrained Pseudopeptidic Macrocycles. *J. Org. Chem.* **87**, 3519–3528 (2022).
